# Recent Progress on Layered Sn and Pb-Based Mono Chalcogenides: Synthesis, Structure, Optical, and Thermoelectric Properties and Related Applications

**DOI:** 10.3390/nano14181530

**Published:** 2024-09-20

**Authors:** Safwan Rahman, Razia Khan Sharme, Mauricio Terrones, Mukti M. Rana

**Affiliations:** 1 Adamjee Cantonment College, Dhaka 1206, Bangladesh; safwansamin2005@gmail.com; 2 Division of Physics, Engineering, Mathematics, Delaware State University, Dover, DE 19901, USA; khansharme@gmail.com; 3 Department of Physics, Chemistry and Materials Science & Engineering, Pennsylvania State University, University Park, PA 16802, USA; mut11@psu.edu; 4 Optical Science Center for Applied Research (OSCAR) and Research on Nanomaterial-Based Integrated Circuits and Electronics (NICE), Delaware State University, Dover, DE 19901, USA

**Keywords:** IVA-VIA dichalcogenides, 2D materials, IVA-VIA compounds, thermoelectric properties, photodetector

## Abstract

The research on two-dimensional materials has gained significant traction due to their potential for thermoelectric, optical, and other properties. The development of two-dimensional (2D) nanostructured-based TE generators and photodetectors has shown promising results. Over the years, researchers have played a crucial role in advancing this field, enhancing the properties of 2D materials through techniques such as doping, alloying, and various growth methods. Among these materials, black phosphorus, transition metal dichalcogenides, graphene, and IVA-VIA compounds stand out for their remarkable electronic, mechanical, and optical properties. This study presents a comprehensive review of the progress in the field, focusing on IVA-VIA compounds and their applications in TE and photodetector technologies. We summarize recent advancements in enhancing these materials’ TE and optical properties and provide an overview of various synthesis techniques for their fabrication. Additionally, we highlight their potential applications as photodetectors in the infrared spectrum. This comprehensive review aims to equip researchers with a deep understanding of the TE and optical properties of 2DMs and their potential applications and to inspire further advancements in this field of research.

## 1. Introduction

Materials in which the displacement of electrons and phonons are confined in one dimension are generally referred to as two-dimensional (2D) materials (2DMs). These materials have sparked much interest among the scientific community due to their unique and fascinating features [1]. Additionally, these materials can be infinitely extended in length and width and can be a single atom thick, called mono layers, or a few layers thick. Atomically thin (with thickness < 1 nm) layers of these materials with lateral lengths up to several inches can now be produced [2]. 2DMs exhibit unique thermal, optical, and mechanical properties, making them promising for many technological and scientific applications. Another important application of these devices is in the field of TE generators (TEGs). TEGs directly convert heat, including solar energy or waste heat, into electrical power, making them a crucial candidate for sustainable energy development [3,4]. TEGs offer one of the most important sources of clean energy and have many applications in many areas, including sensors, cooling devices, and power generators. This potential for practical applications underscores the importance of research in this field [5,6]. While graphene is the most used 2DM for many applications, it is unsuitable for photodetection in ultra-violet (UV) and near-infrared (NIR) spectra because of its zero energy bandgap [7]. Transition metal dichalcogenides (TMDs), phosphorene, silicene, and other 2DMs exhibit low bandgap [8]. TMDs are commonly used in supercapacitors and Li-ion battery electrodes due to their structure, high surface area, and excellent electrochemical characteristics. However, due to their relatively low bandgap, their uses are limited for photodetection in the UV-IR spectrum [8]. Similarly, many other 2DMs show low band gaps, which make them unsuitable for photodetection and optoelectronic applications in the UV or IR regions. So, researchers have been looking for alternate 2DMs since the above-mentioned ones cannot be used in many applications. Among these, layered monochalcogenides based on lead (Pb) and tin (Sn) have drawn attention because of their extraordinary optical, structural, and thermoelectric qualities [9,10]. These compounds, which are made of Sn or Pb mixed with chalcogen elements like tellurium (Te), sulfur (S), or selenium (Se), have properties that make them promising candidates for use in next-generation thermoelectric, optoelectronic, and electrical devices [11].

The van der Waals-type structure of layered monochalcogenides enables them to be exfoliated into thin, two-dimensional sheets. Because of their distinct structure, they have anisotropic qualities that increase their potential for various uses [12]. Xia and Li have reported that these layered monochalcogenides are mainly used in photovoltaics, photodetectors, and energy storage devices because of their capacity to create thin films with tunable electrical and optical properties [13]. Furthermore, Yu et al. reported that these materials are appealing for energy conversion technologies, where effective heat-to-electricity conversion is crucial due to their thermoelectric properties, which are defined by a low thermal conductivity and a high Seebeck coefficient [14]. The past decade has seen tremendous progress in understanding the basic characteristics of Sn and Pb-based monochalcogenides, thanks to developments in computational modeling and experimental methods [15,16]. Moreover, their electronic band topologies, carrier mobility, and defect behavior have been revealed by first-principles simulations, which will help experimentalists refine their characteristics [17,18]. When electrical, optical, and thermoelectric properties are involved, the unique qualities of layered materials frequently outweigh those of non-layered materials [12]. It is possible to control the features of layered materials, such as their increased surface area, improved electrical conductivity, and decreased thermal conductivity, because of the weak van der Waals forces between neighboring layers, which facilitate facile exfoliation into thin 2D sheets. The enhanced control over electron and phonon transport resulting from this structural advantage contributes to increased thermoelectric performance. One of the main advantages of layered materials is their ability to be exfoliated into atomically thin layers, which offers more control over material qualities like optical transparency, thermal conductivity, and electrical conductivity [19]. Due to this tunability, improved performance is possible in areas such as flexible electronics, energy conversion, and optoelectronics [20]. Terada et al. [21] investigated multilayer silicene with a deformed buckling structure and showed that its thermoelectric capabilities can be greatly improved by managing intercalated atoms. By achieving metal-like electrical conductivity and a Seebeck coefficient that was three times more than anticipated, this method significantly increased the power factor of silicene and established it as a potential next-generation 2D Dirac thermoelectric material. Wei et al. [22] demonstrated the promise of layered oxide thin films for next-generation memory devices by proving that they can achieve high polarization values without wake-up cycling and show increased ferroelectric characteristics under epitaxial strain. Similar to this, Yang et al. [23] demonstrated that layered oxychalcogenides produced by self-propagating high-temperature synthesis (SHS) could achieve a state-of-the-art figure of merit value of 1.2, indicating improved thermoelectric performance due to decreased lattice thermal conductivity and increased carrier concentration. Furthermore, the formation of heterostructures with low lattice mismatch is made feasible by the weak van der Waals forces between layers, which opens up the possibility of engineering novel materials with specific qualities for cutting-edge technologies like transistors, photodetectors, and thermoelectric devices. The layered materials covered in this review, including Sn-and Pb-based chalcogenides, are very promising for optoelectronic devices and next-generation energy conversion because of these remarkable properties [24].

Despite these developments, there are still obstacles to Sn and Pb monochalcogenides’ full potential. Significant issues include managing the production of flawless, extensive-area films, comprehending the impacts of alloying and dopants, and enhancing the thermoelectric efficiency at ambient temperature [25,26]. A thorough comprehension of how synthesis conditions impact their composition and characteristics is needed to tackle these obstacles. There has been little effort to review all the work performed on IVA-VIA 2DM compounds, particularly Sn and Pb-based chalcogenides. Most reviews lack in-depth explorations of these compounds. This review aims to comprehensively understand these compounds’ potential in materials science, filling a significant gap in the current research.

This review aims to compile thorough reviews of the recent progress in layered Sn/Pb-VIA compounds, focusing on their structure, synthesis methods, thermoelectric and optical properties, and potential applications as photodetectors and TEGs. In particular, the study emphasizes how Sn/Pb-VIA 2DMs can be used to advance photodetection and energy conversion technologies, especially in NIR and thermoelectric applications. This study also discusses the thermal conductivity, electrical conductivity, and thermoelectric coefficient values of the materials fabricated through different synthesis techniques and doping of other elements. Lastly, Section 6 explores the use of these materials as broadband photodetectors in the infrared (IR) region and their potential in thermoelectric generators, showing that recent advancements position them as strong candidates for next-generation devices.

## 2. Materials and Methods

### 2.1. Synthesis of 2D SnSe

Two approaches—top-down and bottom-up—have been investigated for synthesizing two-dimensional SnSe nanosheets (NSs); each has its benefits, drawbacks, and results. As previously indicated, SnSe has a substantial interlayer binding energy that grows bigger as the layer thickness moves closer to less than 10 nm. Because of this, producing ultrathin SnSe nanoflakes using standard micromechanical exfoliation (ME) is difficult because of the significant interlayer coupling brought on by the presence of Sn lone-pair electrons, which prevents efficient exfoliation. Despite these challenges, ME has been successful in some instances. In contrast to Yang et al. [27], who reported obtaining thicknesses of roughly 7 nm and 28 nm, Cho et al. [28] generated SnSe nanoflakes about 90 nm thick. The manufacture of SnSe nanoflakes was also demonstrated by Yang et al. [29], who initially grew SnSe crystals using the Bridgman method and then exfoliated them using ME onto substrates such as SiO_2_ and quartz. Guo et al. [30] similarly created 50 nm thick SnSe flakes using ME. Other research [31,32] used scotch tape to mechanically exfoliate SnSe flakes from bulk crystals. ME’s main disadvantage is its restricted control over thickness, especially for layers smaller than 10 nm, which limits its widespread scaling even if it is simple to use and produces high-quality nanoflakes. Techniques for liquid-phase exfoliation (LPE), such as LPE with sonication assistance, have shown promise in producing SnSe nanosheets on a large scale. These techniques are beneficial because they may produce large volumes of work at a low cost. To produce SnSe NSs, Huang et al. [33] used sonication in isopropanol for 20 h, adjusting thicknesses with centrifugation rates. They discovered that average thicknesses of 4.3 nm, 8.9 nm, and 5.9 nm were obtained at 12,000 rpm, 3000 rpm, and 8000 rpm, respectively. Their findings demonstrated that selecting the right solvent is essential for maximizing the production of ultrathin nanosheets. N-methyl-2-pyrrolidone (NMP) was also shown to be the best solvent for exfoliating SnSe nanosheets by Ye et al. [34]. They produced nanosheets with thicknesses as low as 2.5 nm by utilizing NMP and different centrifugation speeds. Although LPE techniques are very effective and scalable, they can also induce process flaws and provide very low yields of monolayer materials. In conclusion, there are trade-offs and unique results associated with each synthesis technique for 2D SnSe nanosheets. Mechanical exfoliation is still straightforward but has limitations with controlling thickness, whereas liquid-phase exfoliation can be scaled up but has issues with homogeneity and fault introduction. Although they need more complicated setups and longer processing periods, ion intercalation techniques can produce incredibly thin nanosheets. In order to balance quality, scalability, and ease of manufacturing for 2D SnSe materials, these approaches are being optimized through ongoing research. Equation (1) describes how Li^+^ ions intercalate into SnSe after dissolving in ethylene glycol to generate Li_x_SnSe throughout the process of reduction:

(1) $$SnSe+x{Li}^{+}+{xe}^{-}\to {Li}_{x}^{+}{\left(SnSe\right)}^{-x}$$

Equation (2) indicates that later on when water is introduced to the lithiated SnSe powders, Li^+^ ions found in Li_x_SnSe quickly solvate, producing LiOH and H_2_ gas:

(2) $${Li}_{x}SnSe+xH_{2}O\to SnSe+xLiOH+\frac{x}{2}H_{2}\left(↑\right)$$

**Figure 1:**
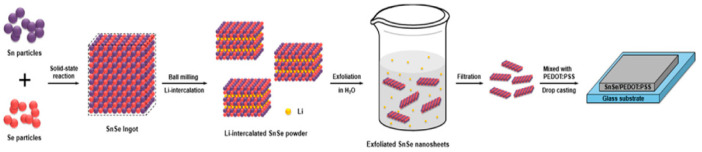
Diagram showing the steps in fabricating the SnSe NS/PEDOT: PSS composite film. Following ball milling, SnSe ingot and Li atoms were intercalated into the SnSe interlayers. SnSe NSs were produced when water was introduced to the lithiated SnSe powders Reproduced from [38] under permissions from copyright clearance center.

The rapid increase in the innermost layers of SnSe allows SnSe’s layers to be exfoliated, successfully preparing SnSe NSs. A comparable method of obtaining SnSe NSs was disclosed by Ren et al. [35] and Qiao et al. [36]. Li^+^ intercalation is used to synthesize SnSe, presented in Figure 1 [37].

Using the LPE sonication-assisted extraction technique and the molecular intercalation approach, Tetrabutylammonium bromide (TBAB) was dissolved in water by Doung et al. [39] to create 2D SnSe NSs, which were then progressively mixed with bulk SnSe powder. Three-layer SnSe NSs with a thickness of 1.82 nm were produced by centrifuging the mixture after it had been sonicated for 24 h in a cold bath to eliminate extra bulk material. They observed that large-lateral-size 2D crystals may be effectively produced by exfoliation using an electrochemical technique. However, this technique is not appropriate for SnSe nanostructures. Although the advantages of the EE approach are its speed, minimal energy consumption, and high efficiency, only a few reports have been made. As a result, many issues and challenges still need to be resolved. The lateral dimensions of the resulting flakes are typically very small, often measuring in the sub-micrometer range. This is due to the use of ultrasonic and Li-intercalation exfoliation in the liquid phase, which effectively overcomes the binding energy of the interlayer and isolates extremely thin SnSe flakes [40,41]. Additionally, using the Li intercalation-assisted liquid exfoliation method yields high-yield SnSe NSs, but many defects are also present [39].

**Figure 2:**
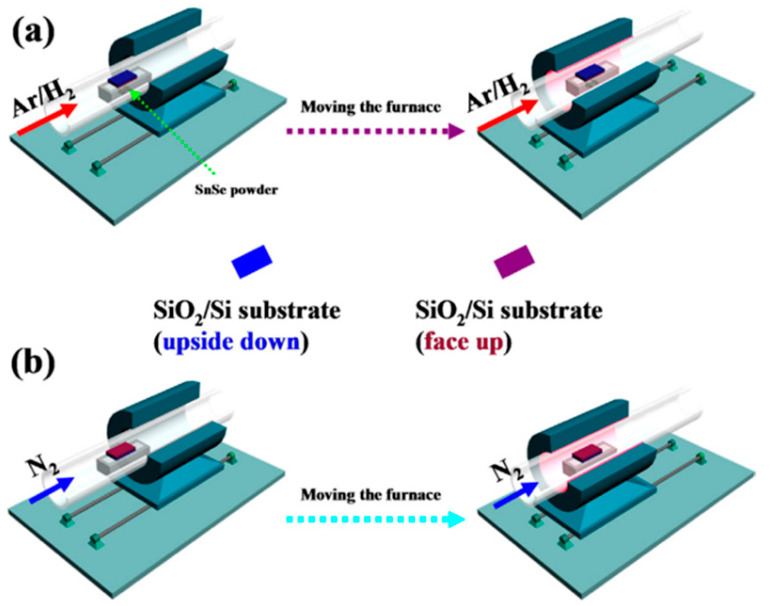
(**a**) Synthesis of bulk SnSe. (**b**) Synthesis of monolayer SnSe using N_2_ etching process [48] under permissions from copyright clearance center.

**Figure 3:**
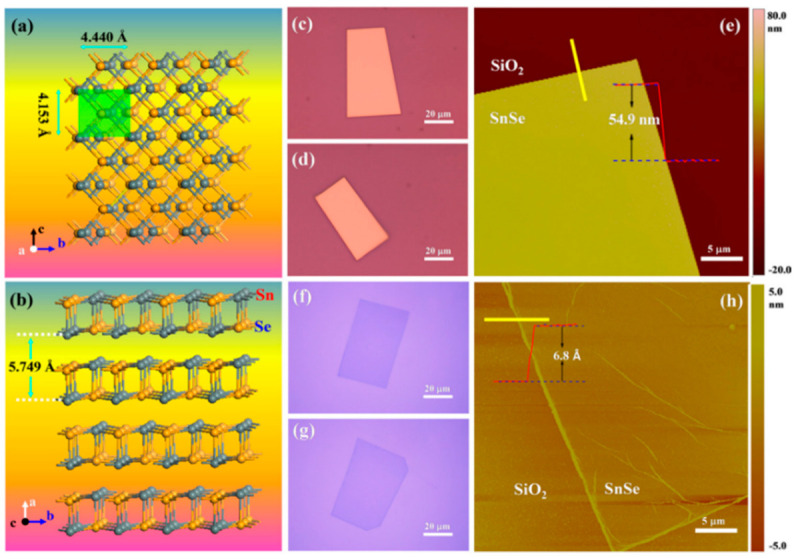
(**a**,**b**) Grey Sn atoms and yellow Se atoms comprise the layered SnSe crystal structures, laterally the *a* and *c* axes. Green shadows represent the single-layer’s primitive cell SnSe; the lattice parameters in the x (armchair) and y (zigzag) directions are 4.440 Å and 4.153 Å. A single layer has a thickness of 5.749 Å. (**c**,**d**) Optical microscope images of bulk SnSe flakes with a rectangular form as they were produced. (**e**) The height profile reveals a thickness of about 54.9 nm, and an average AFM image is captured at the flake edge of (**d**). (**f**,**g**) Optical pictures of single-layer rectangular SnSe flakes as synthesized. (**h**) AFM picture typically shows approximately 6.8 Å of thickness at the flake edge of (**f**) and height profile. Reproduced from [48] under permissions from copyright clearance center.

Some reports have been made on preparing SnSe films that are not 2D layers. The CVD method has only succeeded in SnSe preparation with varying morphologies in terms of 2D SnSe, such as nanoflakes and nanoplates [42,43], nanowires [44,45], and thin films [46,47]. There have been no reports of growing monolayer or 2D SnSe using the CVD method [17], although some authors have mentioned using the CVD method to synthesize a few layers of thick SnSe. Jiang et al. [48] reported the growth of single-layer rectangular SnSe flakes. Figure 2 shows the fabrication processes using the two-step synthesis method. In contrast, Figure 3 shows the film’s crystal structure and optical Atomic Force Microscopy (AFM) images, along with the height profile determined by AFM [48].

In the bottom-up synthesis of 2D SnSe, Physical Vapor Deposition (PVD) is a commonly utilized and efficient method for producing high-quality, stoichiometry-controlled nanoscale SnSe flakes. Nevertheless, PVD-grown SnSe flakes on various substrates usually have some restrictions, like thicker (more than 10 nm) or smaller lateral dimensions (less than 10 µm). Using PVD to produce monolayered SnSe successfully has not yet been reported. Yu et al. [49] combined powdered Sn and Se in a 1:1 ratio and added iodine (I_2_) as a transport agent to encourage crystal formation to build 6 nm thick SnSe nanosheets. The mixture was put in a double-zone furnace after being vacuum-sealed in an ampoule. Nanosheets were effectively produced by gradually cooling the furnace over five days following the development phase. Although it takes a long time to process, this approach offers a regulated environment for the formation of crystals [50]. Hao et al. [51] used magnetron sputtering to create wafer-sized SnSe films for photodetection applications in an alternative method. On SiO_2_/Si substrates heated to 450 °C and vacuumed to 5 × 10⁻⁴ Pa, the SnSe films were deposited. Sputtering was carried out at 10.0 W and 1.0 Pa of argon pressure, and 30 min of vacuum post-deposition annealing enhanced the films’ crystallization. Large-area film production can be achieved using magnetron sputtering, although post-deposition treatments and high vacuum levels are necessary to improve crystallinity. Using recently ground SnSe powder and PVD, Chiu et al. [52] produced huge SnSe flakes on mica surfaces. A temperature gradient and Ar/H_2_ flow helped SnSe evaporate in a furnace’s hot zone during synthesis and deposit on mica downstream. The resulting SnSe flakes were formed in a range of thicknesses, with flakes as thin as 2 nm (3–4 layers) and lateral diameters up to 23 µm. Although this technique successfully expands the side length, maintaining uniform thickness control is still difficult. Jiang et al. [53] used a quartz tube furnace system in a two-step procedure to generate single-layer SnSe flakes on SiO_2_/Si substrates. The resulting SnSe flakes were rectangular and showed a pristine crystalline phase orientated along the *a*-axis. Although this large-scale technique provides good crystalline quality, more work is needed to improve control over the lateral dimensions and thickness of the monolayer [54]. Monolayer SnSe films have also been synthesized via Molecular Beam Epitaxy (MBE). Zhou et al. [55] grew SnSe films on mica substrates using tin pellets and refined SnSe particles as evaporation sources. To control Sn vacancies and the electrical properties of the films, the Sn source temperature was adjusted between 700 °C and 800 °C while the substrate temperature was kept constant at 250 °C. The monolayer SnSe films had an average thickness of 0.68 nm. While MBE yields excellent monolayers with fine control over thickness, the technique is costly and necessitates high vacuum levels. Chang et al. [56] improved upon the MBE method by fabricating single-layer SnSe plates on graphene substrates using a van der Waals epitaxy approach in an ultra-high vacuum environment. The substrate temperatures were adjusted between 70 °C and 220 °C to produce a variety of plate morphologies—increases in substrate temperature produced thicker, straight-edged plates with surface steps around 6 Å high. First, an irregularly shaped monolayer was deposited at a lower substrate temperature in the two-step growth procedure, and then the monolayer was annealed to produce well-defined rectangular plates. While this approach allows for fine control over thickness and shape, it takes several steps to reach the ideal morphology. A different synthesis technique called microwave-aided solvothermal synthesis was presented by Hartl et al. [57]. Their investigation produced stable SnSe precursors by demonstrating in situ reductive bond cleavage in a dimeric diselenide ligand (SeC_2_H₄N(Me)C_2_H₄Se)_2_. They created SnSe_2_ particles with a CEM Discover 2.0 device by dispersing 40 mg of SnSe_2_ in dehydrated NMP and heating it in a microwave. This method allows for quick synthesis and scalability but needs exact control over the microwave parameters to prevent unintended side effects. 

In conclusion, there are specific benefits and trade-offs associated with film thickness, lateral size, and scalability for each of these processes: PVD, chemical vapor transport, magnetron sputtering, MBE, and microwave-aided solvothermal synthesis. However, expensive and complicated techniques such as MBE offer fine control over thickness and quality. Monolayer SnSe film production presents difficulties for more scalable techniques like PVD and LPE. These techniques need to be optimized further to produce 2D SnSe materials on a wide scale and with good quality. Table 1 summarizes the synthesis of 2D SnSe layers prepared by various methods and thicknesses.

### 2.2. Synthesis of 2D SnS

**Figure 4:**
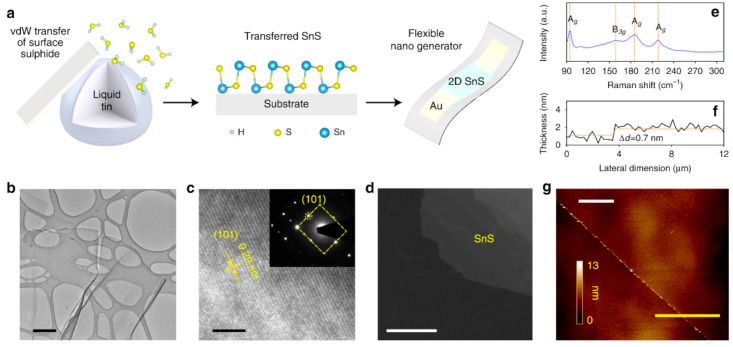
(**a**) Synthesis process of monolayer SnS, including its applications and thickness. (**b**) TEM image. (**c**) HRTEM fringe pattern demonstrating a 0.29 nm d-spacing matched to the (101) plane. (**d**) Two-dimensional SnS dark field TEM picture demonstrating a reduced appearance of grain borders verifying single crystal SnS. (**e**) Raman spectroscopy. (**f**) Thickness profile of 0.7 nm corresponding to a single atom thick SnS. (**g**) AFM picture of single layer thick SnS layer. Reproduced from [62] under permissions from copyright clearance center.

Two-dimensional SnS NSs have been created using a variety of processes, such as solvothermal, LPE, CVD, and PVD. Each technique provides unique results regarding nanosheet quality, thickness, lateral dimensions, and overall scalability. Every strategy, though, has a unique set of benefits and drawbacks. SnS NSs have been produced widely via PVD for various applications. To create SnS NSs for photodetection, Zhang et al. [60], for instance, placed a mica substrate downstream in a tube furnace heated to 720 °C. Nanosheets with respectable crystallinity and acceptable lateral dimensions were produced using this technique. PVD techniques, however, generally have difficulty producing homogeneous thicknesses and monolayers, which restricts their capacity to produce ultrathin nanosheets with a significant surface area. Yuan et al.’s [67] version of this technique yielded SnS NSs with a thickness of 12 nm. The PVD technique’s failure to attain monolayer thickness persists despite the films’ suitability for specific applications such as photodetection. Because of its relative ease of use and scalability, LPE has become popular. Bilayer SnS nanosheets were successfully manufactured via LPE by Brent et al. [61]. The process involved sonicating bulk SnS in NMP and then centrifuging the dispersion to separate the nanosheets. The device’s performance may be impacted by this method’s inability to manage lateral size and thickness homogeneity despite its effectiveness in manufacturing thin flakes. Similarly, Sarkar and Stratakis [72] produced SnS NSs using LPE in several solvents, yielding nanosheets that were 10 nm thick in IPA but only 4.5 nm thick in NMP. LPE is perfect for large-scale manufacturing since it is simple and inexpensive, but it frequently produces nanosheets with flaws and no control over thickness. Another method that shows promise for producing SnS NSs rapidly is Microwave-Assisted Synthesis. SnS NSs were created by Modi et al. [65] by microwave-irradiating a solution of SnCl_2_ and thioacetamide (TAA) with PVP for 30 min. Centrifugation was used to purify the black SnS precipitates obtained using this approach. Although microwave irradiation is a quick and energy-efficient technique, uneven heating and response control can lead to variability in the size and shape of nanosheets. SnS NSs can also be synthesized via solvothermal methods, as shown by Li et al. [73], who utilized a solvothermal strategy to produce SnS NSs with reduced graphene oxide (RGO). This approach produced 10 nm thick nanosheets by autoclaving the reaction mixture for 18 h at 190 °C. While solvothermal methods can yield high-quality NSs with well-defined thickness, their scalability, and speed are limited in comparison to other methods because of their lengthy reaction periods and requirement for high-temperature autoclaving. The process of CVD has been used to produce SnS nanoflakes with precise lateral and thickness dimensions. For instance, Liu et al. [71] created SnS nanoflakes with thicknesses ranging from 25 to 95 nm by synthesizing SnO and sulfur precursors in a quartz tube heated to 650 °C. Although CVD gives exact control over flake thickness and dimensions, it usually necessitates high temperatures and specialized equipment, adding to the process’ complexity and expense. An additional top-down method for producing monolayer or few-layer SnS NSs is van der Waals exfoliation. Using this technique, Khan et al. [62] successfully manufactured monolayer SnS. Van der Waals forces are used in this approach to exfoliate tiny layers from bulk materials. Although this technique can provide monolayers of excellent quality, its poor yield and scalability frequently make it impractical for use in commercial settings. Figure 4 shows the synthesis process, high-resolution TEM (HRTEM) image, tunneling electron microscope (TEM) image, Raman spectrum, thickness profile, and surface image using AFM for monolayer SnS NSs [63].

**Figure 5:**
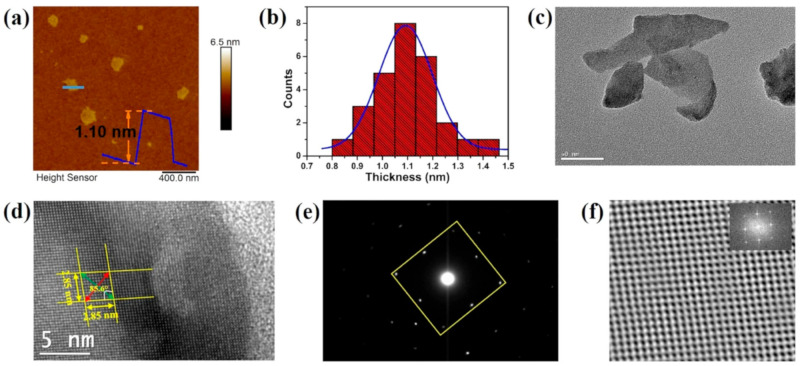
SnS NSs (**a**) AFM image with height profile. Scale bar length is 400 nm. (**b**) Thickness distribution histograms. (**c**) TEM pictures. (**d**) Ultrathin SnS HRTEM picture. (**e**) SAED pattern corresponding to (**d**) image. (**f**) The chosen area in (**d**)’s atomic resolution is filtered by the fast Fourier transform (FFT) (Inset: The chosen region’s FFT pattern in (**d**)). Reproduced from [42] under permissions from copyright clearance center.

Jamali-Sheini et al. [64] employed thermal evaporation to produce SnS nanosheets by depositing SnS onto SiO_2_ substrates in a tube furnace under regulated conditions. Although thermal evaporation frequently yields thicker, less controllable nanosheet production compared to other approaches, this method created nanoscale SnS particles. However, it is a useful method for making more nanosheets in greater quantities while maintaining reasonable control over particle size. In conclusion, all synthesis methods have limitations despite their unique benefits. High-quality nanosheets can be produced with PVD and CVD, but their capacity to create monolayers and expensive equipment are usually their limitations. Although LPE offers great cost-effectiveness and scalability, some applications may struggle with the lack of control over flaws and sheet thickness. While solvothermal and microwave-assisted techniques provide quick synthesis, they need to be optimized for better scalability and consistency. Although it has a low yield, van der Waals exfoliation is a potent technique for producing monolayers. The final decision about the synthesis technique is made in light of the intended SnS nanosheet characteristics and the intended use. Sarker et al. [42] used the LPE approach to process tin (II) sulfide to produce ultrathin SnS NSs. The solution was centrifuged for 20 h under sonication to separate the nanosheets, and the result was monolayered SnS NSs with an average thickness of 1.10 nm. The characteristics of these sheets were examined in more detail, as shown in Figure 5. In another report, Khan et al. [63] mentioned that they fabricated SnS NSs. They degassed and eliminated any remaining water by heating. They introduced instantaneous change in the solution, making the solution hue from pale yellow to black-brown, which signifies the creation of nanoparticles. During the process, aliquots were taken at various intervals. Table 1 summarizes the synthesis of 2D SnS layers prepared by various methods and thicknesses.

### 2.3. Synthesis of 2D SnTe

**Figure 6:**
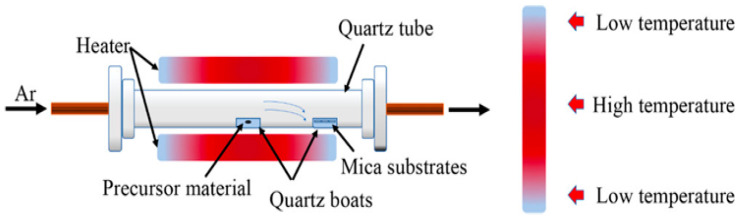
The growth process of 2D SnTe nanosheet platelets (NPLs) created using CVD. Reproduced from [83] under permissions from copyright clearance center.

**Figure 7:**
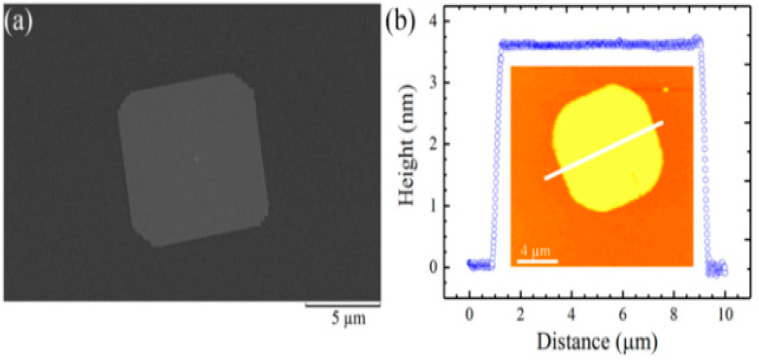
(**a**) SEM micrograph and (**b**) AFM picture of NPLs with height profile. Reproduced from [83] under permissions from copyright clearance center.

Most of the work performed so far for the investigations of 2D SnTe layers is related to theoretical calculations. Many authors reported the structural and other properties of 2D SnTe layers based on DFT calculations. However, a few reports have focused on synthesizing 2D SnTe layers using different methods. These methods are mostly bottom-up types. There has been no report on top-down approaches to preparing the 2D SnTe layers. According to Chang et al. [26], exfoliating SnTe flakes from bulk materials is typically not feasible due to the material’s three-dimensional rock salt structure at ambient temperature. Li et al. [81] used hot injection and colloidal synthesis in a nonpolar solvent to create quasi-2D SnTe nanocrystals. The nanostripes had a rock salt crystal structure and were regulated in thickness using trioctylphosphine, oleic acid, and 1-bromotetradecane (1-BTD). Once the solution’s color changed to signal that the reaction was complete, the SnTe nanosheets (NSs) were purified using several centrifugation cycles. Using diphenyl ether, oleic acid, and tri-n-octylphosphine under nitrogen, He et al. [82] created 2D SnTe nanosheets. After heating and degassing the mixture, a tri-n-octylphosphine-tellurium precursor was added, and the reaction took place at 210 °C. The process was quenched by removing the heating mantle, and the resultant nanostructures were purified. Liu et al.’s study [83] showed how the CVD method allowed the controlled van der Waals development of 2D SnTe nanoplates on mica substrates as shown in Figure 6. They demonstrated how the mica surface aided in the creation of six monolayers of very thin SnTe nanoplates. They successfully regulated the shape of the nanoplates because of the surface energy differences between the {111} and {100} planes by varying the growing temperature. The SEM and AFM images are depicted along with the height profiles of 2D SnTe nanoplates in Figure 7a and Figure 7b, respectively [83]. 

**Figure 8:**
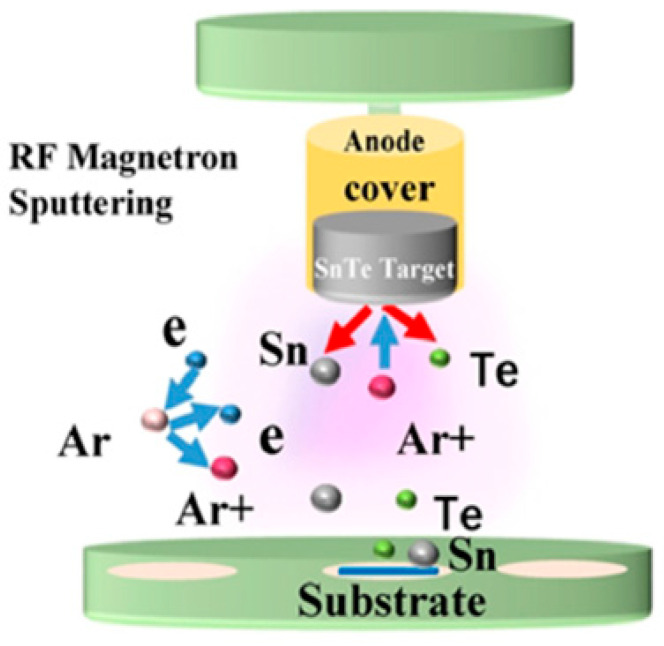
Schematic diagram depicting the deposition of SnTe nanofilm through the magnetron sputtering method. Reproduced from [84] under permissions from copyright clearance center.

Large, thin SnTe nanosheets (NSs) were created by Song et al. [84] through the hot-injection technique, in which a TOP-Te precursor was injected after a mixture of oleic acid, Sn(CH_3_CO_2_)_2_, diphenyl ether, and TOP was heated to 250 °C. Centrifugation was used to purify the SnTe NSs after the solution changed color, indicating nanostructure production. Additionally, they used magnetron sputtering to create SnTe nanofilms by varying the sputtering power (40–80 W) and deposition duration while maintaining a steady chamber pressure of 5 Pa without heating the substrate. The schematic diagram illustrating the magnetron sputtering method’s deposition of SnTe nanofilm is presented in Figure 8 [84].

On graphitized 6H-SiC (0001) substrates, Chang et al. [85] showed the development of monolayer SnTe nanosheets (NSs) up to 1 µm. Before SnTe was deposited using MBE at substrate temperatures ranging from room to 200 °C, the SiC substrates underwent annealing and graphitization. They discovered that larger-area films formed at 130–180 °C, whereas lower substrate temperatures resulted in fractal development with uneven edges, and higher temperatures generated islands with straight edges. Table 1 summarizes the synthesis of 2D SnTe layers prepared by various methods and thicknesses.

### 2.4. Synthesis of 2D PbTe

Most of the work related to the investigations of 2D PbTe layers is related to theory. Many authors reported the structural and other properties of 2D PbTe layers based on density function theory (DFT). However, a few reports have been on synthesizing 2D PbTe layers using different methods. These methods are mostly bottom-up types. There has been no report on top-down approaches to preparing the 2D PbTe layers. 

**Figure 9:**
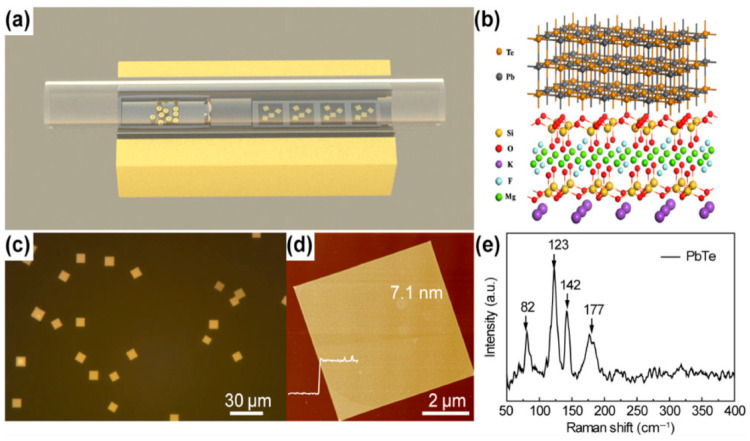
(**a**) Diagram showing the growth setup for 2D PbTe nanosheet van der Waals epitaxy. (**b**) A side view of the 2D PbTe crystal structure, (**c**) Two-dimensional quadrilateral PbTe NSs grown on mica viewed optically. (**d**) An AFM picture of a 7.1 nm-thick PbTe nanosheet. (**e**) The synthetic PbTe NSs’ Raman spectra. Reproduced from [86] under permissions from copyright clearance center.

Using the PVD method, Zhao et al. [86] created PbTe nanosheets (NSs) with a thickness of roughly 7.1 nm on fluorophlogopite mica substrates. The NSs were square-shaped. High-purity Ar gas was pumped through the system before the growth, and the furnace was heated to 600–750 °C for 0.5–10 min. Following the growth process, the furnace was allowed to cool gradually while using H_2_ gas as the carrier at a flow rate of 150 SCCM. PbTe nanosheet generation [86] is shown in Figure 9, along with their synthesis, crystal structure, optical image, thickness, and Raman analysis. Using a hydrothermal process, Zhu et al. [87] combined lead nitrate, sodium hydroxide, PVP, and Na_2_TeO_3_ in distilled water before adding hydrazine hydrate to create PbTe nanosheets (NSs). After five minutes of stirring, the liquid was put in a stainless-steel autoclave lined with Teflon and heated to 160 °C for a full day. After the reaction, the autoclave was left to cool to room temperature, producing the PbTe NSs spontaneously. Table 1 summarizes the synthesis of 2D PbTe layers prepared by various methods and thicknesses.

### 2.5. Synthesis of 2D PbSe

**Figure 10:**
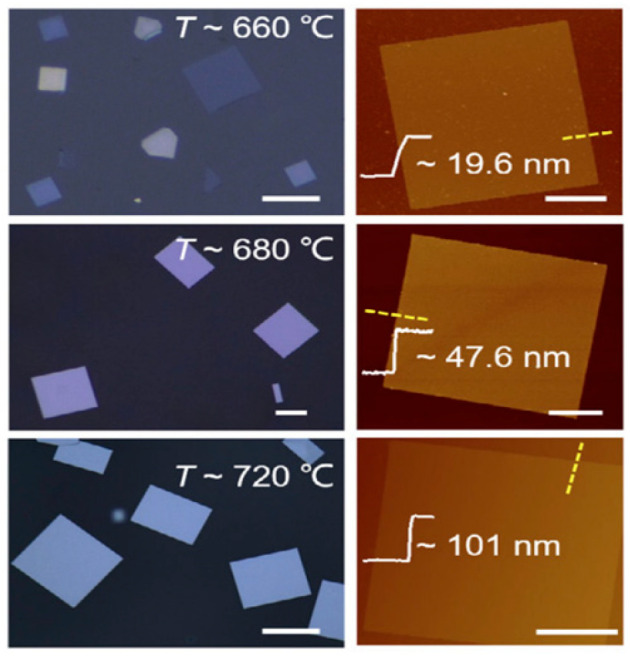
Optical (**left column**) and AFM images (**right column**) along with height profiles of as-grown PbSe NSs on mica substrate. Reproduced from [88] under permissions from copyright clearance center.

Using a salt-assisted APCVD method in a horizontal quartz tube furnace, Jiang et al. [88] created PbSe nanosheets (NSs) by mixing lead selenide and NaCl particles and adding Se powder upstream. After flushing the tube with Ar gas, the downstream center was heated to 680 °C and the upstream center to 400 °C. Throughout the 10-min holding period, a constant flow of 20% H_2_/Ar gas was maintained. PbSe NSs were effectively produced on fluorophlogopite mica substrates following natural cooling. The optical microscope, AFM, and matching height profiles of the as-grown PbSe NSs are displayed in Figure 10 [88]. PbCl_2_, oleylamine, octylamine, and Aliquat 336 were heated in an inert atmosphere by Koh et al. [89] to create PbSe nanoplates, which were then injected with Se powder. Ethanol precipitated the product, which was then repeatedly centrifuged and redispersed to remove impurities. They substituted PbI_2_ for PbCl_2_ to create thicker nanoplates, and they saw faster development and an increase in thickness throughout the reaction. Using a Schlenk device in an inert gas environment, Klepig et al. [90] created 2D PbSe nanocrystal platelets with a cubic rock salt structure at low temperatures (0 °C and ambient). The reaction mixture rapidly changed from colorless to dark brown following the injection of the Se precursor into the Pb-precursor solution. After ethanol precipitation and centrifugation, the product was re-dispersed in hexane for storage.

### 2.6. Synthesis of 2D PbS

**Figure 11:**
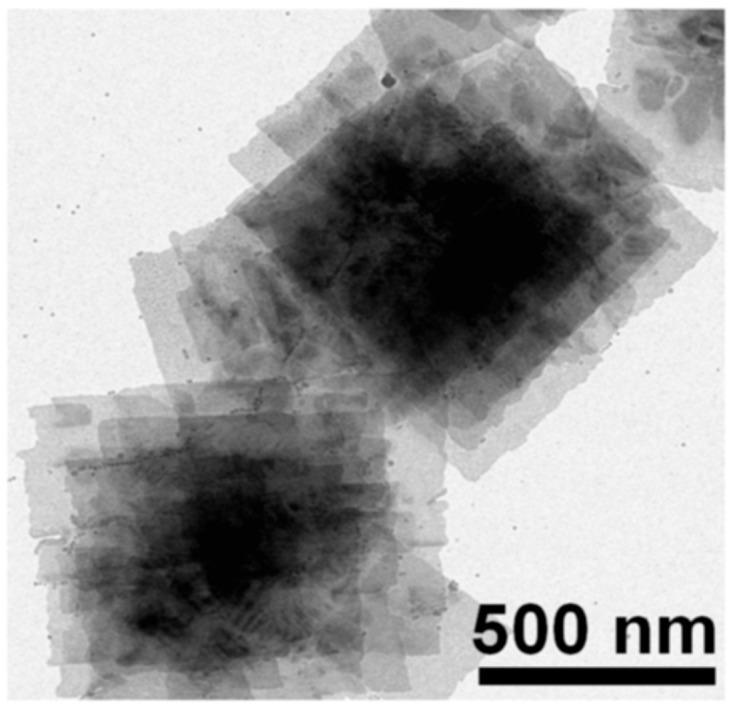
TEM micrograph of stacked PbS NSs prepared by 2D oriented attachment. Reproduced from [97] under permissions from copyright clearance center.

Colloidal PbS nanosheets (NSs) have been synthesized using various approaches, each yielding distinct results about the nanosheet thickness, lateral dimensions, and scalability. However, every strategy has advantages and disadvantages of its own. PbS NSs with precisely defined thickness and lateral dimensions have been produced via the widespread application of colloidal synthesis. Using a colloidal technique, Bhandari et al. [91] combined lead acetate trihydrate with diphenyl ether, oleic acid, and nitrogen atmosphere. The resultant PbS NSs were distributed in toluene and ranged in thickness from 1.2 to 4.6 nm. Although accurate control over lateral dimensions can be difficult to achieve, this technology allows for good control over nanosheet thickness and size. Additionally, the process is time-consuming. Post-synthesis processing procedures like centrifugation and solvent washes are often necessary for colloidal synthesis, adding complexity and perhaps increasing product variability. Gao et al. [92] used liquid-phase exfoliation to create PbS nanoplatelets by dispersing PbS powder in degassed NMP and sonicating it for a whole day. Long-term ultrasonic treatment produced nanosheets, which were then centrifuged to remove bigger particles. LPE has the benefit of being somewhat economical and scalable, although it frequently produces nanosheets with less control over lateral dimensions and wider thickness distributions. In addition, structural flaws in the nanosheets may result from the lengthy sonication durations needed. As shown by Aerts et al. [94], who dissolved lead acetate in diphenyl ether with trioctylphosphine and oleic acid in a nitrogen atmosphere, directed attachment provides an additional method for producing PbS NSs. The PbS NS thickness (4–7 nm) may be precisely controlled with the addition of trichloroethane. For the purpose of creating NSs with precise thickness and excellent uniformity, directed attachment is useful. Unfortunately, this approach frequently necessitates close observation of the reaction conditions and the use of specific reactants, which raises the process’s complexity and expense. PbS NSs have been grown on substrates on a massive scale using Chemical Vapor Deposition (CVD). PbS nanoplates on SiO_2_/Si substrates were created by Wen et al. [96] utilizing a CVD method that involved heating PbS powder to 500–750 °C while H_2_ gas was present. PbS NSs with thicknesses between 5 and 35 nm were created using this technique. When it comes to producing huge quantities of nanosheets, CVD is very efficient and provides excellent quality control. Its scalability is limited, and production costs rise, as it usually demands high temperatures and specialized equipment. Schleicher et al. [97] also employed 2D-Oriented Attachment to create PbS NSs with a thickness of 2.2 nm and lateral dimensions exceeding 1000 nm. This process included dissolving lead acetate in diphenyl ether, vacuum degassing, and reacting with co-solvents that contained chlorine and a sulfur precursor. Large lateral dimension nanosheets can be produced with the 2D-oriented attachment approach, although controlling the thickness homogeneity of the nanosheets can be challenging. The procedure could also be made more difficult by using particular co-solvents and post-synthesis purification procedures. Zhu et al. [95] used microwave-assisted synthesis to create lead nitrate, potassium thiocyanate, and polyethyleneimine in formamide that was heated to 80 °C in order to create PbS NSs for gas-sensing applications. This process yields nanosheets with good gas-sensing qualities and allows for quick production. However, compared to other techniques, regulating thickness and lateral dimensions is more challenging when using microwave irradiation, and it may lead to uneven nanosheet morphologies. Figure 11 shows the TEM image stacked PbS NSs using TCE [97].

**Figure 12:**
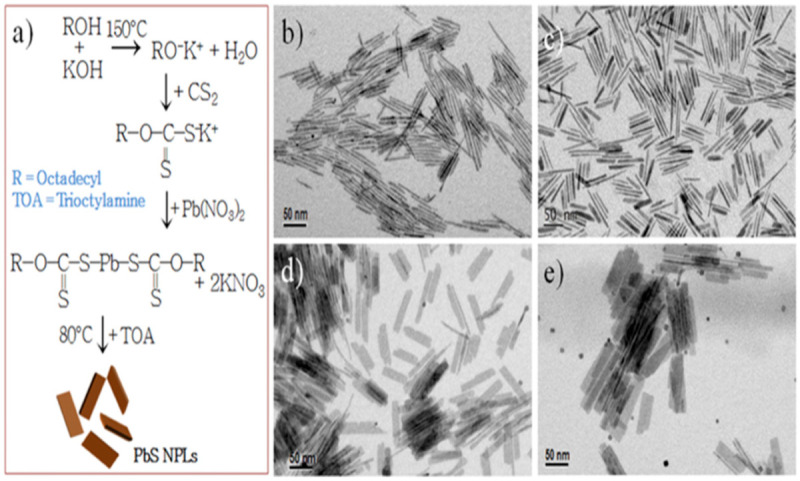
(**a**) Synthesis of PbS NPLs using the colloidal approach. TEM pictures of PbS NPLs that were created by annealing the material for (**b**) 10 min, (**c**) 40 min, (**d**) 2 h, and (**e**) 5 h at 80 °C. Reproduced from [102] under permissions from copyright clearance center.

The ability to precisely control thickness and lateral dimension makes Colloidal Synthesis a preferred method for manufacturing PbS nanosheets. Utilizing a combination of trioctylphosphine (TOP) and dimethylformamide (DMF), Zhang et al. [98] reacted lead acetate with bis(trimethylsilyl)sulfide (TMS) to create PbS NSs. Following 1200 s, PbS NSs with lateral dimensions up to 1 µm and an average thickness of 2.0 nm were produced by the process. Although centrifugation and solvent washes are two common complicated purification processes in colloidal synthesis, they can increase the total duration and complexity of the process despite their effectiveness in producing thin and homogeneous nanosheets. Another method is cation exchange, as Wu et al. [99] showed when they created PbS nanoplates (NPLs) by exchanging ions with Cu_2_S nanoplates. PbS NPLs with an average thickness of 3.5 nm and lateral diameters of 120–150 nm were produced by heating the lead oleate solution and vigorously swirling it for two weeks. Although the procedure is sluggish and needs longer reaction periods to produce the appropriate results, cation exchange is useful for creating nanosheets with regulated thicknesses. Acharya et al. [100] used nanowire decomposition to break down lead hexadecylxanthate in trioctylamine, resulting in PbS nanosheets. This procedure produced rectangular nanosheets that were 1.8 nm thick and had lengths ranging from 3 to 20 µm. The Langmuir–Blodgett (LB) technique was then used to deposit these nanosheets onto surfaces. Large nanosheets can be produced using this method, although the process can become more complex due to the need for surface deposition techniques and nanowire precursors. Vasquez et al. [101] used thiourea-based synthesis, producing Pb(OA)_2_ as the lead precursor and the sulfur precursor using thiourea and noctylamine. PbS nanoplates were created after 16–18 min of reaction at comparatively low temperatures (32–38 °C), which were then centrifuged and treated with CdCl_2_. Although the synthesis at ambient temperature is very fast and scalable, high-purity nanosheets require post-synthesis modification. Khan et al. [102] employed octadecylxanthate-based synthesis, dissolving potassium octadecylxanthate in water under argon flow to generate the lead octadecylxanthate (PbOctdX) precursor. After 20 h of processing at 80 °C, PbS nanoplates with a thickness of about 1.8 nm were produced. Although this process effectively creates thin nanosheets, it is made more difficult by the longer reaction time and requirement for inert gas conditions. The production mechanism of PbS NPLs is illustrated in Figure 12 with TEM micrographs annealed for different durations [102].

áPeter Lu [103] created a lead precursor by dissolving lead acetate trihydrate in diphenyl ether (DPE) and oleic acid (OA), which allowed them to build 2.2 nm thick PbS nanosheets. At 130 °C, the lead precursor was mixed with the sulfur precursor (produced from TAA), and the reaction was periodically stopped with cold toluene. Centrifugation was used to gather the products, which were then diluted in methanol. Premathilaka et al. [104] dissolved lead oxide in oleic acid and DPE to create PbS NSs with adjustable lateral diameters ranging from 100 to 1500 nm. Following the degassing process, the sulfur precursor was added at 130 °C, and 1,1,2-trichloroethane was added at a temperature slightly below its boiling point. After being chilled and diluted with toluene, the product was centrifuged to collect it. PbS nanoplates (NPLs) were created from CuS NPLs by Sonntang et al. [105] using cation exchange. Lead oleate was created by heating lead oxide and oleic acid together. After that, lead oleate was added to a CuS NPL dispersion and agitated for 20 min. Centrifugation was used to purify the goods, and toluene was used to wash them. By dissolving lead acetate trihydrate in DPE and OA, degassing the mixture, and adding coligands based on alkylamines, Bielewicz et al. [106] created 10 nm thick PbS NSs. TAA dissolved in DMF was added to start the reaction, and it was aggressively agitated until it cooled. Centrifugation was used to gather the product, and toluene was used for washing. Lead acetate trihydrate in DMF was combined with CS_2_ by Han et al. [107] to create single-crystalline PbS NPLs, which are black PbS products. After ethanol and water washing, the goods were allowed to air dry. The sheets measured 50 nanometers thick. Using a one-pot synthesis, Shkir et al. [108] created PbS NSs by dissolving lead acetate in distilled water, sodium dodecyl sulfate, and NaOH at 60 °C. A second thiourea solution was added to start the reaction and render the solution black, which signifies the creation of PbS. The products, which had less than 5 nm thicknesses, were dried in a furnace at 80 °C. To create 1.2 nm thick PbS nanosheets, Akkerman et al. [109] dissolved Pb in octadecene, OA, and oleylamine and heated the mixture to 165 °C. This was performed in a three-neck flask. At 150 °C, PbS NSs started to form; the solution was then rapidly cooled using a water bath. Centrifugation was used to gather the nanosheets, which were then disseminated again in toluene. Using the CVD technique, Gu et al. [110] produced PbS NSs for photodetecting applications by positioning sulfur and PbCl2 at different points within a quartz tube. After the system was cleared out, heated, and backfilled with Ar gas, PbS nanoplates developed on mica substrates. The resultant nanoplates were 5 µm in lateral size and averaged 50 nm thick. Table 1 summarizes the synthesis of 2D PbS layers prepared by various methods and thicknesses.

An overview of the several manufacturing processes, including synthesis, deposition, and exfoliation techniques, for 2D nanosheets (NSs) and 2D Sn/Pb-VIA materials is given in this section. Depending on the required material qualities, scalability, and application, each process has pros and cons. For high-quality, large-area 2DMs, CVD and MBE are commonly employed; however, solution-based techniques provide a more economical and scalable alternative, albeit with certain structural control trade-offs. Both chemical and mechanical exfoliation methods are still helpful for creating high-purity monolayers, but their application is usually restricted to small-scale manufacturing. Overall, the choice of fabrication process is determined by the particular needs for thickness control, material quality, and the intended use. Research is now being conducted to optimize these techniques for improved accuracy and efficiency in fabricating 2DMs.

## 3. Structural Properties

### 3.1. SnSe Structure

**Figure 13:**
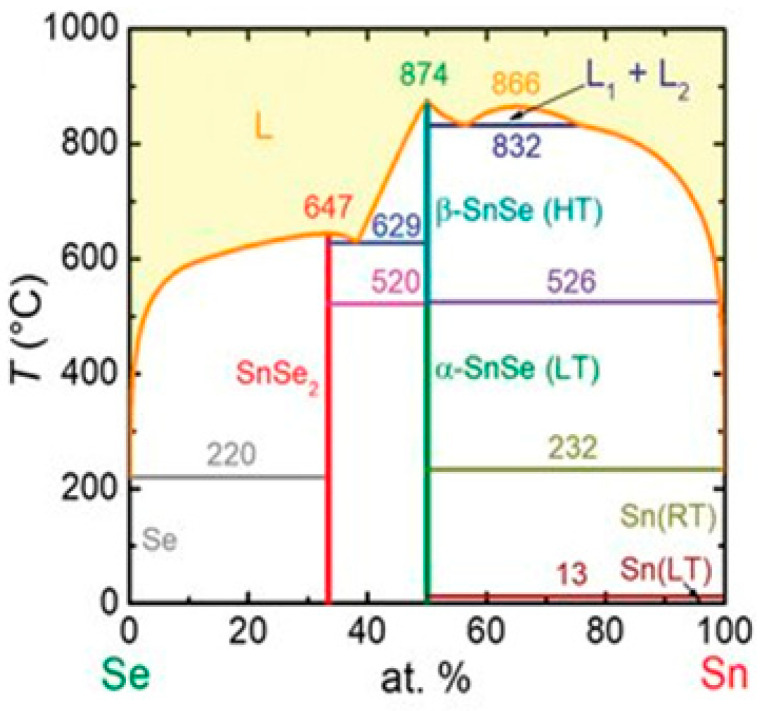
Phase diagram of SnSe. Reproduced from [111] under permissions from copyright clearance center.

The Sn–Se phase diagram, depicted in Figure 13, clearly shows that SnSe primarily has two stable phases. The material α-SnSe exhibits a low-temperature orthorhombic phase at temperatures below 800 K, while the material β-SnSe adopts a high-temperature orthorhombic phase at temperatures above 800 K. Pallikara et al. mentioned that SnSe can also exist in the cubic *Fm*$\bar{3}$*m* (rock salt), *P*2_1_3 (π-cubic) and *F*4$\bar{3}$*m* (zincblende) structures [111]. 

**Figure 14:**
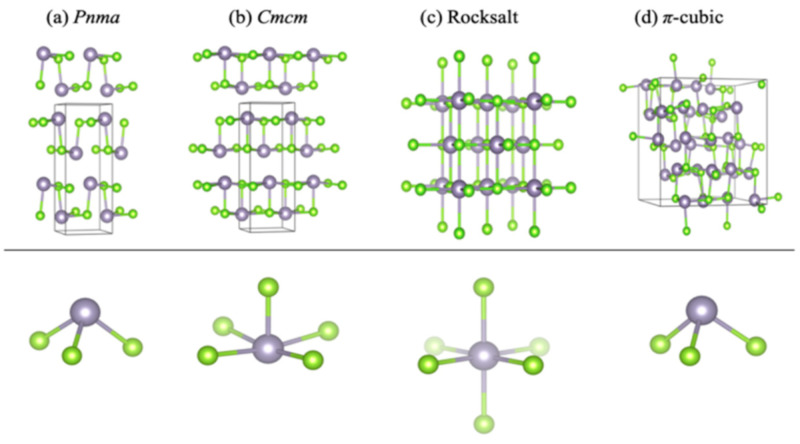
Crystal structure of various phases of SnSe. The green ball is Se and the purple ball is Sn. Reproduced from [111] under permissions from copyright clearance center.

From the Sn–Se binary phase diagram, we can observe that SnSe exhibits a high melting point of 1134 K. According to Shi et al., the α-SnSe phase performs significantly more steadily than the β-SnSe phase [112]. Above 750 K, α-SnSe exhibits a second-order phase transition of the displacive type, transitioning from α-SnSe to β-SnSe. While the lattice constants of these two phases differ, their crystal structures are comparable [111,113]. α–SnSe is an orthogonal structure part of the Pnma space group [114]. A unit cell of α–SnSe comprises eight atoms. The crystalline structure of SnSe is made up of layers with two planes of interconnected Sn and Se atoms. These atoms are linked by heteropolar bonds, creating zigzag patterns within the layers. Three adjacent Se atoms link with each Sn atom, and three adjacent Sn atoms bond with each Se atom. Van der Waals forces and long-range electrostatic attractions primarily bind the neighboring layers of both α-SnSe and β-SnSe [115,116]. The crystal structures of α-SnSe, β-SnSe, and other phases, including zinc blend, π-SnSe, and rock salt structures, are depicted in Figure 14 [111]. 

Despite belonging to the Cnma space group, β-SnSe also possesses an orthogonal structure with lattice parameters a = 4.3062 Å, b = 11.7243 Å, and c = 4.3178 Å [117]. Figure 14 illustrates that β-SnSe, like α-SnSe, possesses a layered structure with strong heteropolar bonding, an anisotropic band structure, high crystal symmetry, and TE characteristics [111,118]. The geometric structure of π-SnSe is cubic, and its lattice parameters are a = 11.9702 Å. Its unit cell has 64 atoms in it. Of them, 32 are atoms of Sn, and 32 are atoms of Se [119]. This cubic phase, which was recently reported to be a metastable phase of both materials, is a member of the non-centrosymmetric crystal class. The π-SnSe structure can be understood as a pseudo-2D layered structure created by the alternating layers of the rock salt SnSe structure being mismatched along one direction. In this manner, the lead (II) cations adopt a deformed local geometry. Lead (II) forms three bonds with adjacent chalcogen ions within each layer. Sn 5 s lone pair with a stereochemical activity that extends into the interlayer gap to promote a dispersive interaction between layers. In a rock salt SnSe structure, there is an octahedral bonding, and each lead (II) cation has six nearest-neighbor chalcogen ions. Rock salt has a cubic geometric structure with lattice parameters of a = 5.71 Å [120]. Although theoretically mentioned, there has not been much work to explore the properties of the zincblende SnSe phase, probably because of its instability [111].

**Figure 15:**
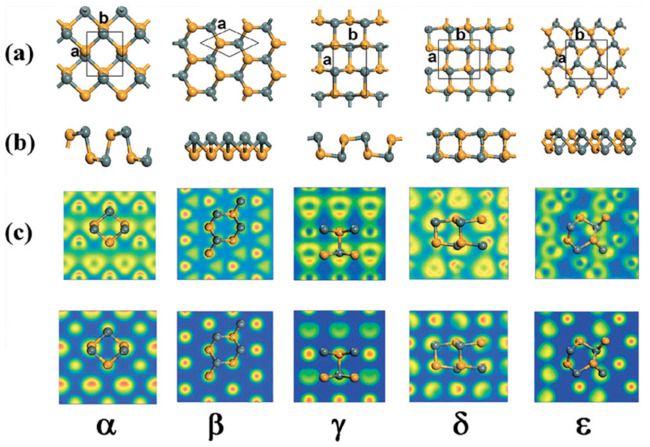
(**a**) Top and (**b**) side views of the α-SnSe, β-SnSe, γ-SnSe, δ-SnSe, and ε-SnSe monolayer geometric structures. The lattice parameters are a and b. Gray balls represent Sn atoms, and yellow balls represent Se atoms. Wigner–Seitz cells are shown in black lines. (**c**) The top views display simulated scanning tunneling microscopy images (+2 V and −2 V) of α-SnSe, β-SnSe, γ-SnSe, δ-SnSe, and ε-SnSe. Reproduced from [122] under permissions from copyright clearance center.

Additional allotropes of monolayer SnSe, including γ-SnSe, δ-SnSe, and ε-SnSe monolayers, have been anticipated [121]. Hu et al. [122] used ab initio DFT to describe the monolayer SnSe’s electrical structure. They stated that there are five different allotropes of monolayer SnSe, including the α, β, γ, δ, and ε phases. According to their report, Figure 15 depicts the five common honeycomb polymorph structures of SnSe, which are α-SnSe, β-SnSe, γ-SnSe, δ-SnSe, and ε-SnSe. According to their findings, these five polymorphs have monolayer morphologies that match a single layer of bulk SnSe (buckled β phase), suggesting that they may be stable. They reported that only β-SnSe has an isotropic structure, while the four other phases, α-SnSe, γ-SnSe, δ-SnSe, and ε-SnSe, are anisotropic. Table 2 shows the lattice groups, lattice parameters, binding energy, and bandgap energy of five allotropes of monolayer SnSe.

**Figure 16:**
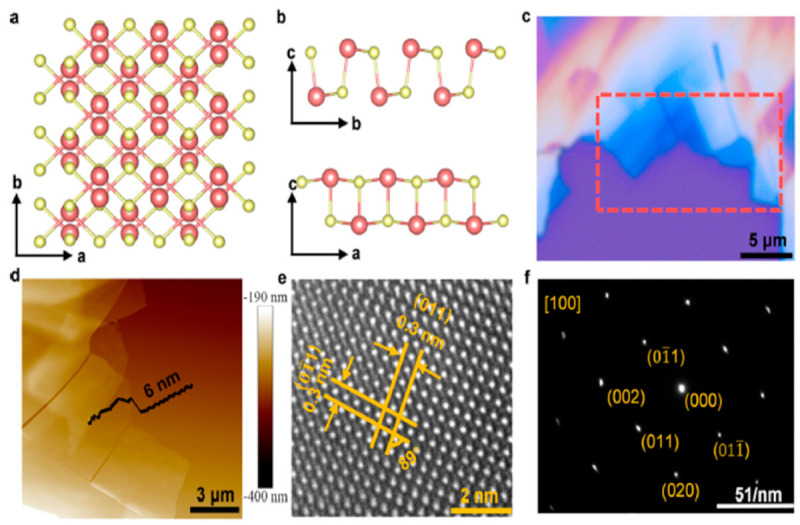
(**a**) SnSe nanosheet crystal structure diagram along the *a* and *b* axes; the pink and yellow balls, respectively, represent the Sn and Se atoms (top view) (**b**) the crystal structure diagram’s side view along different axes (**d**) An AFM image of a 6-nm-thick SnSe nanosheet; (**c**) optical image of SnSe NSs of various thicknesses (**e**) SnSe crystal HRTEM picture; (**f**) SAED pattern. Reproduced from [49] under permissions from copyright clearance center.

They reported that the orthorhombic monolayer structure of α-SnSe has four atoms in the unit cell organized in a rectangular lattice, with a = 3.95 Å and b = 4.82 Å as the relaxed lattice parameters. With a height of h = 2.59 Å, the puckered structure of α-SnSe resembles zigzag chains along the *y*-axis. They developed a symmetric lattice vector a = b = 3.78 Å for 2D hexagonal β-SnSe, which has a buckled surface and retains sp3 character bonds. The rectangular unit cells of γ-SnSe, δ-SnSe, and ε-SnSe, the other polymorphs, have four, eight, and eight atoms, respectively. It is evident from Figure 15’s top view that the five allotropes with comparable structures had identical Wigner-Seitz cells. Finally, they concluded that all five honeycomb structures maintained their stabilities. According to Yu et al. [49], weak van der Waals forces stack adjacent SnSe layers along the *c*-axis; as a result, mechanical separation can be accomplished in this direction easily (Figure 16a). The SnSe flake’s crystal structure is depicted in Figure 16b along the *a*- and *b*-axes, which stand for the in-plane directions. Figure 16d displays an AFM image of 6-nm-thick SnSe, which corresponds to the 10 layers of SnSe NSs along the *c*-axis, while Figure 16c displays an optical image of SnSe NSs of various thicknesses. The HRTEM image of the SnSe crystal is displayed in Figure 16e, where the lattice spacings along the *a*- and *b*-axes, which correspond to the (011) and ($00\bar{1}$) planes, respectively, are roughly 0.3 nm. According to their report, the angle of intersection between the (011) and ($01\bar{1}$) planes is around 89° [49].

### 3.2. SnS Structure

**Figure 17:**
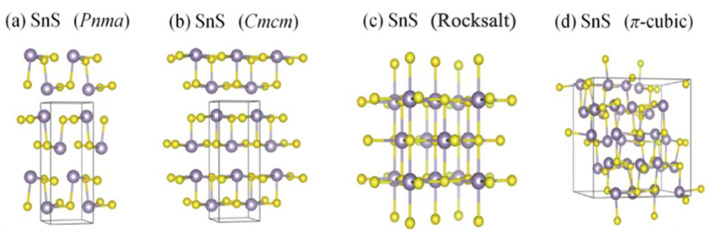
Optimized structure of (**a**) Pnma, (**b**) Cmcm, (**c**) rock salt, and (**d**) π-cubic SnS produced by VESTA software. Reproduced from [111] under permissions from copyright clearance center.

Likewise, SnSe, layered SnS, also exists in five phases-orthorhombic Pnma and Cmcm structures and cubic *Fm*$\bar{3}$*m* (rock salt), *P*2_1_3 (π-cubic) and *F*4$\bar{3}$*m* (zincblende) structures [111]. Figure 17 shows the crystal structure of α-SnS, β-SnS, and other phases such as π-SnS, zincblende, and rock salt structures [111,123,124].

**Figure 18:**
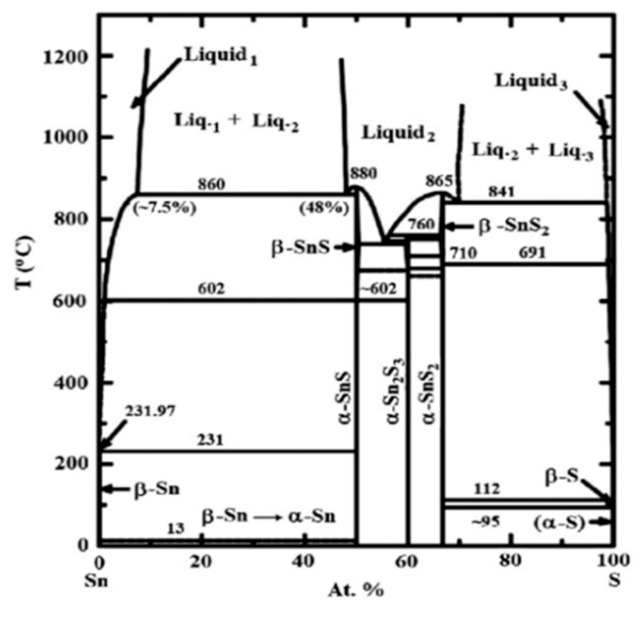
Phase diagram of SnS. Reproduced from [141] under permissions from copyright clearance center.

SnS usually exists in the orthorhombic α-SnS phase at room temperature [111]. Unlike SnSe, SnS shows an order displacive phase transition from orthorhombic α-SnS to orthorhombic β-SnS at 887 K, which belongs to the Cmcm phase. Figure 18 shows the phase diagram of SnS, which shows trends similar to those of SnSe [141]. SnS exhibits both n-type and p-type semiconductor characteristics [142]. The lattice parameters of α-SnSe are a = 11.32 Å, b = 4.05 Å, and c = 4.24 Å. β-SnS has the lattice parameters of a = 4.148 Å, b = 11.480 Å, and c = 4.177 Å [125].

Abutbul et al., π-SnS has a lattice parameter of a = 11.595 Å. π-SnS contains 64 atoms in its unit cell [124].

SnS orthorhombic Pnma and cubic rock salt structures have a bonding similar to SnSe, as previously described [120]. The π-cubic structure was reported as a metastable phase of SnS [111,124]. The zincblende phase of SnS has been reported as a high-energy, dynamically unstable phase [128,143]. It had been reported that the zincblende SnS can be considered as a misassignment of the π-cubic phase [111]. Although the rock salt phase of SnS has been reported as high-energy and dynamically unstable, under some circumstances, like epitaxial growth, it can be stabilized [144]. Mariano et al. reported epitaxial growth of rock salt phase SnS film under high pressure [126]. Table 2 summarizes the phases of multilayer SnS with their lattice constant.

### 3.3. SnTe Structure

**Figure 19:**
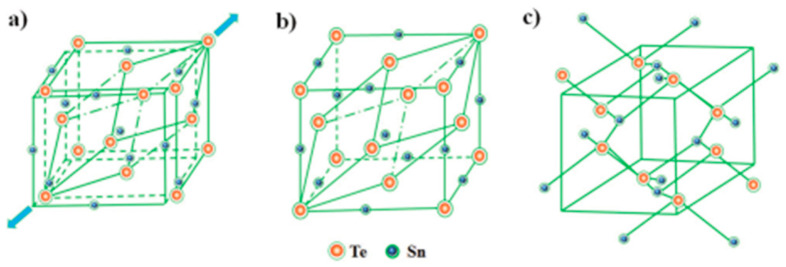
The unit cell of the three phases of SnTe. (**a**) α-SnTe, (**b**) β-SnTe, and (**c**) γ-SnTe. Reproduced from [127] under permissions from copyright clearance center.

In bulk form, SnTe can exist in three phases, namely-α, β, and γ-SnTe. α-SnTe has a rhombohedral or orthorhombic structure with lattice parameters of a = 6.235 Å and α = 89.895 Å, and its space group is R3m. α-SnTe is a low-temperature phase and exists below 100 K [127]. β-SnTe has a cubic structure that exists above 100 K and is stable at room temperature and pressure [85]. It has a lattice parameter of a = 6.318 Å and α = 90 Å and belongs to the space group of *Fm*$\bar{3}$*m*. β-SnTe can change to the α-SnTe phase when distortion occurs along the [111] direction of β-SnTe, forming the ferroelectric state [127]. This typically happens at a temperature less than 100 K and when the thickness is less than four atomic layers [26]. At the ferroelectric transition temperature Tc, two sublattices consisting of tin and tellurium atoms are shifted from each other along the [111] direction. As the concentration of Sn vacancy rises, Tc rapidly decreases [at 98 K] due to the screening action of charge carriers [128]. γ-SnTe has an orthorhombic structure at high pressure (18 kbar). When β-SnTe is under a high pressure of more than 18 kbar, it can transform to γ-SnTe phase. γ-SnTe has the space group Pnma and lattice parameters a = 11.95 Å, b = 4.37 Å, and c = 4.48 Å. The unit cell of the three SnTe phases is seen in Figure 19 [129]. SnTe can then be exfoliated in ambient circumstances [128]. 

**Figure 20:**
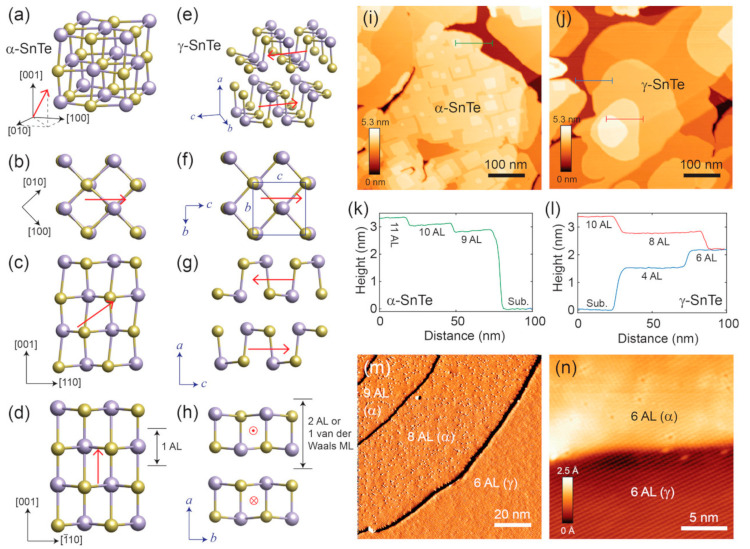
α and β phases coexisting in ultrathin SnTe sheets. (**a**–**d**) Ultrathin α and γ-SnTe films’ lattice structure. For clarity, the lattice distortion is emphasized. SnTe lattice structure (antipolar, space group Pnma) (**e**–**h**). Te atoms are golden, and Sn atoms are gray. The red arrows show the directions of spontaneous polarization. STM topography in the α-SnTe phase (**i**) and γ-SnTe phase (**j**) regions. Height profiles along the line segments shown in (**i**,**j**) are shown in (**k**,**l**). (**m**) Examining how the defect concentration differs in the SnTe nanoplate between the α and γ-SnTe phases. (**n**) Topography image with atomic resolution at the boundary between two phases on the same terrace. Reproduced from [129] under permissions from copyright clearance center.

Beyond these, Chang et al. reported the coexistence of α and β phases in ultrathin SnTe films [129]. When the γ-SnTe films are thicker than four atomic layers, and the temperature becomes 400 K, they transform into a rock salt structure. Using variable temperature scanning tunneling microscopy to characterize SnTe nanoplates, Chang et al. reported this phase change [129]. Figure 20 shows the co-existence of α and β phases in ultrathin SnTe films [129].

**Figure 21:**
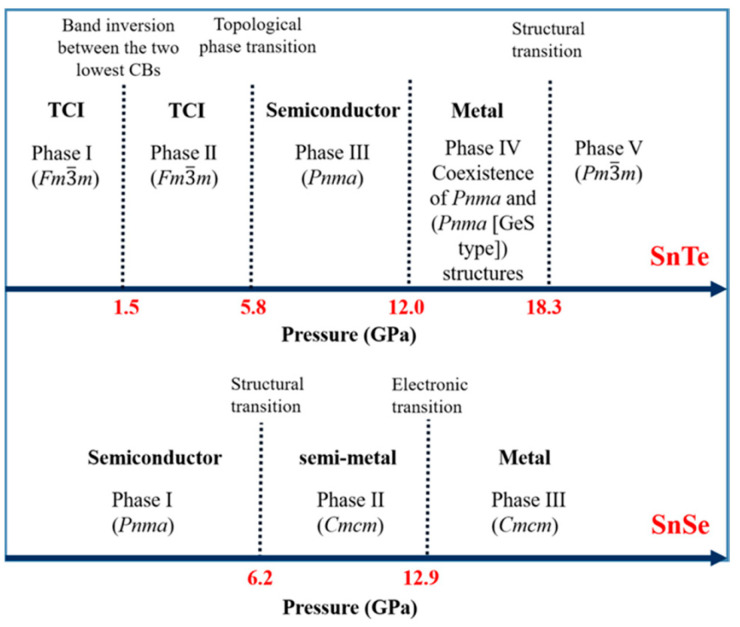
Phase diagram of SnSe and SnTe at different pressures. Reproduced from [146] under permissions from copyright clearance center.

**Figure 22:**
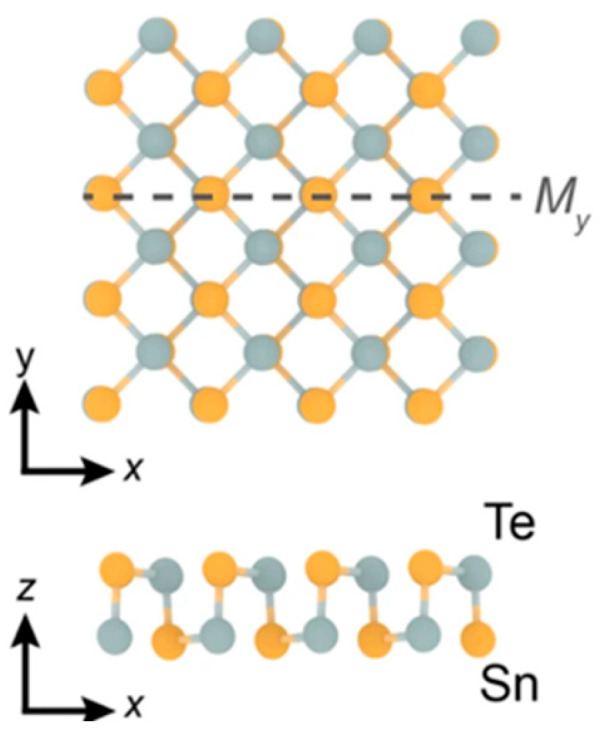
Atomic geometry of puckered rectangular lattice of monolayer SnTe with the top view (**upper diagram**) and side view (**lower diagram**). Reproduced from [145] under permissions from copyright clearance center.

SnTe is a Sn-deficient, narrow-gap (0.2 eV) semiconductor when it is in bulk form. It becomes a p-type material as a result of additional Te atoms producing two holes in the valence band [145]. Bulk SnTe can exist in five phases as the pressure changes. At 1.5 GPa pressure, there are changes to the charge density isosurfaces and the band inversion between the two lowest energy conduction bands, according to Pal et al. A shift in structural symmetry causes a topological phase transition to occur at 5.8 GPa pressure. When the pressure increases to 12 and 18.3 GPa, SnTe changes its crystal and electronic band structures. Figure 21 shows the phase diagram of SnTe and SnSe at different pressures [146]. Sn and Te atoms alternate in a puckered rectangular lattice to form the SnTe monolayer. Figure 22 displays the monolayer SnTe atomic geometry [145].

Table 2 summarizes the phases of SnTe multi and monolayer with their lattice constant.

### 3.4. PbS Structure

**Figure 23:**
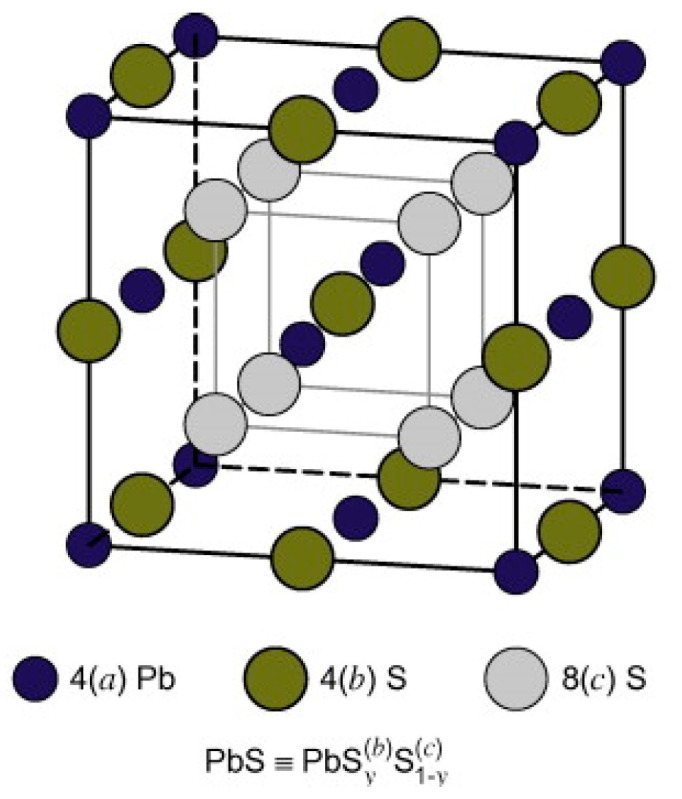
Crystal structure of PbS. Reproduced from [147] under permissions from copyright clearance center.

There has not been much work done to investigate the properties of monolayer PbS, primarily because the buckling of Pb and S sublattices in opposite out-of-plane directions makes a flat monolayer unstable [133]. PbS mainly shows a cubic rock salt crystal structure similar to NaCl [130]. It also shows an orthorhombic structure at high pressure [131]. It has a crystal structure of DO3 or B1 type and is a member of the Fm$\bar{3}$m space group. Its lattice parameter is a = 5.936 Å [130]. Under a pressure of 2.5 GPa, the B1 crystal structure of PbS changes its phase to an orthorhombic B33 crystal structure [131]. In the case of PbS nano-crystals, the transition occurs at higher pressure (3.0 GPa). This phase has a crystal structure that belongs to the space group of Pnma. PbS nanofilms can also exist in orthorhombic crystal structures under normal conditions. After one hour of reaction conducted by chemical synthesis, its lattice parameters are found to be a = 10.96 Å, b = 4.02 Å and c = 4.28 Å [132]. Figure 23 displays the PbS crystal structure [147].

**Figure 24:**
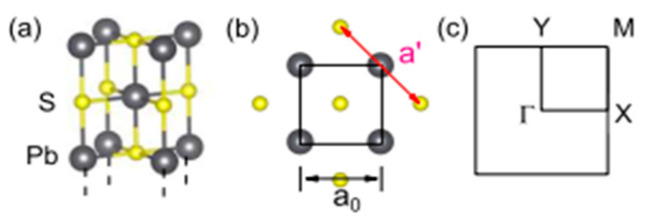
(**a**) Side view, (**b**) top view of the lattice structure, and (**c**) the first few layers of PbS (001) Brillouin zone (BZ). The bulk lattice constant in (**b**) is denoted by a′ and the few-layer lattice constant by a_0_. [133] under permissions from copyright clearance center.

**Figure 25:**
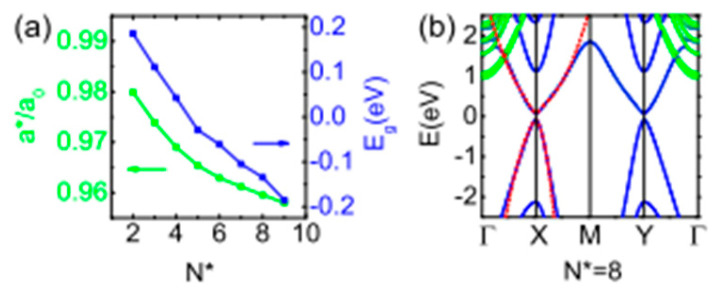
The band structures and lattice constants from the KF-PbS monolayer (**a**) vary according to the ratio of the flat freestanding monolayer’s lattice constant (a_0_) to the lattice constant of a*, and (**b**) the band structures with N* = 8 and the energy bandgap are associated with the KF layer number (N*). The colors blue and green represent the contribution from PbS and KF. The fitted bands using the effective k.p. Hamiltonian equation are represented by the red lines. Reproduced from [133] under permissions from copyright clearance center.

Wan et al. [133] investigated several properties of a few layers thick PbS (001), focusing on the monolayer. A few layers of PbS (001) are shown, together with the lattice structure and the first Brillouin zone (BZ) in Figure 24 [133]. They discovered that the interlayer spacing between the few layers, which experiences periodic contractions and extensions, is 4.069 Å and that the predicted lattice constant of bulk PbS is a = 6.000 Å. According to their findings, each odd-layer PbSe layer has an additional single-layer or trilayer block in the core. Additionally, they showed that a few PbS layers can be made stable in an experiment by being sandwiched between the shells of CdS. Because of this, they proposed an improved structure consisting of the monolayer of PbS sandwiched between two KF layers. To avoid the unbearable computational cost, they adopted the PBE + SOC method to show the band structure of KF-PbS monolayer-KF. Less than 4.5% of the PbS monolayer is under strain, as shown in Figure 25. They concluded that the PbS monolayer mainly contributes to the electronic states near the Fermi level. At the same time, due to their wide band gaps, the contribution of the KF layers is minimal (see Figure 25). 

According to Qadri et al. [137], around 375 K, lead sulfide (PbS) undergoes a weak phase transition from the B1 type structure to the B3 type cubic structure (space group F43m). During this transition, there is a noticeable increase in the relative intensity of the (220) diffraction reflection compared to that of the (200) reflection. Since it is metastable, at 300 K, the B3 structure and the phase with the B1 type structure can coexist. Table 2 summarizes the PbS multi and mono-layer phases with their lattice constants. 

### 3.5. PbSe Structure

**Figure 26:**
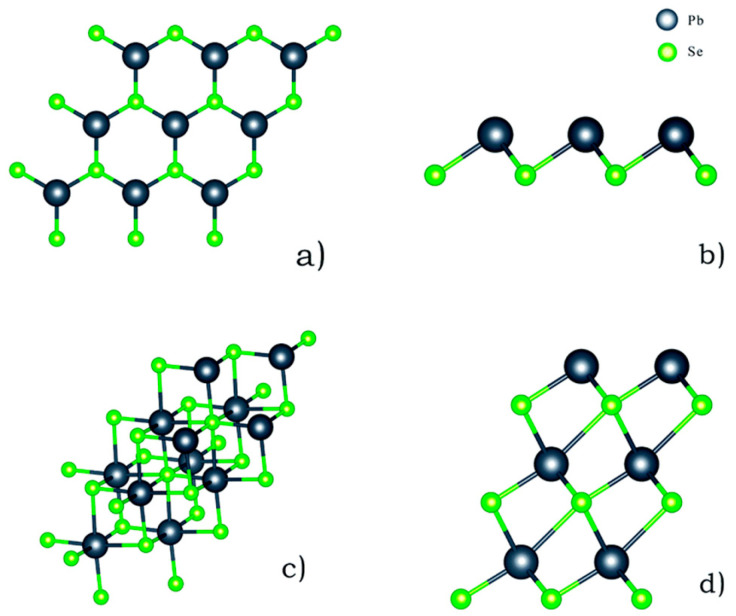
PbSe structures with a single layer and several layers ((**a**) is PbSe in a single layer; (**b**) is a side view of it; (**c**) is PbSe in a multi-layer; (**d**) is a side view). Reproduced from [135] under permissions from copyright clearance center.

PbSe has a structure similar to PbS. It crystallizes into a cubic Nacl structure (face-centered cube). It has a crystal structure of B1 and belongs to the space group of Fm$\bar{3}$m [148]. It has a lattice constant of a = 6.12 Å [149]. Under high pressure, PbSe changes its structure from cubic to orthorhombic type. At a pressure of ~9.5GPa, PbSe changes its crystal structure to orthorhombic, which belongs to the space group of Pnma, and its lattice parameters are a = 11.18 Å, b = 4.17 Å and c = 4.05 Å. PbSe also shows CsCl structure at high pressure (above 16 GPa), which belongs to the B2 crystal structure and space group of Pm$\bar{3}$m. The lattice constant of CsCl PbSe is a = 3.56 Å. At a pressure of ~9.5 GPa, PbSe’s orthorhombic phase also belongs to the space group of Cmcm. The PbSe combination is discovered to be a metal, but the second one is a semiconductor with a narrow energy bandgap. This is the primary distinction between the electrical characteristics of the Cmcm and Pnma phases (E_G_ = 0.12 eV) [134]. The structure of single and multilayer PbS can be seen in Figure 26 [135].

**Figure 27:**
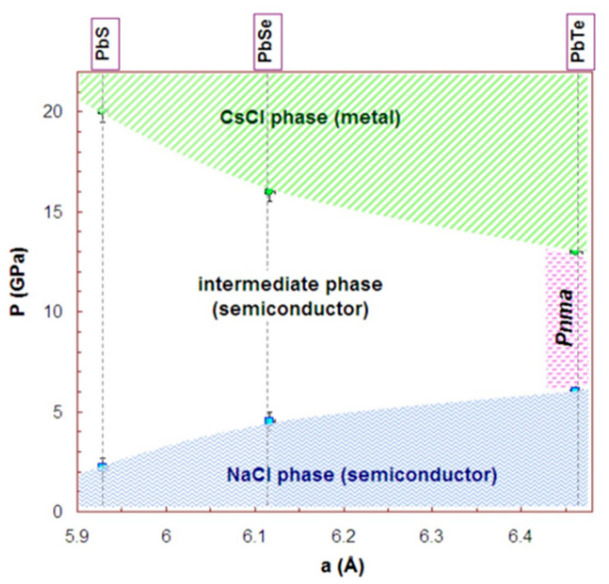
Various PbS, PbSe, and PbTe phases at different pressures with lattice constant. For PbTe, the structure of the intermediate phase is Pnma symmetry, while PbS and PbSe remained obscure. Reproduced from [114] under permissions from copyright clearance center.

The TE characteristics of a puckered PbSe monolayer that resembles a honeycomb were explained by Tang et al. [136] using DFT calculations and the Boltzmann transport approach theory. They showed that Pb and Se atoms in a honeycomb-like structure formed the puckered PbSe monolayer in a zigzag pattern. According to their findings, the PbSe monolayer (P3m1 space group) has equilibrium lattice parameters of a = b = 4.091 Å. According to a report, PbX [X = S, Se, and Te] passes through a few low-symmetry intermediate high-pressure phases [114] (see Figure 27). Ovsyannikov et al. [150] demonstrated that for PbSe, the transition between B1 and B2 happens with two intermediate phases. After analyzing the thermopower and other data, they mentioned that these two phases are n-type and p-type, with n-type conductivity dominating. P-type conductivity, on the other hand, is optional. Additionally, they claimed that both phases have semiconducting electrical conductivity. Table 2 summarizes the phases of the PbSe multi and monolayer with their lattice constants.

### 3.6. PbTe Structure

**Figure 28:**
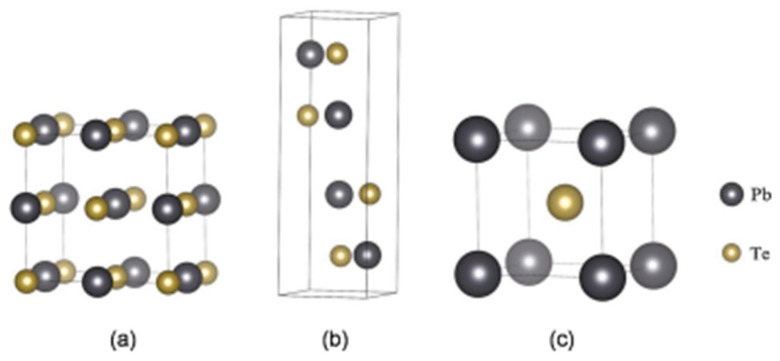
Unit cells of PbTe ((**a**) Fm$\bar{3}$m space group PbTe, (**b**) Pnma space group PbTe, (**c**) Pnn space group PbTe). Reproduced from [151] under permissions from copyright clearance center.

PbTe, under ambient conditions, crystallizes to cubic (B1) NaCl structure, with space group Fm3m and lattice constant a = 6.461 Å [138]. PbTe also exists in orthorhombic Pnma and Pmm and CsCl space groups [139]. Unit cells of *Fm*$\bar{3}$*m*, Pnma, and Pnn PbTe are shown in Figure 28 at 9.5 Gpa. PbTe changes its phase from cubic PbTe to orthorhombic PbTe (Pnma). The lattice constants of orthorhombic PbTe are a = 8.11 Å, b = 4.50 Å, and c = 6.26 Å. At higher pressure (above 24.7 GPa), PbTe changes its space group to CsCl. The lattice constant of CsCl PbTe is a = 3.57 Å [140]. For PbTe, the intermediate phase, while this material transitions between B1 and B2 phases under the influence of pressure, is identified as Pnma symmetry [150,151]. 

The crystal structures of several tin-and lead-based chalcogenides, such as SnSe, SnS, SnTe, PbS, and PbSe, have been covered in this section. Every material’s distinct structural features immediately impact its electrical and physical properties. The anisotropic thermoelectric and mechanical properties of SnSe and SnS both derive from their adoption of layered orthorhombic structures. Conversely, SnTe has a rock-salt crystal structure akin to lead chalcogenides, providing superior symmetry and being a viable option for thermoelectric purposes. The cubic rock-salt structure shared by PbS and PbSe is responsible for their stable electrical characteristics and widespread use in thermoelectric and optoelectronic devices.

## 4. TE Properties

Group IVA-VIA 2D elements are known to exhibit TE properties. TE materials use the Peltier or Seebeck effects to turn heat into electricity. Similar to gas molecules, the charge carriers in metals and semiconductors are mobile and carry both heat and charge when one side of the material experiences an increase in temperature. A net charge will result from this particle accumulation at the cold end; this net charge will be positive for holes (h^+^) and negative for electrons (e^−^). Consequently, an electric potential and a repulsive electrostatic force will be produced. The chemical potential for diffusion and the electrostatic repulsion come to an equilibrium in the system. The Seebeck effect is the name given to the electric potential difference (ΔV) generated by the temperature differential (ΔT). The figure of merit (zT) = α^2^Tσ/(κE + κL), where α, σ, κE, κL, and T are the Seebeck coefficient, electrical conductivity, lattice thermal conductivity, electrical, thermal conductivity, and absolute temperature, respectively, define the performance of TE materials. The power output is determined by the electrical current produced by the heat flow, which determines the voltage (ΔV = α∆T) from the Seebeck effect [152]. For large-scale applications, TE materials must have a high conversion efficiency. Low thermal conductivity, High electrical conductivity, and a big thermopower—the absolute value of the α—are necessary for a material to have a high zT. Various parameters must be tuned to maximize zT since these transport characteristics depend on connected material qualities. 

The underlying geometrical patterns and energy band structures of Sn and Pb-based materials play a crucial role in determining their TE capabilities. For example, the material’s thermal conductivity, Seebeck coefficient, and electrical conductivity all directly impact the TE figure of merit. The material’s crystal shape and electrical structure are inextricably linked to these factors. The geometrical structure of these materials is crucial to phonon transport, which in turn influences thermal conductivity. Anisotropic bonding and atomic arrangements of layered materials, such as SnSe and PbS, provide substantial phonon scattering in particular crystallographic orientations, greatly reducing heat conductivity and improving TE performance. Furthermore, phonon scattering is further enhanced by decreasing the dimensionality of thin films or nanosheets, which lowers thermal conductivity without appreciably compromising σ. The Seebeck coefficient and electrical conductivity are significantly influenced by the energy band structure, which is frequently dictated by optical and electronic characteristics. A material’s bandgap determines its carrier concentration and mobility; high Seebeck coefficients and adequate electrical conductivity are made possible by an ideal bandgap. For example, SnSe has a good band structure that balances energy dispersion and carrier concentration close to the Fermi level, enabling strong TE performance. The higher Seebeck coefficient is also a result of the density of states (DOS) close to the Fermi energy, which increases the figure of merit even more. This section offers a thorough examination of the interactions among geometrical structure, energy band structure, and TE characteristics in order to maximize the thermoelectric efficiency of Sn and Pb-based materials.

To achieve a considerable zT value, the semiconductor should be doped with only one type of impurity—either n-type or p-type. If both types of conductivities were used to cancel out the induced Seebeck voltages, both charge carriers would have to move to the cold end. There are several factors and terms on which the zT value depends. The first one is the Seeback coefficient, which is defined as follows:

(3) $$\alpha =\frac{8\pi^{2}k_{B}^{2}}{3eh^{2}}m^{*}T{\left(\frac{\pi }{3n}\right)}^{2/3}$$

Here, k_B_, h, m*, e, T, and n are the Boltzmann constant, Planck’s constant, effective mass of the material, carrier charge, absolute temperature, and doping concentration, respectively. Materials with higher effective mass would yield higher zT values. However, the m* value would decrease the mobility of the carrier and, hence, the electrical conductivity. Effective mass and mobility have a complicated relationship influenced by anisotropy, different scattering mechanisms, and electronic structure [10]. The material for TE applications should be chosen carefully to optimize the values of the effective mass of the dominant carrier (n or p-type) and the mobility high enough to obtain a higher zT value. This is a challenging job. The link between conductivity, Seeback coefficient, and carrier concentration is intricate [18]. Electrical conductivity would rise in tandem with a drop in the Seeback coefficient value as carrier concentration increased. The following relationship expresses the link between electrical conductivity, electron concentration, and carrier mobility:

σ = neµ(4)

Therefore, the value of the doping concentration should be selected carefully to maintain a balance between conductivity and the Seeback coefficient. The semiconductor’s doping concentration also influences a material’s thermal conductivity value. The single independent parameter for the TE characteristics, lattice thermal conductivity, negatively correlates with the zT value. The expression for electrical thermal conductivity is

κ_e_ = σLT = neµLT (5)

where L is the Lorenz factor, 2.4 × 10^−8^ J^2^ K^−2^ C^−2^ for free electrons. 

Carrier concentration has a significant impact on the Lorenz factor. According to reports, materials with low carrier concentration can lower their κe by up to 20% relative to the free electron value. Via the inclusion of a bipolar term to the thermal conductivity, mixed carrier conduction can also increase the degree of uncertainty in the κe value. Low heat and strong electrical conductivity are required to achieve a high zT value. Once more, this calls for the two conductivity mechanisms to be optimized. α alone determines zT.

**Figure 29:**
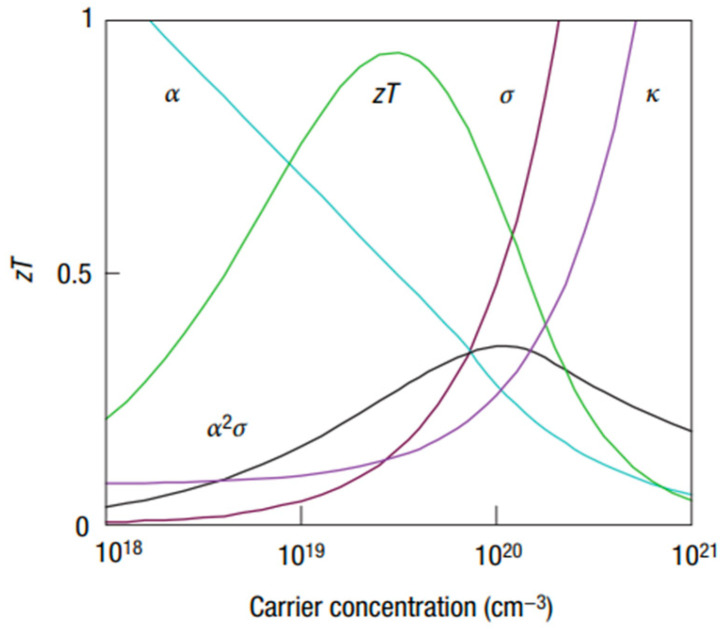
Dependence of TE figures of merit on various parameters. Reproduced from [152] under permissions from copyright clearance center.

The aforementioned equations and discussions demonstrate that improving zT just through parameter optimization of S, σ, and κE is challenging because of their significant coupling impact. Figure 29 [152] displays the change of zT and its reliance on other parameters. The zT value may be adjusted to reach its peak at various temperatures by adjusting the carrier concentrations. In semiconductors, carrier densities between 10^19^ and 10^21^ carriers per cm^3^ are usually where the most significant value of zT occurs. 

Numerous theoretical forecasts indicated that electron charge carriers trapped in a quantum state might significantly increase the TE efficiency [153]. Quantum confinement is achieved by creating a structure with progressively narrower electron energy bands. For these types of structures, the confinement increases as the dimensionality decreases and the effective mass of carriers increases. Larger effective mass produces large α [153]. The most effective approach to increasing the zT value is perhaps by decreasing the thermal conductivity [152]. According to reports, three distinct approaches have been used to reduce the heat conductivity of the lattice. The foundation of all three strategies is searching for crystal-form materials that disperse phonons without appreciably altering electrical conductivity. Regarding this, the first method involves alloying, rattling structures, or point defects like interstitials or vacancies to disperse phonons within the unit cell [91]. The second tactic is to split the electron-crystal from the phonon-glass by forming a complicated crystal structure. Without affecting the electron-transport region’s crystallinity, this intricate structure will aid in creating a phonon glass. Instead of quick transfer via phonons, thermal conductivity in the case of glass is understood as a random path of energy across a lattice. The idea of a minimum thermal conductivity will result from this [154]. Thermal conductivity will decrease if crystal materials have flaws. The phonon scattering is strongly boosted as long as the defect size matches the typical free path range. This can dramatically lower the lattice thermal conductivity and enhance the TE characteristics of PbTe [155]. Nanostructures with superlattices can be formed by mixing multiphase nanocomposites. The third strategy is based on scattering phonons at the interfaces of the superlattice [153]. 

In addition to single materials, hetero-nanostructures have gained significant attention due to their ability to further enhance thermoelectric performance, particularly by drastically reducing thermal conductivity. The nanostructuring approach introduces interfaces and grain boundaries that scatter phonons more effectively than in bulk materials, lowering the thermal conductivity without significantly impacting electrical conductivity. This review will also explore hetero-nanostructures, highlighting their potential advantages over single-material systems, as they offer an additional pathway to optimize thermoelectric efficiency and improve overall material performance for energy conversion applications. To better understand the thermal resistance at amorphous/epitaxial interfaces, a study by Ishibe et al. [156] focused on the problems associated with heat dissipation in nanoscale electronic applications. In the amorphous germanium sulfide/epitaxial-lead telluride hetero-nanostructure, the authors claim a shallow interface thermal resistance due to the lack of a complicated interface layer. With the help of ab initio calculations, it is possible to optimize heat conduction in nanoscale systems by identifying the high phonon transmission caused by group velocity similarities and overlapping phonon density-of-states between the crystalline and amorphous layers. In another article [157], the intriguing nanostructured phase-change materials for non-volatile memory applications—the GeTe–Sb_2_Te_3_ superlattices—are examined. In contrast to previous predictions, the study’s use of electron microscopy and MBE revealed that the film is made up of rhombohedral GeSbTe and van der Waals bound layers of Sb_2_Te_3_. The van der Waals heterostructure is thermodynamically favorable, implying that the superlattice converts to bulk GeSbTe during annealing, providing fresh insights into phase-change mechanisms and memory applications. Additionally, the thermoelectric properties of (GeTe)_x_(AgSbTe_2_)_100−x_ compounds were investigated by Yang et al. [158]. The results showed that TAGS-90 displayed a lower zT of 0.50 due to a more significant carrier concentration, whereas TAGS-75, TAGS-80, and TAGS-85 samples reached high zT values of 1.50–1.53 at 720 K. In the high zT samples, high-resolution microscopy identified nanoscale regions (~10 nm) that boosted mid-frequency phonon scattering and lowered lattice thermal conductivity. This nanoscale structure is proposed to decrease thermal conductivity in TAGS compounds, thus improving thermoelectric performance. Regarding enhancing thermoelectric performance, Sn and Sb-based chalcogenides have several advantages over GeTe, including reduced thermal conductivity, variable carrier concentration, and better thermal stability [159]. It has been demonstrated, for instance, that SnSe has substantially less thermal conductivity than GeTe, which results in improved heat conversion efficiency. Moreover, materials based on Sb_2_Te_3_ frequently exhibit greater mechanical flexibility and doping control, which, in some circumstances, can result in increased zT values. Sn and Sb-based chalcogenides frequently show better thermal and electrical properties when compared to different types of GeTe, such as bulk GeTe, polycrystalline GeTe films, and epitaxial GeTe films on Si and sapphire substrates [160,161]. Bulk GeTe usually has a high heat conductivity, although strain-induced effects can be seen in epitaxial GeTe films, which can marginally enhance performance. However, Sn and Sb-based chalcogenides are competitive and, in some cases, even better than GeTe in thermoelectric applications due to their superior ability to suppress thermal conductivity through phonon scattering at grain boundaries or interfaces and their ability to achieve high Seebeck coefficients [162]. For example, strain enhances the performance of epitaxial GeTe films on Si or sapphire substrates, although SnSe and Sb_2_Te_3_ materials often perform better in terms of total thermoelectric efficiency because of their superior electrical characteristics and phonon scattering mechanisms [117].

### 4.1. SnS TE Properties

**Figure 30:**
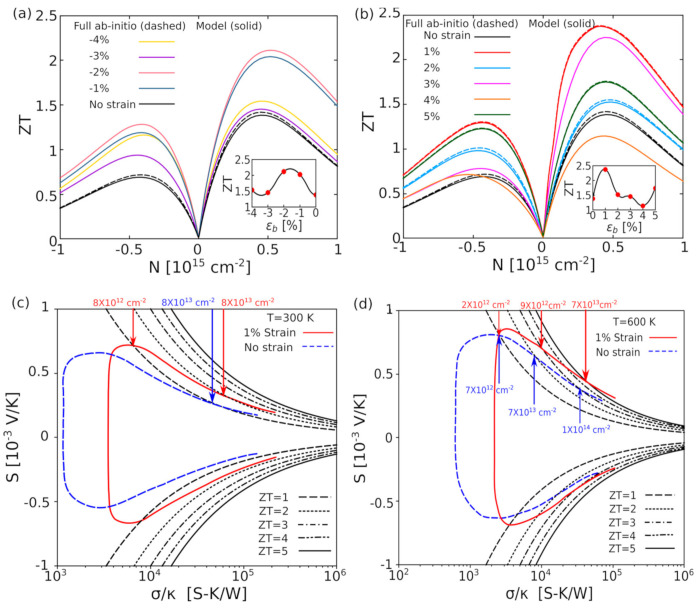
Changes in room-temperature zT with carrier concentration for SnS monolayers under (**a**) compressive strains (=−4%, −3%, −2%, and −1%) in the absence of strain and (**b**) tensile strains (=1%, 2%, 3%, 4%, and 5%) in the presence of strain and no strain. Whereas dotted lines show zT computed using full ab initio lattice thermal conductivity, solid lines show zT derived using the semiempirical model for lattice thermal conductivity. Both Figures’ insets display the fluctuation of maximum zT with compressive and tensile strains. α variation at (**c**) 300 K and (**d**) 600 K as a function of electrical conductivity to total thermal conductivity ratio. The red line shows the SnS monolayer at 1% tensile strain, while the blue dashed line shows the unstrained SnS monolayer. Iso-zT curves are represented by the black lines. Reproduced from [167] under permissions from copyright clearance center.

The spontaneous polarization is caused by the dipole moment in the armchair direction in monolayer SnS [163]. So, the monolayer SnS is expected to demonstrate ferroelectric behavior in that direction. Moreover, Gupta et al. [164] examined the TE characteristics of monolayer SnS using DFT and the Boltzmann transport equation. From a phonon scattering perspective, they discovered a clear difference between bulk materials and this monolayer, where typical processes play a larger role. They also found that the zT values of monolayer SnS at 600 and 300 K are, respectively, ~5 and ~1.36, which is 33 times higher than its bulk equivalent. Li et al. [165] synthesized SnS NSs through WCS. They increased the carrier concentration of SnS NSs by increasing Sn vacancies by doping SnSe and embedding Ag atoms. This led to a decrease in the lattice thermal conductivity at 873 K. They observed that on SnSe-Ag doped SnS NSs, the maximum zT value was 0.8 at 873 K. Ju et al. [68] synthesized porous SnS NSs coated with conductive PANI and reported their TE properties. At normal temperature, SnS crystals can form in a highly anisotropic layered orthorhombic structure (Pnma). At high temperatures (873 K), these crystals transition to the Cmcm structure, which exhibits a high zT value due to limited thermal conductivity. By creating piezoelectric nanogenerators, Khan et al. [62] investigated the piezoelectric characteristics of the monolayer SnS. After that, the devices were examined by applying mechanical strains in different ways. They investigated the piezoelectric property of monolayer SnS using piezoresponse force microscopy (PFM). They measured the piezoelectric coefficient and carrier mobility of atomically thin SnS and found that they were around 35 cm^2^V^−1^s^−1^. According to their findings, the SnS monolayer-fabricated piezoelectric nanogenerators showed a peak output voltage of about 150 mV. Li et al. [166] investigated the SnS/SnSe heterojunction using DFT. Their findings indicated that the reduced effective mass, mild electron-phonon scattering, and improved elastic modulus of the 2D SnS/SnSe heterojunction make it a highly desirable candidate for 2D electronic devices. Beyond size control, these results suggest that heterointerlayer coupling is a promising strategy for high-efficiency TE materials. Using first-principles calculations and the Boltzmann transport equation, Gupta et al. [167] reported improving the zT value of the SnS monolayer by applying uniaxial compressive and tensile loads. They discovered that the strains (both tensile and uniaxial compressive) were dynamically stable and ranged from –4% to 5% along the armchair direction. They obtained a high power factor for 1% strain related to p-type carriers, approximately 1.8 times more than in the case without strain. When a minimum of 1% tensile strain is applied, they discover an approximately 77% enhancement in the zT value for p-type carriers and an approximately 86% increment in the zT for n-type carriers concerning equilibrium. The fluctuation of the SnS monolayer at room temperature is seen in Figure 30 [167]. 

**Figure 31:**
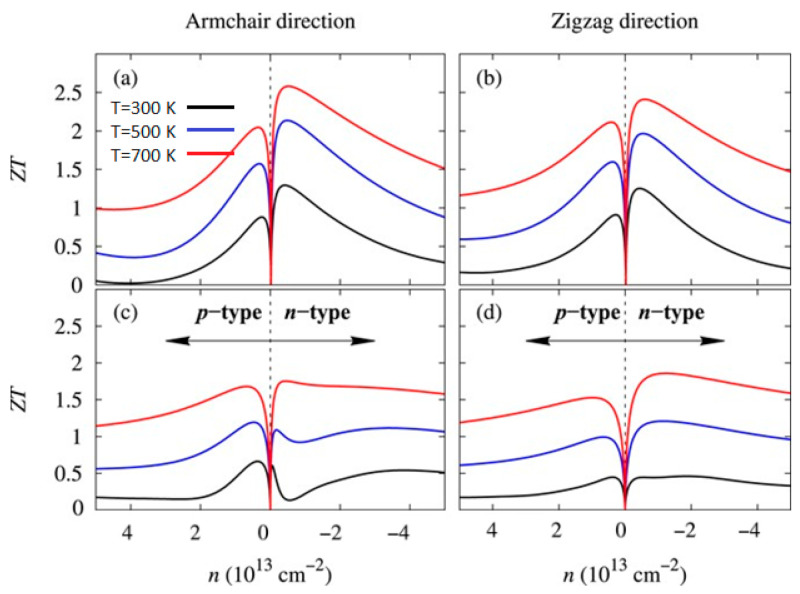
Values of zT were computed as a function of the carrier concentration (n) along armchair and zigzag directions for the monolayer of (**a**,**b**) SnSe and (**c**,**d**) SnS at temperatures of 300 K, 500 K, and 700 K. Reproduced from [168] under permissions from copyright clearance center.

Using DFT in conjunction with Boltzmann transport theory, Shafique et al. [168] investigated 2D monochalcogenides’ TE and phonon transport properties. The researchers examined these compounds’ electronic configurations, α, electrical and lattice thermal conductivities, and the monolayer figures of merit. High zT was discovered at 700 K along the armchair and zigzag directions for SnSe (zT = 2.63, 2.46) and SnS (zT = 1.75, 1.88). They confirmed the dynamic stability of the compounds by using phonon dispersion simulations. They discovered that although the electrical and α are high, the computed lattice thermal conductivities are low. Due to its low lattice thermal conductivity, α, and high electrical conductivity, SnSe exhibited the highest zT of 2.63. A high zT of 1.88 is anticipated in the zigzag direction, for instance, SnS. For the monolayer of SnSe and SnS at temperatures of 300 K, 500 K, and 700 K, Figure 31 displays the computed zT as a function of the carrier concentration [168].

**Figure 32:**
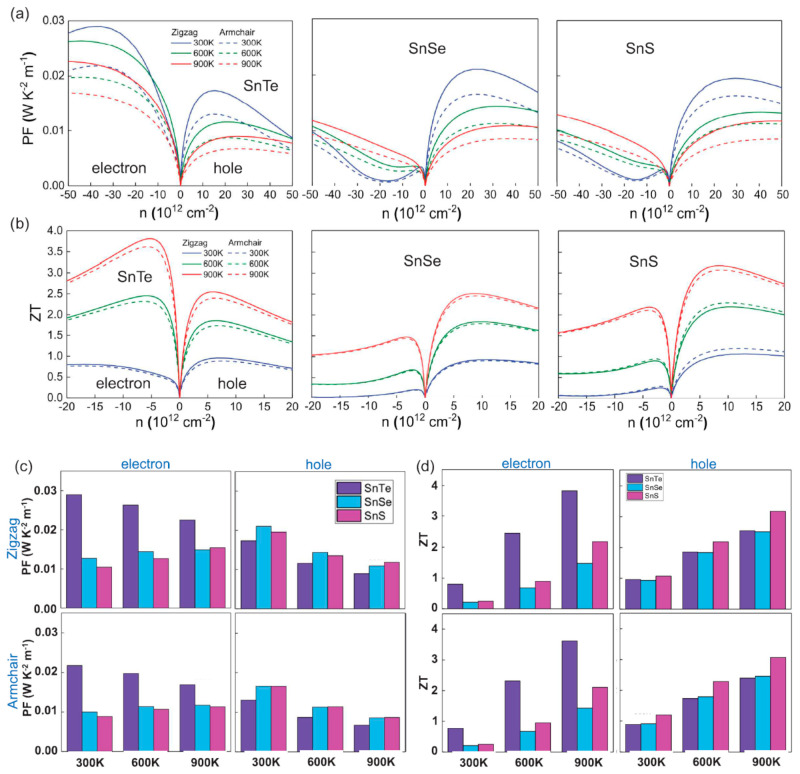
β′-SnX’s TE performance. zT of SnX (X = S, Se, Te) varying with the doping level n at various temperatures (**a**,**c**) and power factor (PF) (**b**,**d**). The dashed and solid lines uphold the armchair and zigzag directions. The maximal PF and zT at the ideal doping level are displayed in (**c**,**d**) as a function of temperature and crystal orientation. Reproduced from [172] under permissions from copyright clearance center.

Pandit et al. [169] analyzed the structural, electronic, and TE characteristics of 2D SnS monolayers semi-classical Boltzmann transport theory combined with DFT. It was discovered that SOC had a major impact on the electrical structure and characteristics. At 700 K, the highest zT value for n-type SnS was anticipated to be 1.04. Using first principle calculations, Fang et al. [170] discovered that the SnS monolayer exhibits significant anharmonic phonon activity and modest group velocities. For SnS, they discovered a zT value of around 0.13–0.89 at 300 to 700 K, respectively. To determine the TE performance of SnS monolayers, Sandonas et al. [171] employed a variety of methods to calculate the band structures for phonons and electrons. The methodologies used Green’s function-based transport methodologies. At room temperature, they determined the zT value for both transport directions. Dong et al. [172] used VASP in conjunction with Universal Structure Predictor Evolutionary Xtallography (USPEX) to investigate the optical and electrical characteristics of monolayer SnS. Using USPEX, they discovered a new hexagonal phase that they termed β′-SnX (X = S, Te). They discovered that the most stable phases of SnS are also observed to be energetically near the β′ phases. With a practically achievable carrier concentration of 5 × 10^12^ cm^−2^, they achieved a high TE behavior in the β′-SnS phases, with the zT reaching as high for SnS at temperatures 300 K to 900 K as shown in Figure 32. They attributed the low thermal conductivity and high-power factor to the quantum confinement of 2D substances and band convergence around the Fermi level and the low elastic constants resulting from strong lattice anharmonicity and weak inter-Sn bonding strength.

### 4.2. SnTe TE Properties

**Figure 33:**
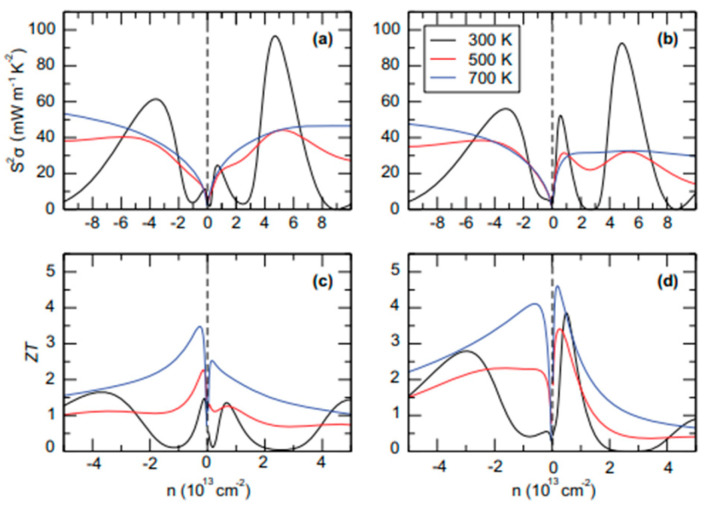
Variations of power factor and TE zT of SnTe bilayer with changing temperature along the armchair (**a**,**c**) and zigzag (**b**,**d**) directions. Reproduced from [178] under permissions from copyright clearance center.

Relying on a synthesis of Boltzmann transport theory and DFT, Li et al. [173] conducted methodical investigations on the electronic, TE, and phonon transport properties of monolayer SnTe. They discovered that the monolayer is dynamically stable. At a modest carrier concentration of 10^20^ cm^−3^, they obtained zT values of the armchair orientation, resulting in a doping of n-type of 2.9 and p-type of 2.2. For the n-type SnTe monolayer, they measured a maximum power factor along the armchair direction. They claimed this value was nearly twice just as high as that in a zigzag pattern. The elasticity criterion and phonon dispersion computation were used by Dong et al. [172] to verify the structural stability. They discovered that the β′-SnTe is the most stable of all potential 2D phases of SnTe, including those available experimentally. With a practically achievable carrier concentration of 5 × 10^12^ cm^−2^, they achieved a TE behavior in the β′-SnTe phases, with a zT value of ~0.96 to 3.81 for SnTe at temperatures ranging from 300K to 900 K. The elasticity criterion and phonon dispersion computation were used by Dong et al. [172] to verify the structural stability. They discovered that the β′-SnTe is the most stable of all potential 2D phases of SnTe, including those available experimentally. With a practically achievable carrier concentration of 5 × 10^12^ cm^−2^, they achieved a TE behavior in the β′-SnTe phases, with a zT value of as high as ~0.96 to 3.81 for SnTe at temperatures ranging from 300 K to 900 K. Zhang et al.’s [174] first-principles computation and Boltzmann transport theory were used to examine the TE characteristics of SnTe monolayers. Their phonon analyses demonstrate that the ultralow thermal conductivity is caused by the avoidance of the crossing between the longitudinal acoustic phonons and the low-lying optical branches in the Γ-X and Y-Γ high-symmetry routes, as well as the coexistence of resonant bonding and weak bonding in PbTe monolayers. They concluded that these characteristics cause SnTe monolayers to have high zT values, which can exceed 1.58 at 900 K when there is an n-type doping concentration of 10^19^–10^20^ cm^−3^. Tang et al.’s [175] research on the TE performance of a 2D wrinkled SnTe monolayer used Boltzmann transport theory and first-principles computations. They used ab initio molecular dynamics simulations, the elastic modulus, and formation energy to extensively evaluate the high mechanical, dynamic, and thermal stabilities of the SnTe monolayer. They concluded that the p-type SnTe monolayer exhibits a zT of 1.44 at 900 K along the zigzag direction. Using the Boltzmann transport equation in conjunction with first-principles calculations, Liu et al. [176] could determine the TE characteristics of SnTe monolayers. According to their computed findings, SnTe monolayers have an indirect band gap in semiconductors and tiny effective masses, which result in electrical solid conductivities and great carrier mobilities. They observed that the lattice thermal conductivity of SnTe is relatively low due to considerable anharmonicity and low phonon group velocity. They determined that the ideal zT value was 2.61 at 500 K. Lubis et al. [177] computed the properties, including electrical and TE, of monolayer and bilayer SnTe using Density Functional Theory, which included the linearized Boltzmann transport equation with a constant relaxation time assumption. They demonstrated that the monolayer SnTe ideal figure of merit is higher than the bilayer SnTe. This highlights the importance of reducing the bulk SnTe to a single-layer thickness to improve TE performance. TE performance with zT > 1.6 is expected for the n-type monolayer buckling SnTe at T = 900 K. Bilayer SnTe exhibited strong electrical conductivity, low lattice thermal conductivity (<1.91 W-m^−1^K^−1^), and high α (>400 μVK^−1^), according to Pandit et al.’s first-principle calculations [178]. They projected that for carrier concentrations in the range of 10^12^–10^13^ cm^−2^. This is seen in Figure 33 [178]. 

In a different work, Pandit et al. [169] investigated the structural, electrical, and TE properties of 2D SnTe monolayers using DFT in conjunction with semi-classical Boltzmann transport theory. It was discovered that SOC significantly impacted the electrical structure and characteristics. For n-type SnTe, the maximum expected zT value was 1.46 at 700 K. Ju and Kim [179] synthesized porous SnTe NSs and reported their TE properties. Its α is high and has limited heat conductivity due to flawed geometries and incredibly small nanoparticle sizes. At 923 K, they reported a zT of 1.1. Panwar et al. [180] synthesized SnTe thin films of thickness 55, 33 nm by vacuum deposition method on a glass substrate. They found that it has a higher zT than that of bulk SnTe thin films.

### 4.3. SnSe TE Properties

**Figure 34:**
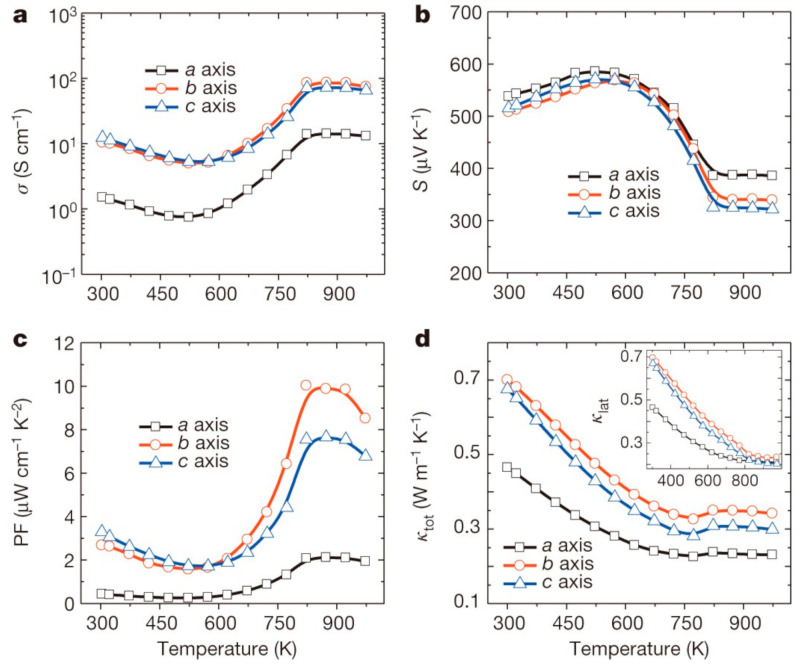
TE characteristics for SnSe crystals with changing temperature: (**a**) total conductivity, (**b**) α, (**c**) power factor, and (**d**) electrical conductivity. Lattice thermal conductivity as a function of temperature, inset. Reproduced from [182] under permissions from copyright clearance center.

Likewise, monolayer SnS and SnSe exhibit TE properties. Aspandiar et al. [181] mentioned that SnSe is the heavier analog of SnS. Additionally, they noted that both SnSe and SnS crystallize in a highly anisotropic layered orthorhombic structure (Pnma) at room temperature, but at high temperatures, they change into Cmcm, which exhibits a greater value of zT. Zhao et al. [182] obtained a zT value of 2.6 at 973 K because of the ultra-low lattice thermal conductivity and moderate power factor throughout the *b*-axis in single crystal SnSe. Figure 34 shows different TE parameters of SnSe with temperature [182]. 

SnSe nanoplates were created by hydrothermal synthesis and spark plasma sintering (SPS), as described by Chandra and Biswas [183]. These nanoplates were subsequently integrated into Ge. A maximum zT of approximately 2.1 was reported at 873 K by anisotropic measurement parallel to the SPS pressing direction. Ge doping up to 3% increased the p-type carrier concentration, enhancing electrical conductivity. Li et al. [184] synthesized porous SnSe NSs by solution synthesis doped with Zn and Ga. Higher carrier concentrations brought about by doping resulted in increased electrical conductivity; they also helped to explain the NSs’ low lattice thermal conductivity. As a result, 0.8 was the average zT, and ~1.86 was the maximum zT that they reported. Wang et al. [185] investigated TE and electric properties of SnSe single sheet by first principle calculations. They realized an intrinsic thermal conductivity of 2.57 W/mK (zigzag) and 2.02 W/mK (armchair) assessed at ambient temperature. These findings demonstrate SnSe’s promise for TE applications. Dong et al. [172] used VASP in conjunction with USPEX to investigate the optical and electrical characteristics of monolayer SnSe. Using USPEX, they discovered a new hexagonal phase known as β′-SnSe. They also mentioned that the most stable phases of SnSe are observed to be energetically close to the β′ phases. They achieved a high TE behavior in the β′-SnSe phases, which have a zT value as high as ~0.93 to 2.51 for SnSe at temperatures ranging from 300K to 900K. Both low elastic constants primarily cause low thermal conductivity due to the high power factor, substantial lattice anharmonicity, and weak inter-Sn bonding strength, which they ascribed to the band convergence close to the Fermi level and the quantum confinement of 2D substances.

**Figure 35:**
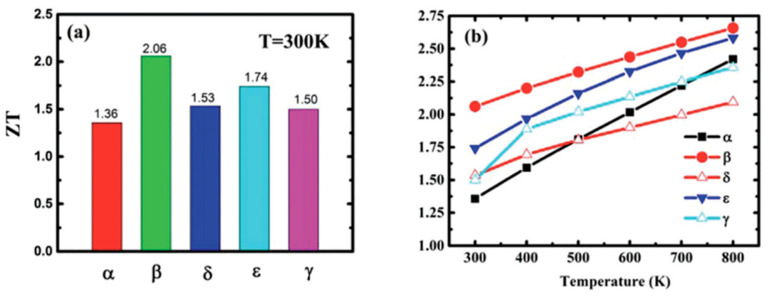
(**a**) At 300 K, the zT values of five monolayer SnSe phases. (**b**) SnSe allotropes’ zT values at different temperatures. Reproduced from [122] under permissions from copyright clearance center.

**Figure 36:**
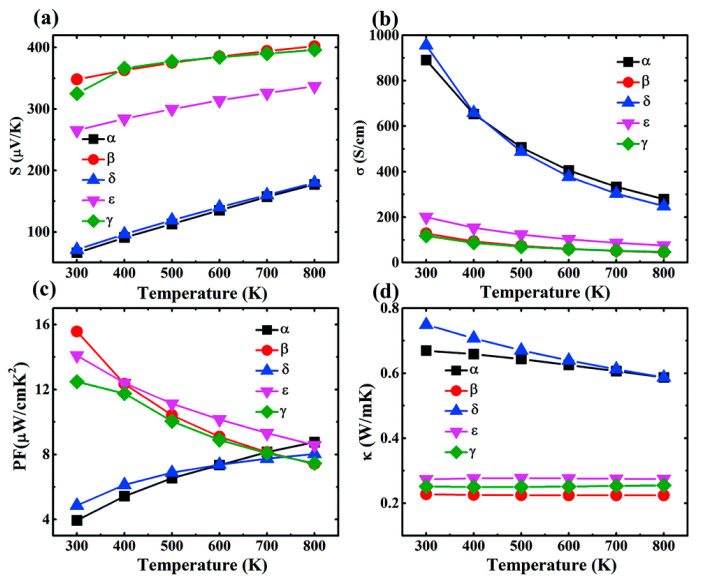
Variations of SnSe allotropes’ (**a**) α, (**b**) electrical conductivity, (**c**) power factor, and (**d**) thermal conductivity at various temperatures. Reproduced from [122] under permissions from copyright clearance center.

Using DFT in conjunction with Boltzmann transport theory, Shafique et al. [168] investigated the TE and phonon transport properties of SnSe. Their findings demonstrated that the compounds’ monolayer TE performance is better than their bulk phases. They discovered that although the electrical and α are high, the computed lattice thermal conductivities are low. Fang et al. [170] found that the SnSe monolayer exhibits an optimum power. Furthermore, they observed that the intrinsic lattice thermal conductivity was low due to the tiny group velocities and significant anharmonic phonon activity. Because of this, SnSe has good thermal equilibrium qualities. At temperatures between 300 and 700 K, the zT for SnSe can reach approximately 0.25 to 1.41. Through the application of Boltzmann transport theory and first-principles electronic structure computations, Ding et al. [186] showed thus, in contrast to bulk SnSe, monolayer SnSe has a higher lattice thermal conductivity, which results in a lower TE figure of merit. Additionally, they discovered that electron doping could lead to improved TE performance and a decrease in the anisotropic level in monolayer SnSe. They discovered that, along a zigzag path, the zT value for monolayer p-type SnSe is 1.2, and for n-type SnSe, it is 2.5. In 2D nanoplates of Ge-doped SnSe produced using a straightforward hydrothermal process succeeded by SPS, Chandra et al. [183] reported a zT value of around 2.1 at 873 K. Using the anisotropic data, they discovered a zT value of around 1.75 at 873 K parallel to the SPS pressing direction. Additionally, they discovered that at 873 K, the p-type carrier concentration in SnSe nanoplates is greatly enhanced by 3 mol% Ge doping. This leads to a high power factor and electrical conductivity of about 5.10 μW/cm-K^2^. Guo and Wang [187] examined the electrical transport of monolayer SnS and SnSe using first-principles calculations, semi-classical Boltzmann transport theory, and the SOC. According to their findings, the electronic, lattice thermal conductivities and power factor anisotropy of SnS and SnSe primarily explain their isotropic TE conversion efficiency. The computed findings indicate little change in zT for SnS and SnSe for different carriers on-and p-type. It is discovered that SnS and SnSe have superior TE performance and higher TE conversion efficiency when doping with p-type SnSe. Hu et al. [122] employed ab initio DFT to predict the TE behavior of the allotropes of monolayer SnSe. Figure 35a shows the zT values of all the allotropes at 300K, while Figure 35b shows the variations of the zT values with temperature for all the allotropes. Among all the allotropes, they found that β−SnSe is the highest among all the SnSe allotropes. They also studied variations of α, electrical conductivity, power factor, and thermal conductivity of SnSe allotropes at different temperatures (see Figure 36). Zhu et al. [188] explored the electronic and phonon transport properties of 2D hexagonal SnSe using the first principle study. Using calculated transport parameters, they obtained a zT value of 2.32 for p-doped SnSe at 700 K.

### 4.4. PbS TE Properties 

**Figure 37:**
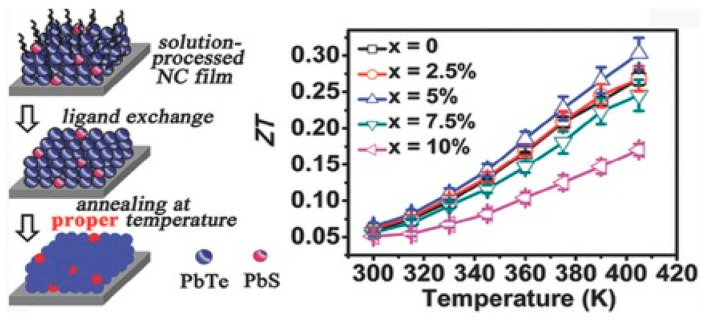
Synthesis of PbS and PbTe nanocrystal films (**left**). Variations of zT value at different temperatures with percent of crystals. Reproduced from [189] under permissions from copyright clearance center.

Using a layer-by-layer technique with ligand exchange, Ding et al. [189] produced PbS and PbTe nanocrystal films on glass substrates while adjusting the concentrations of PbS and PbTe in the samples. Electrical conductivity was improved by necking between nanocrystals, which was made more accessible by ethylenediamine ligand exchange. Because thermal annealing preserved the many surfaces between the nanocrystals, conductivity was further enhanced, but thermal conductivity was decreased. Thus, it led to a higher zT value of 0.30 at 405 K with 5% PbS NC. The values of zT, α, and electrical conductivity with temperature are shown in Figure 37 [189].

**Figure 38:**
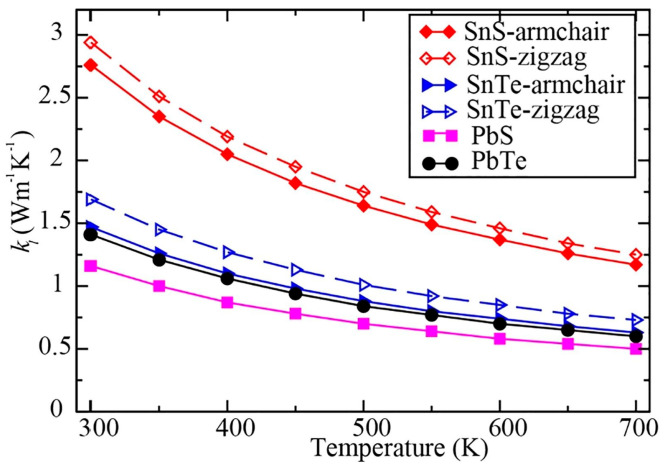
Temperature-dependent lattice thermal conductivity (κl) of SnS, SnTe, PbS, and PbTe MLs. Reproduced from [169] under permissions from copyright clearance center.

Using an ab initio technique, Haq et al. [190] examined the thermoelectric (TE) properties of monolayer PbS. They found that it had a maximum power factor of 6.1 × 10^10^ W/mK^2^ and a zT of around 1.0, much higher than that of bulk PbS. The improvement was ascribed to the monolayer’s strong Seebeck coefficient and high electrical conductivity, which resulted from its narrow bandgap and a substantial density of states (DOS) close to the bandgap edges. Pandit et al. [169] investigated the TE properties of 2D PbS monolayers using semi-classical Boltzmann transport theory with DFT. They discovered that spin-orbit coupling (SOC) affected the electronic structure and resulted in poor thermal conductivity, contributing to a larger zT value. They stated that the low thermal conductivity value displayed for various materials in Figure 38 was the reason for the more excellent zT value.

The anisotropic characteristic of layered materials such as SnS can be the reason for the decreased thermal conductivity of SnS-armchair compared to SnS-zigzag, as shown in the image above. This kind of material’s crystallographic orientation directly affects its thermal conductivity since it can change the atomic arrangement and bonding strength. Comparing the armchair configuration to the zigzag form, the atoms may encounter fewer bonding contacts along the direction of heat flow, which could result in less effective phonon transport. Contrarily, the zigzag arrangement may have greater phonon transmission and stronger bonding, which would lead to higher thermal conductivity. The reason behind SnS’s armchair orientation’s reduced thermal conductivity in comparison to its zigzag counterpart could be attributed to the anisotropy in thermal transfer that is prevalent in 2DMs. Because thermal conductivity and thermoelectric performance are related, decreased thermal conductivity in the armchair design would improve the thermoelectric figure of merit (zT). This is consistent with the background information given on how low thermal conductivity affects higher zT values for thermoelectric materials.

### 4.5. PbSe TE Properties

Tang et al. [136] obtained a maximum zT value of 1.3 for a p-type doped PbSe monolayer. According to their study, the α along the armchair and zigzag directions are approximately 234 and 280 μV/K, respectively, and correspond to the most significant zT at 900 K. These numbers have a strong correlation with the PbSe monolayer’s unique band structure and DOS. Zhu et al. [188] carried out first-principles computations to analyze the electronic and phonon transport features of 2D PbSe. They showed that 2D PbSe has an exceptionally low lattice thermal conductivity of approximately 0.50 W/mK and a significant α of about 1150 μV/K at room temperature. For p-type PbSe, they reported a zT value of 3.95 at 500 K. Yin et al. used DFT [191] to examine the TE characteristics of PbSe that were doped with both p and n. For p-type and n-type ones, they reported room temperature zT values of 0.8 and 0.3, respectively. According to Gayner et al. [192], for both n/p-type PbSe, the ambient temperature zT in PbSe is comparatively low (≤0.1 to 0.3). To achieve the greatest zT value, PbSe is doped with both p and n-type dopants, with their doping level adjusted accordingly. While Al, B, Ga, Cr, or In had been used as electron doping in PbSe, Na, K, Ti, or Ag had been employed as effective hole dopants. The power factor has been enhanced three to eight times by introducing heavy metals as dopants in PbSe, which will help adjust the conduction/valance bands around the Fermi level. 

### 4.6. PbTe TE Properties

**Figure 39:**
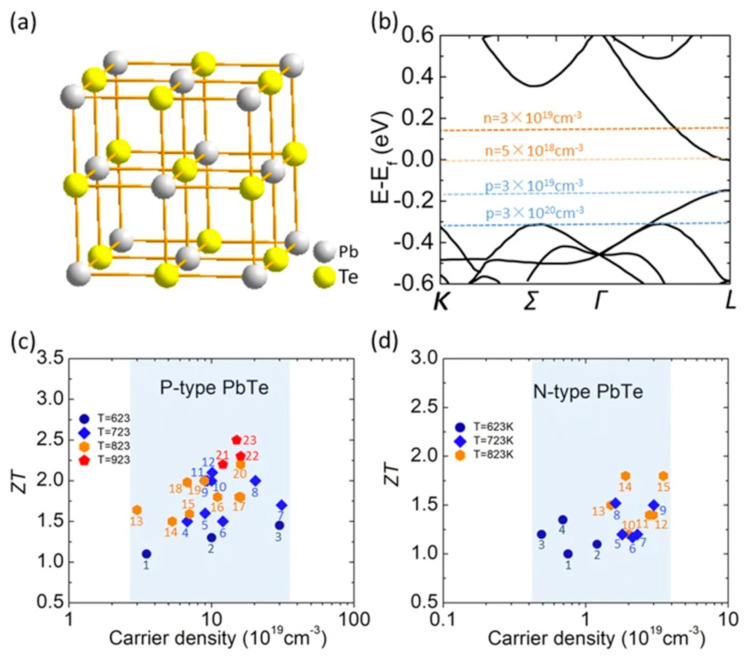
(**a**) Crystal structure, (**b**) Optimized band structure, and zT values for PbTe structures with carrier concentrations of (**c**) P-type and (**d**) N-type PbTe. Reproduced from [193] under permissions from copyright clearance center.

It is recognized that PbTe is a highly potential compound for intermediate-temperature power generation [193]. Investigators doped PbTe as TE materials using both n-and p-type impurities. Most of the work relating to the TE properties of 2D PbTe is theoretical, although some laboratory work uses theoretical predictions. Below, we summarize various works conducted to investigate the properties of TE for 2D PbTe. The zT of p-type PbTe was raised by Pei et al. [194] and Girard et al. [195] using band convergence along with nanostructuring technology. Through band convergence, they achieved a high value of density of states for valance band N_v_ to enlarge the effective mass. They showed that, despite the possibility of a decline in carrier mobility due to inter-valley scattering, the increase in effective mass outweighs the reduction in mobility of the carrier, producing an improved net zT. Distortion of energy levels into the valence bands or conduction can be produced by increasing the α and effective mass [194]. According to Heremans et al. [196], there are two methods to achieve this: (1) adding an excess density of states n(E) close to the Fermi level, and (2) increasing the energy-dependent μ(E), a process referred to as “resonant scattering”. Additionally, they stated that the resonant state can only significantly enhance the α near and below room temperature due to its extreme temperature sensitivity. In p-type PbTe, imperfections in the host material can also raise the α. This will worsen carrier mobility and enhance phonon scattering. Introducing an exotic phase with a modest band offset compared to a matrix, specifically band alignment, is one of the most successful strategies [197]. Conduction band flattening for n-type PbTe can significantly increase the effective mass and maximize the α [198]. Adding group-IIIA elements (Ga, In, and Tl) to PbTe can also raise the impurity levels and the α [199]. The substitution of Ga and In (+3) on Pb (+2) sites does not increase PbTe’s carrier density. The impurity levels that Ga and In create have the potential to act as a charge reservoir by capturing free electrons at low temperatures. The carrier density will rise as the temperature rises because the trapped electrons will be freed from the deep impurity levels and enter the conduction band [200]. Figure 39 shows the optimized band structure and zT values of n-and p-type PbTe structures with carrier concentrations [193]. The figure shows that p-type PbTe demonstrated a higher zT value than n-type, which is ~2.5 at 923 K at ρ = ~1.5 × 10^20^ cm^−3^. 

To maximize PbTe’s TE figure of merit, Hao et al. [201] described the technique of increasing carrier concentration through foreign atom doping and lowering thermal conductivity by introducing nanostructures. Several methods have been documented to raise the zT value of PbTe: nanostructures, alloy concentration, doping adjustments, energy band engineering, synergistic effects, all-scale layered architecture design, etc. Bafekry et al. [202] examined the structural, electrical, optical, and TE properties of monolayer PbTe using first-principles calculations conducted within the context of DFT. They discovered that the band gap shrinks when layer thickness increases from monolayer to bulk. They discovered that at strains larger than +6%, the application of uniaxial strain causes a direct-to-indirect bandgap transition. Tang et al.’s [203] first-principles calculations and semi-classical Boltzmann transport theory were used to examine the electrical transport and TE characteristics of a honeycomb-like puckered PbTe monolayer. According to their findings, the puckered PbTe monolayer combined with the SOC effect is a direct gap semiconductor. They also deduced low lattice thermal conductivity. Table 3 shows different TE properties of Sn/Pb (X = S, Se, Te).

The thermoelectric (TE) properties of SnSe, SnS, SnTe, PbS, PbSe, and PbTe have been discussed in this section. Each of these materials exhibits unique behaviors in terms of TE performance. Thanks to its low heat conductivity and extremely anisotropic crystal structure, SnSe has become a standout material with record-high zT values. Although SnSe and SnS display similar structural characteristics, SnSe’s comparatively higher heat conductivity limits its potential for TE. Doping SnTe to minimize heat conductivity and maximize carrier concentration can improve its thermoelectric performance, which is a result of its rock-salt structure. PbTe is the lead-based chalcogenide that has been investigated the most because of its high zT value, which is related to its advantageous thermal conductivity and tunable electronic properties. Both PbSe and PbS show good TE properties; however, PbSe performs better overall, especially at higher temperatures. While research is still being conducted to improve the electrical and thermal transport properties of these materials through doping, alloying, and nanostructuring in order to increase their zT values for more effective thermoelectric applications, these materials show a variety of TE behaviors overall.

Zhang et al. [174] examined the TE properties of PbTe monolayers. PbTe has ultra-low lattice thermal conductivities. Furthermore, PbTe monolayers with this band structure of the “pudding mold” kind have a high power factor. They concluded that these characteristics result in a high zT for PbTe monolayers, which can exceed 1.58 when the n-type doping concentration is between 10^19^ and 10^20^ cm^−3^. Liu et al. [176] could determine the TE characteristics of SnTe monolayers. Based on the predicted results, PbTe monolayers are shown to be semiconductors with an indirect band gap. Their modest effective masses result in strong electrical conductivities and substantial carrier mobilities. They observed that the lattice thermal conductivity of PbTe is quite low because of the considerable anharmonicity and low phonon group velocity. The optimal zT value of PbTe is found to be 5.91 at 500 K. Pandit et al. [169] investigated the structural, electrical, and TE characteristics of 2D PbTe monolayers using semi-classical Boltzmann transport theory combined with DFT. It was discovered that SOC had a major impact on the electrical structure and characteristics. 

## 5. Optical Properties

### 5.1. SnSe Optical Properties

**Figure 40:**
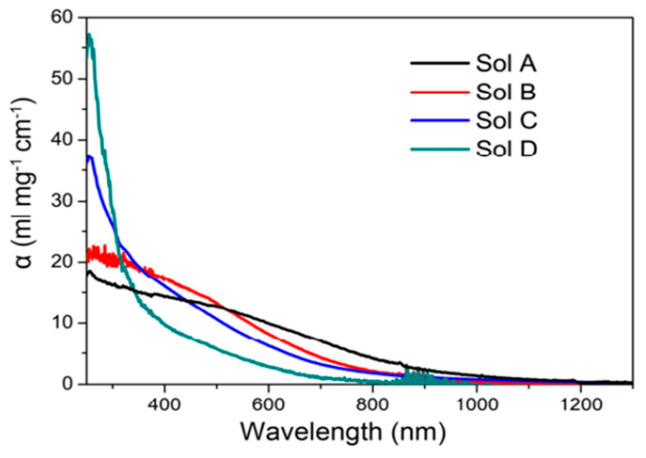
α(ω) of SnSe NSs of different thicknesses. Reproduced from [33] under permissions from copyright clearance center.

**Figure 41:**
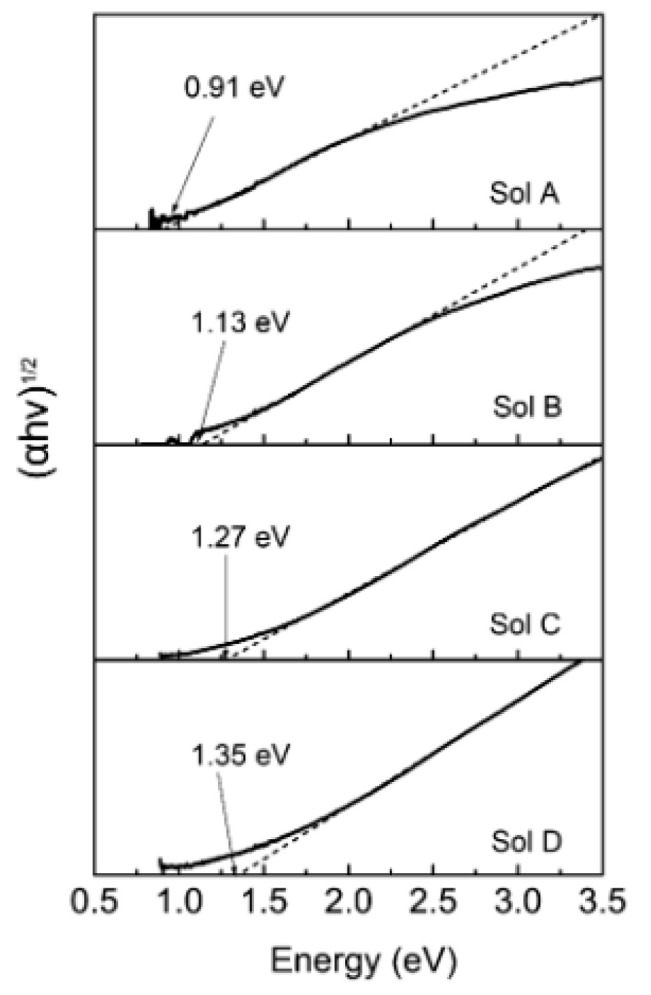
Optical bandgap of SnSe NSs of different thicknesses. Reproduced from [33] under permissions from copyright clearance center.

**Figure 42:**
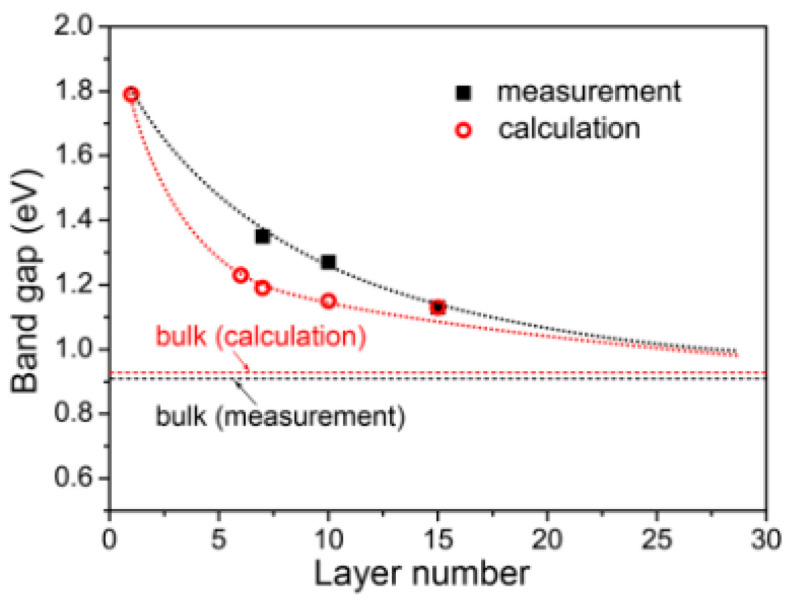
Optical bandgap and the corresponding layer thickness of SnSe NSs of different thicknesses. Reproduced from [33] under permissions from copyright clearance center.

**Figure 43:**
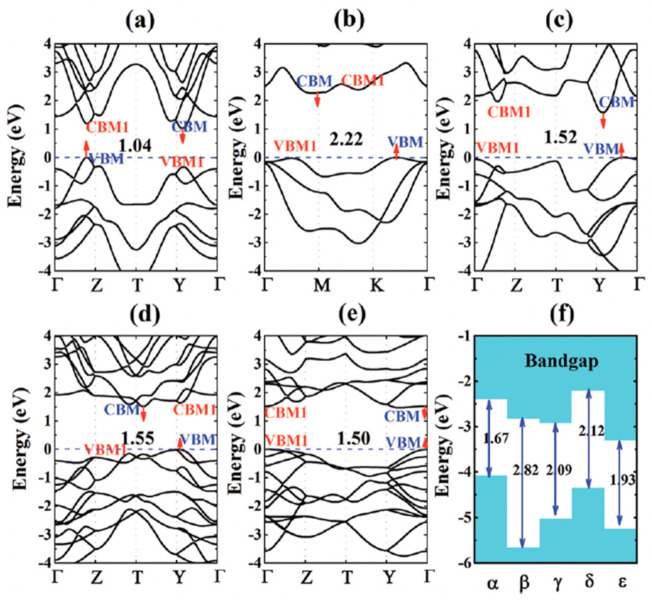
The band structures of five different allotropes based on GGA (**a**) α-SnSe, (**b**) β-SnSe, (**c**) γ-SnSe, (**d**) δ-SnSe, and (**e**) ε-SnSe, (**f**) bandgaps of five allotropes based on HSE06. Reproduced from [122] under permissions from copyright clearance center.

Huang et al. [33] synthesized samples of a few layers of thick SnSe NSs using a typical bath sonication exfoliation method. They prepared four solutions named A, B, C, and D with various thicknesses varying the centrifugation speed. According to their report, the median thickness values for Sol B, Sol C, and Sol D are 8.9, 5.9, and 4.3 nm, respectively. Sample A is the bulk sample, so these median thickness values translate to 15, 10, and 7 layers, respectively. The α(ω) for each of the four samples is plotted against wavelength in Figure 40. As the thickness of the nanosheet decreases, the α(ω) rises in the UV areas and falls in the visible and infrared regions. They reported indirect bandgaps of 4 different samples as 0.91, 1.13, 1.27, and 1.35 eV. The band gaps (indirect) of the solutions determined by Tauc’s equation can be seen in Figure 41, which shows the tunability of SnSe NSs by varying thicknesses [33]. They also determined the optical bandgap and the corresponding layer thickness theoretically and experimentally, as shown in Figure 42. They reported that as the structure shifts from bulk to monolayer, the computed band gap rises sharply from 0.93 to 1.79 eV. Figure 43 displays the band structures of several phases of monolayered α, β, γ, δ, and ε−SnSe, which were determined using the PBE method [122]. To compensate for the PBE functional’s tendency to underestimate materials’ energy band gaps, scientists additionally ran HSE06 computations. 

**Figure 44:**
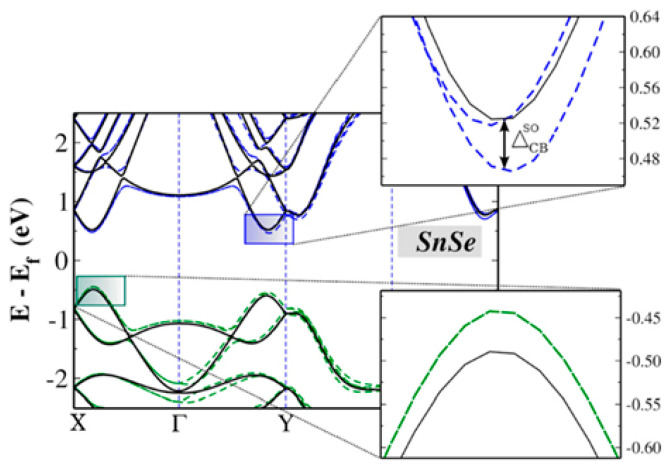
Electronic band structures for SnSe monolayer with and without the SOC effect (continuous black lines) and with green and blue dashed lines. Reproduced from [15] under permissions from copyright clearance center.

For monolayer SnSe, Gomes et al. [15] stated an optical bandgap of 1.41 eV determined by GW using the BSE method. They used the SOC effect to illustrate how the band structure differs, as seen in Figure 44. They concluded that the bands’ spin degeneracy is removed across the Brillouin zone, except for the Γ–X region. All things considered, the SOC barely modifies the band form. The band crossings avoided in the relativistic result, observed at the states at 1.3 eV and 1.4 eV, were the most obvious differences they discovered.

**Figure 45:**
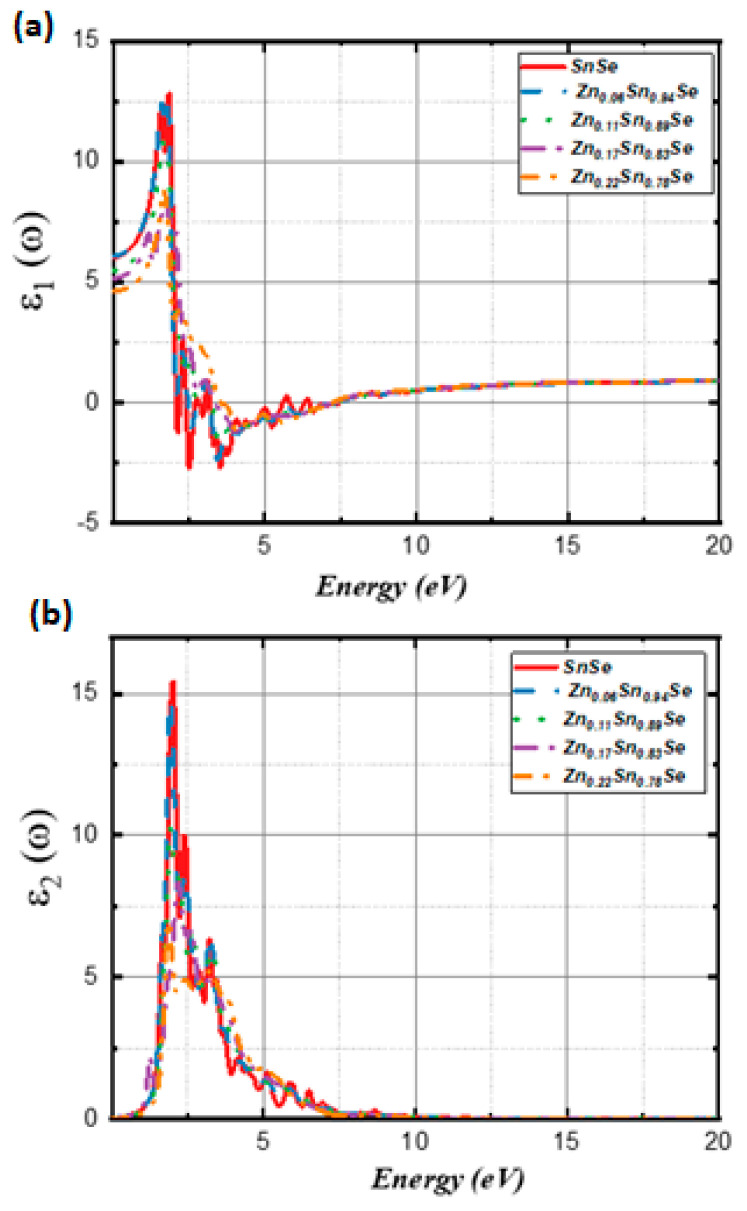
Variations of the (**a**) real and (**b**) imaginary parts of dielectric constants of Zn doped monolayer SnSe with energy. Reproduced from [204] under permissions from copyright clearance center.

However, the bandgap energy of monolayer α-SnSe can vary depending on the type of DFT calculation used. Al Bouzieh et al. [204] reported energy bandgap values of 0.986, 1.067, 1.471, and 1.67 eV for monolayer SnSe determined by GGA, HSE06, and other DFT methods, respectively. They discovered that the conduction bands in SnSe monolayers alter with Zn concentration and that all doped systems behave like semiconductors. The dielectric constant decreased as bulk forms gave way to monolayer forms. They reported that the monolayer system’s static dielectric constant ε_1_(0) is 6.08 for the undoped system. They found that when the ε_1_ value changed to 5.50, 5.15, and 4.62, the Zn concentration increased to 11%, 17%, and 22%, respectively. Figure 45a shows the variations of ε_1_ with energy for doped and undoped systems. 

**Figure 46:**
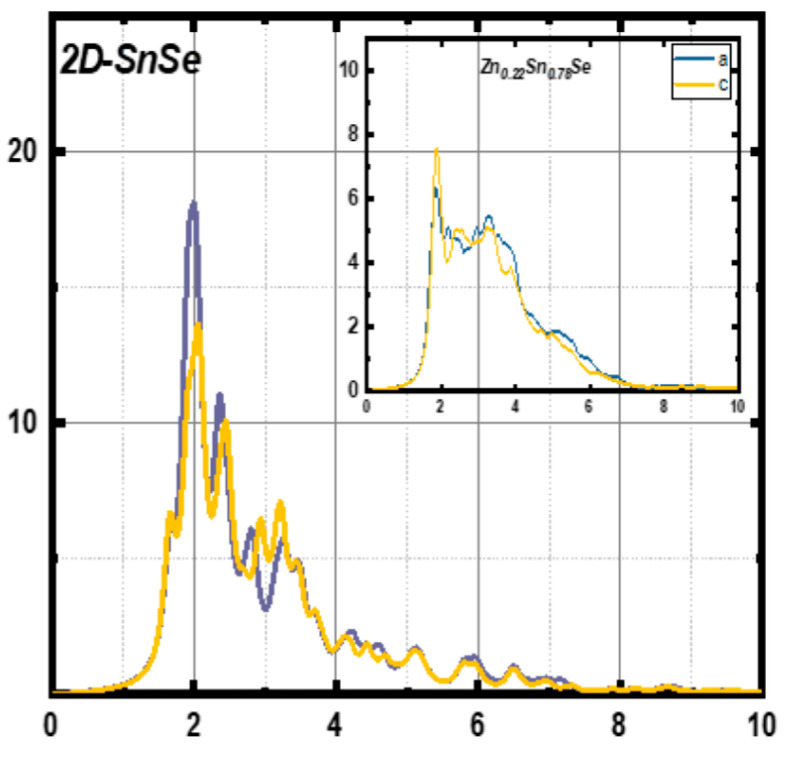
For 2D undoped SnSe and Zn (22%)-doped 2D SnSe, the imaginary component ε_2_(ω) of the dielectric function was calculated along the a, b, and c directions. Reproduced from [204] under permissions from copyright clearance center.

For 2D SnSe systems, ε_2_(ω) is displayed in Figure 45b. As Zn concentration rises, the doped monolayer systems show small, red-shifted peaks. However, as the Zn concentration rises, as Figure 46 illustrates, the peak in the c direction becomes higher.

**Figure 47:**
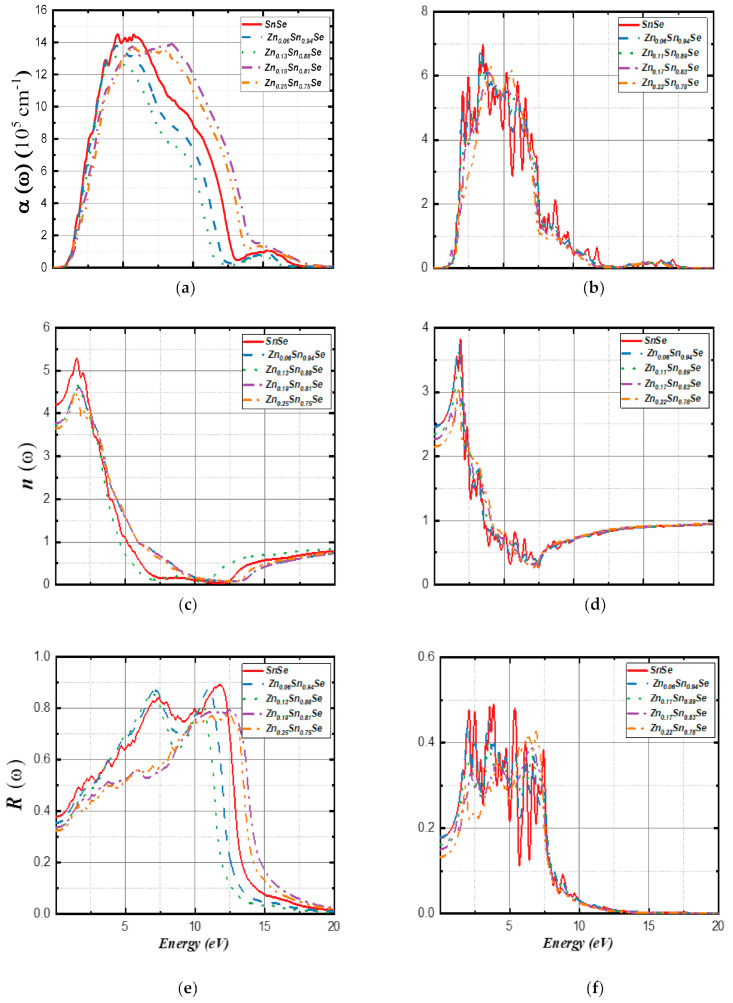
Computed reflectivity R(ω) (**e**,**f**), refractive index n(ω) (**c**,**d**), and α(ω) (**a**,**b**) for 2D and three-dimensional (3D) SnSe compounds (**left** and **right**, respectively). Reproduced from [204] under permissions from copyright clearance center.

They also reported the calculated α(ω) of monolayer SnSe compounds. The spectra of SnSe monolayer compounds exhibited a zigzag pattern, as depicted in Figure 47a,b. The undoped structure and low Zn concentrations, such as 6% and 11%, showed the strongest peak at approximately 3.50 eV. In the case of Zn concentrations of 17% and 22%, the peak shift to ~4.0 eV. Figure 47c,d illustrates the monolayer systems’ refractive index n(ω). The undoped 2D SnSe monolayer was reported to have a value of n(0) of 2.47, which decreased to 2.15 as the concentration of Zn increased. The reflectivity spectrum of R(ω) for an undoped SnSe monolayer with several reflection peaks is displayed in Figure 47e,f. It is also evident from Figure 47e,f that the maxima for the reflection coefficient blue-shift to around 7 eV at Zn concentrations of 17% and 22% as Zn concentration rises.

**Figure 48:**
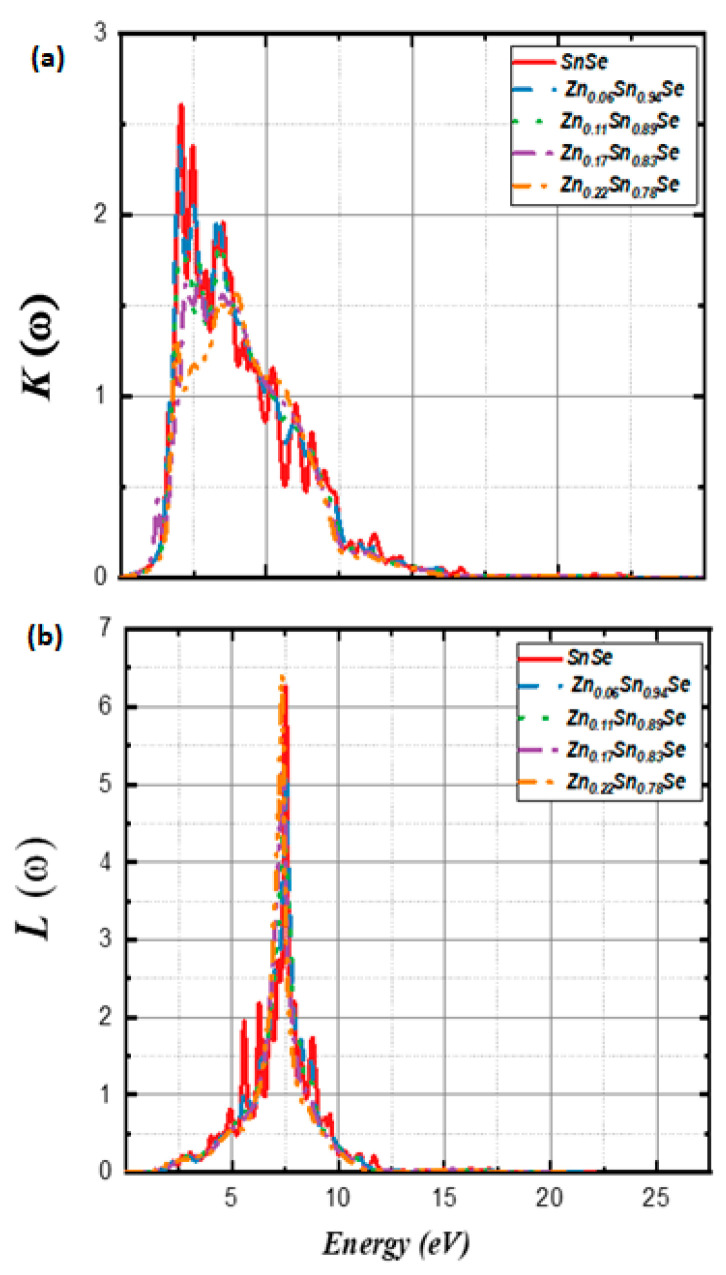
Values of 2D SnSe compounds’ (**a**) extinction coefficient K(ω) and (**b**) energy loss L(ω) were calculated. Reproduced from [204] under permissions from copyright clearance center.

The fluctuations of 2D SnSe’s loss energy L(ω) and extinction coefficient K(ω) are displayed in Figure 48. The peaks of the extinction coefficient trends are sharper in doped systems than in undoped systems. The trends are nearly identical. Zn doping had little effect on the loss energy peaks for 2D SnSe compounds, as Figure 48 illustrates. The L(ω) peaks were reported at ~7.50 eV for all 2D systems.

### 5.2. SnS Optical Properties

**Figure 49:**
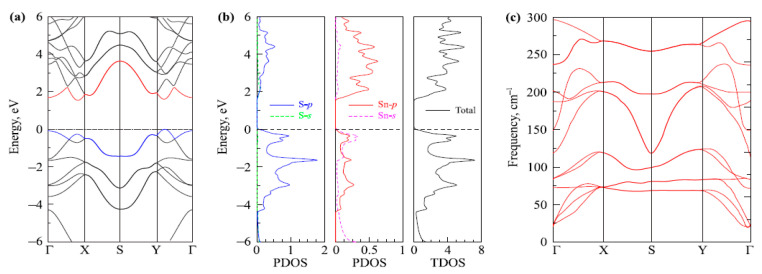
(**a**) Band structure (red line is the conduction band, and the blue line is the valence band), (**b**) density of states, and (**c**) phonon dispersion curves of monolayer SnS at equilibrium. Reproduced from [205] under permissions from copyright clearance center.

**Figure 50:**
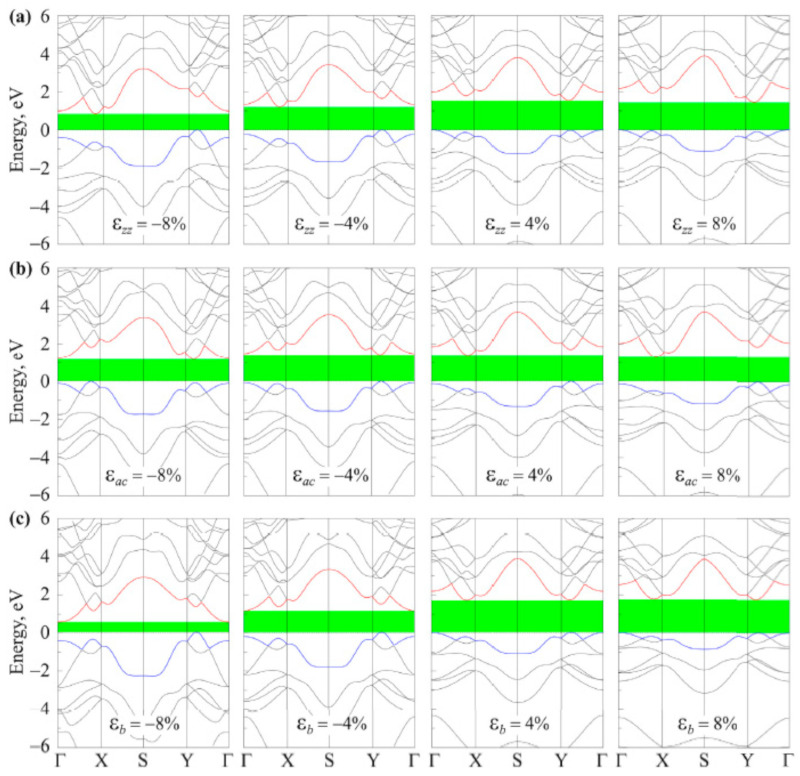
Monolayer SnS band structure with (**a**) ZZ-strain ε_zz_, (**b**) AC-strain ε_ac_, and (**c**) biaxial strain ε_b_. Reproduced from [205] under permissions from copyright clearance center.

**Figure 51:**
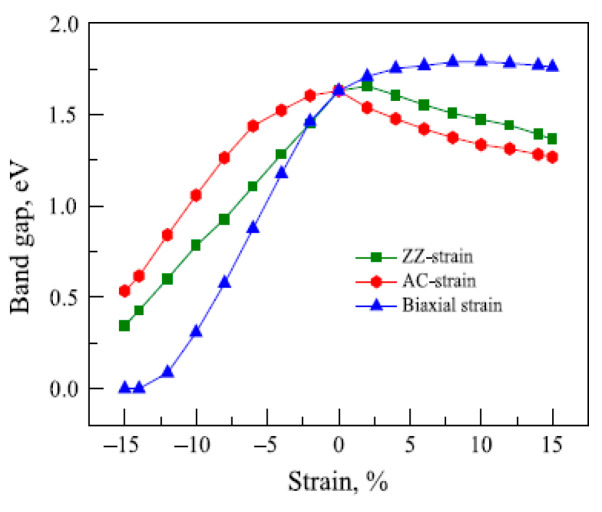
Variation of bandgap of monolayer SnS with strain. Reproduced from [205] under permissions from copyright clearance center.

**Figure 52:**
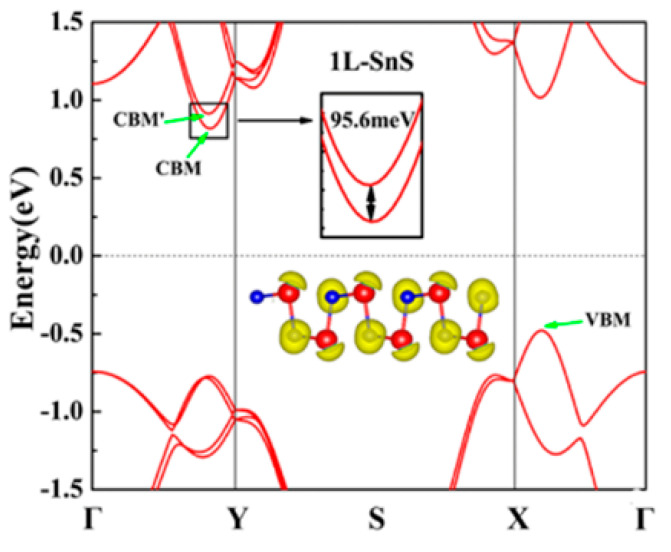
Band structure of monolayer SnS. Reproduced from [166] under permissions from copyright clearance center.

**Figure 53:**
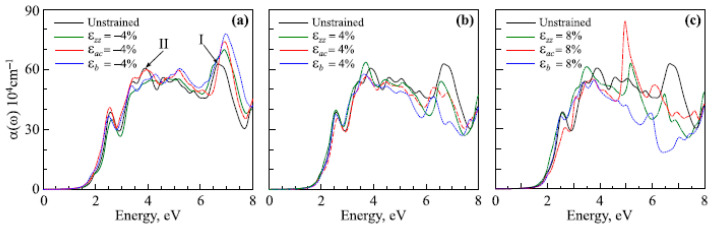
Straitened monolayer SnS α(ω) at elongations of −4% (**a**), 4% (**b**), and (**c**) 8%. Reproduced from [205] under permissions from copyright clearance center.

Khoa et al. [205] used first-principles calculations to demonstrate that the semiconductor-like monolayer SnS at equilibrium has a wide bandgap of 1.63 eV. Figure 49 shows that the energy band structure of monolayer SnS is displayed at equilibrium. They applied tensile strains on the material to see the effect of various properties and found that the effect of strain on the band gap is quite small [205]. Figure 50 [205] depicts how the band structure changes as strain is applied. They found that at −14% elongation, a consistent compressive biaxial strain led to a semiconductor-metal phase transition, dramatically affecting the bandgap of monolayer SnS. Additionally, they noted that the monolayer’s optical spectra are very anisotropic, and the monolayer SnS’s α(ω) tends to grow while that of tensile strain decreases. It is seen in Figure 51. The energy bandgap for an unstrained SnS monolayer is 1.3 eV, according to research by Li et al. [166]. The band structure of Figure 52 is displayed. The absorbance coefficient of monolayer SnS in the presence of uniaxial ε_ac∕zz_ and biaxial ε_b_ strains was also calculated by Khoa et al. [205]. The monolayer SnS absorption spectra are very anisotropic, as Figure 53 illustrates. They showed that in the tension situation, the biaxial strain ε_b_ decreases the α(ω), but in the compression case, it raises α(ω). In the presence of compression strains, the α(ω) of monolayer SnS tends to rise relative to the unstrain condition, but the α(ω) is reduced in the presence of tensile strains. The greatest α(ω) at equilibrium, at 6.65 eV of energy, is 62.9 × 10^4^ cm^−1^ in the energy range of 0 to 8 eV. 

**Figure 54:**
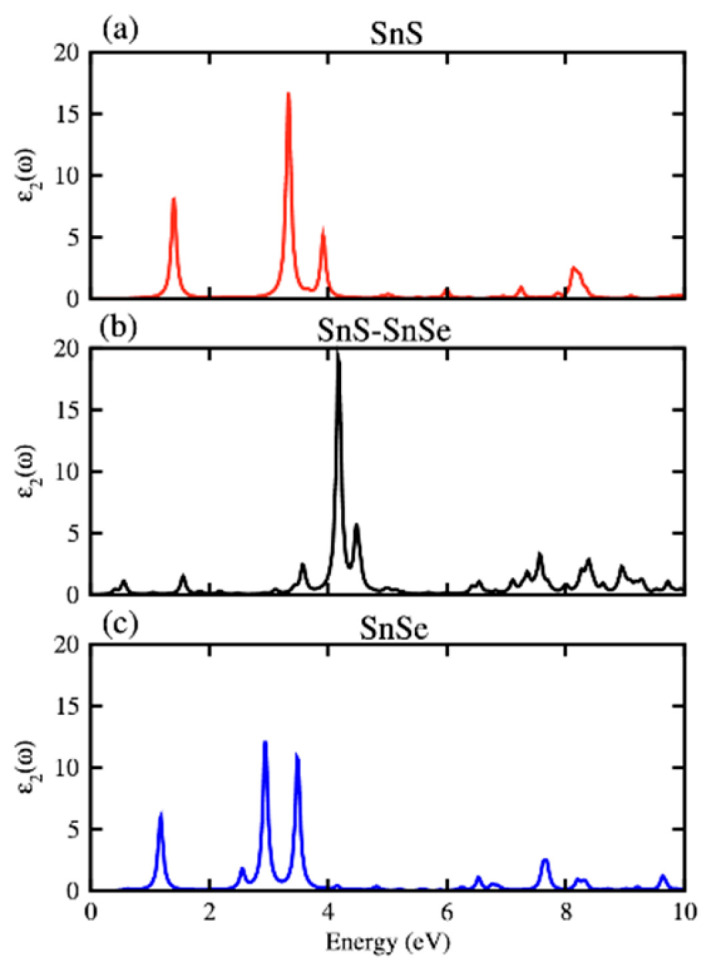
Variation of the imaginary part of dielectric constant with energy for monolayer (**a**) SnS, (**b**) SnS-SnSe, and (**c**) SnSe. Reproduced from [206] under permissions from copyright clearance center.

**Figure 55:**
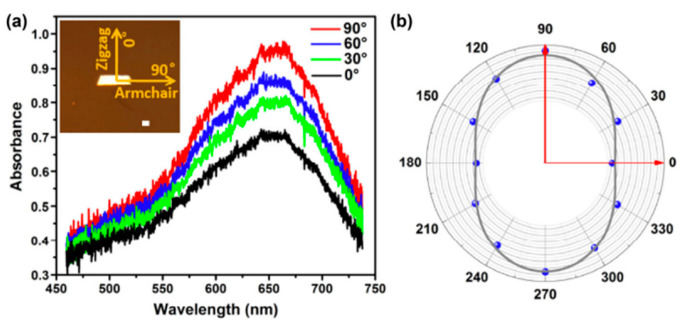
(**a**) Multilayer SnSe flake’s anisotropic absorption spectra at different polarization angles. The SnSe flake’s optical image is seen in the inset. (**b**) The corresponding polar plots are in a plane normal to the flake. The lines represent the least-square fittings, while the symbols represent the experimental values. Reproduced from [206] under permissions from copyright clearance center.

Taher et al. [206] mentioned an energy band gap value of 1.38 eV using the DFT of the SnS monolayer. However, this value is quite low compared to what was reported by Do et al. [206]: 2.89 eV for monolayer SnS determined by first-principles calculation Using UV-vis-NIR spectroscopy, Brent et al. [61] calculated the optical bandgap of SnS nanosheets (NSs), reporting an indirect bandgap of 1.03 eV for SnS NSs in NMP solution based on Tauc’s plot. They pointed out that there might have been a systematic error in this measurement since bigger NSs in the solution probably generated dispersion. They determined that a more precise measurement was obtained because of less scattering when they used fractionated SnS sol B and discovered a greater bandgap of 1.65 eV, which they attributed to quantum confinement phenomena in thinner NSs. Do et al.’s [206] first-principles computations demonstrated the optical characteristics of monolayer SnS. Figure 54 [206] displays the imaginary component of the dielectric constant for monolayer SnS, SnSe, and SnS-SnSe. At about 3.75 eV, it is evident that the monolayer SnS has a strong absorption. Additionally, they examined multilayer SnSe’s (thickness ~200 nm) angle-resolved optical absorption spectra at 450–750 nm wavelengths on a quartz substrate. The inset of Figure 55 displays the angle-dependent optical absorption spectra of the apparatus under study. Anisotropic light absorption is observed in the multilayer SnSe, wherein light polarized in the armchair direction (90°) absorbs more light than light polarized at 0° (zigzag direction). Along the YZ, the optical α(ω) plane and parallel to the *X*-axis direction exhibited anisotropic characteristics due to the orthorhombic lattice of the SnSe doping system, as shown in Figure 55 [206]. Additionally, they observed that there were notable redshifts in the doping systems’ optical absorption edges in comparison to the pure SnSe system, which was linked to a narrowing of the band gap. Their results showed that light absorption was widened by doping. Doping proved advantageous for enhancing optical absorption, as seen by the absorption strengths for the doping cases remaining within the range of 10^5^, similar to that of pure SnSe.

### 5.3. SnTe Optical Properties

**Figure 56:**
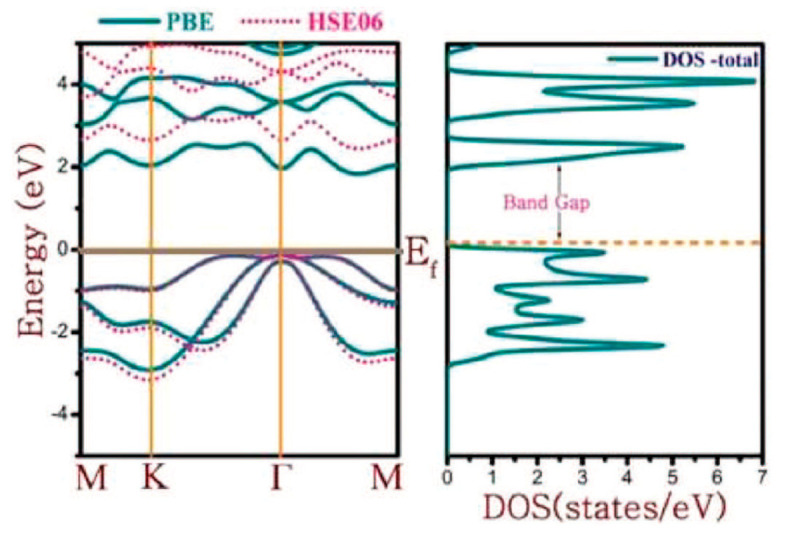
(**Left**) SnTe band structures derived from the HSE06 approximation (red lines) and PBE theory (green lines). (**Right**) The density of states is computed with the GGA PBE approximation for SnTe. Reproduced from [207] under permissions from copyright clearance center.

Fatahi et al. [207] used an ab initio approach to investigate the optical properties of a hexagonal SnTe monolayer. Their findings show that a 2D SnTe monolayer shows a quasi-direct medium band gap of ~2.00 eV and ~2.60 eV (as calculated from HSE06). The band structure of monolayer SnSe is shown in Figure 56 [207]. 

**Figure 57:**
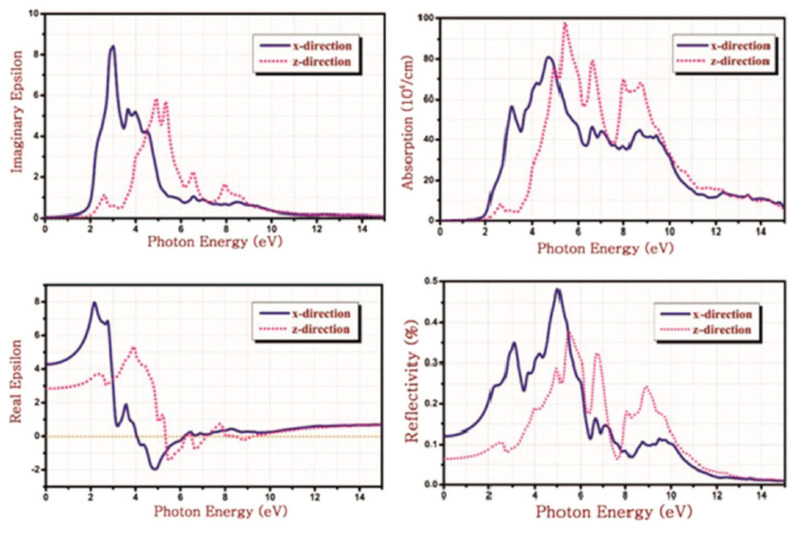
The complex dielectric function’s real and imaginary components, as well as the 2D SnTe monolayer’s absorbance and reflectivity. Reproduced from [207] under permissions from copyright clearance center.

Additionally, they calculated the SnTe monolayer’s reflectivity, α(ω), and imaginary and real parts of the dielectric function using first-principles calculations, which are displayed in Figure 57 [207]. SnTe monolayers in the visible and low-energy portions of the electromagnetic spectrum show nearly little reflection or absorption. Nonetheless, it shows significant reflection and high absorption in the ultraviolet (UV) spectrum.

**Figure 58:**
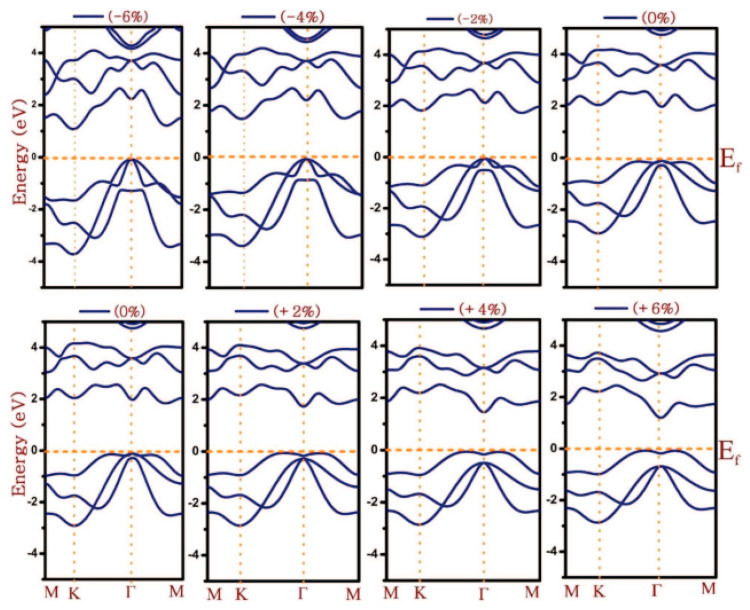
GGA PBE approximation-calculated band structures of all SnTe strain states. Reproduced from [207] under permissions from copyright clearance center.

Figure 58 [207] illustrates the band structures under various strain scenarios for SnTe. The figure indicates that the bandgap decreases as the strain value increases under both tensile and compressive strain situations. 

**Figure 59:**
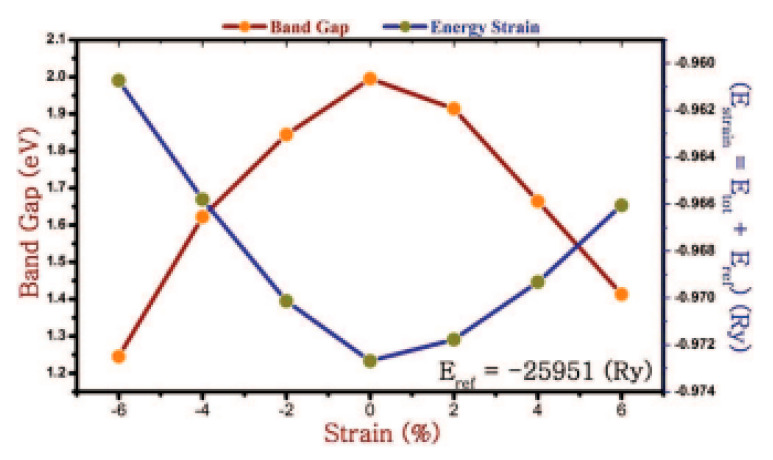
Changes in the energy strain and strain energy gap for SnTe monolayer. Reproduced from [207] under permissions from copyright clearance center.

**Figure 60:**
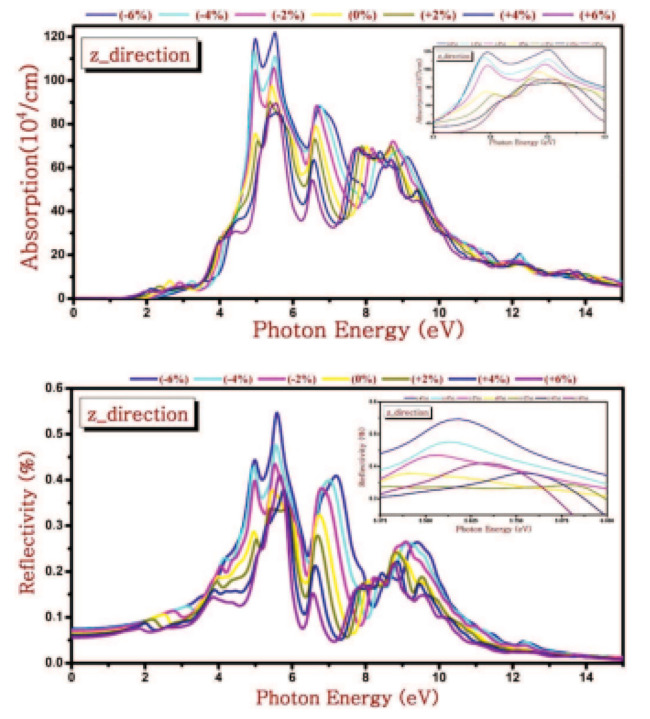
SnTe monolayer variation in absorbance and reflectivity across all strain states. Reproduced from [207] under permissions from copyright clearance center.

They also plotted the variations of bandgap energy and strain energy (E_s_ = (E − E_0_)/n) of the monolayer, which is shown in Figure 59 [207]. Figure 60 [207] displays the monolayer SnTe’s absorbance and reflectivity. These graphs demonstrate that while the absorbance and reflectivity coefficients marginally drop, the strain value increases under compressive and tensile strain circumstances. 

Xu et al. [208] reported the imaginary portion of the macroscopic dielectric function for incident light that was linearly polarized along the x and y directions. When the electron-hole Coulomb interaction is considered, the optical spectra undergo a complete reshaping, indicating the excitonic states’ importance and dominance over the optical properties. They came to the conclusion that the quasiparticle band structure’s direct bandgap corresponds to the GW-BSE method’s restriction to direct band excitation.

### 5.4. PbS Optical Properties 

**Figure 61:**
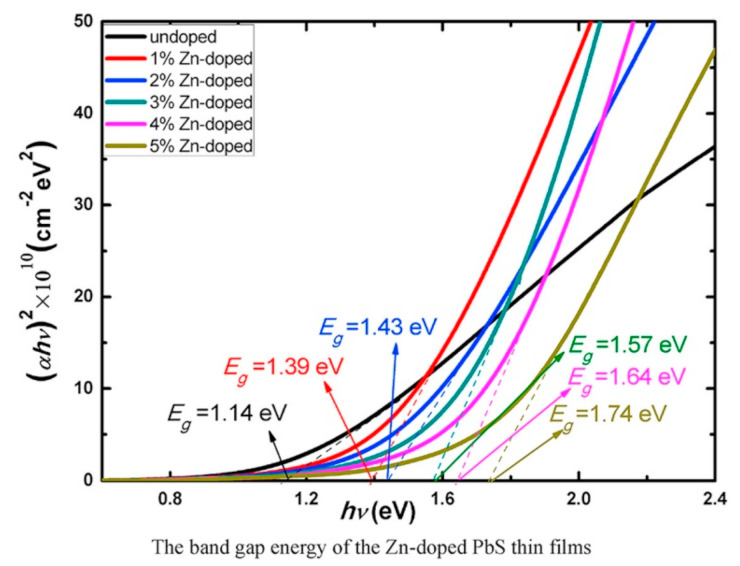
Optical bandgap of PbS nanosheet with for undoped and doped with Zn. Reproduced from [209] under permissions from copyright clearance center.

Liu et al. [209] synthesized Zn-doped PbS NSs on glass substrates by a CBD method. They fabricated different PbS nanosheet samples based on Zn concentration, reported their transmittance, and reported their bandgap (see Figure 61). For undoped, 1%, 2%, 3%, 4%, and 5% Zn doped PbS NSs, they reported its direct bandgap to be 1.14, 1.39, 1.43, 1.57, 1.64, 1.74 eV, respectively as seen from Figure 61 [209] using Tauc equation. 

**Figure 62:**
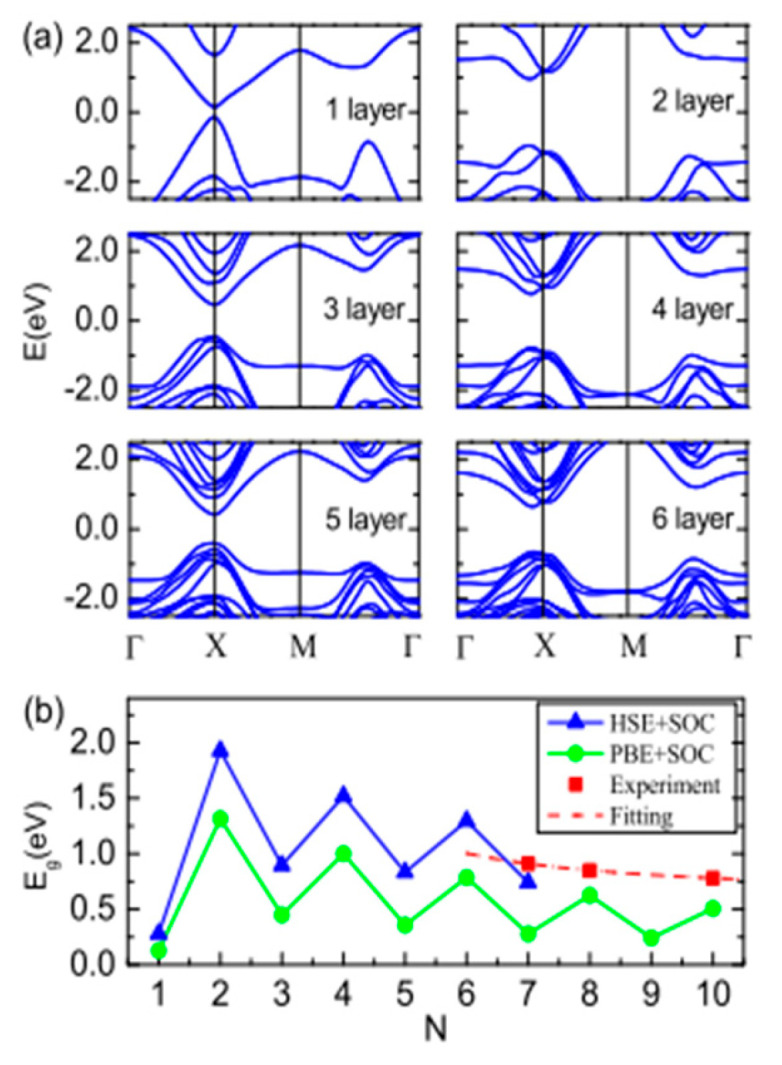
(**a**) Band structure and (**b**) optical bandgap of PbS nanosheet with for undoped and doped with Zn. Reproduced from [133] under permissions from copyright clearance center.

**Figure 63:**
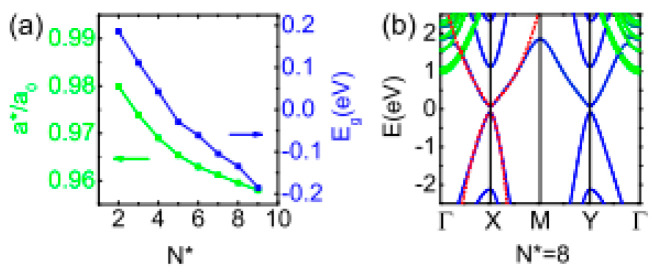
The band structures and lattice constants for the comparable KF-PbS monolayer-KF are shown in (**a**) as a function of the KF layer number (N*), and the band gap (E_g_) and lattice constant (a*) are presented. The flat freestanding monolayer’s lattice constant is a_0_. (**b**) The band configuration where N = 8. The contributions from PbS and KF are denoted in blue and green. The fitted bands using the effective k · p Hamiltonian are represented by the red lines. Reproduced from [210] under permissions from copyright clearance center.

According to Figure 62a, Wan et al. [133] calculated the band structures of PbS (001) in a few layers using the HSE + SOC method, ranging from monolayer to hex-layer. The energy band gap fluctuations with layers of various techniques are depicted in Figure 62b. The band gaps found using the HSE + SOC approach align with experimental values. They noticed that as the number of layers increases, the direct band gap progressively rises to the bulk value and exhibits a pronounced oscillation between odd and even layers. According to their calculations, even layers have higher band gaps because of stronger interlayer orbital hybridization caused by the comparatively lower interlayer distances inside bilayer blocks. Furthermore, they discovered that no band inversion is present in any of these few layers. As shown in Figure 62, the few-layer band gaps range from 0.24 to 1.92 eV, spanning a broad range from the visible to the infrared. In contrast, the band gap of the monolayer PbS is significantly less. They claim that there are two causes behind this. Firstly, the monolayer has a significant crystal field effect, and the bulk crystal possesses cubic symmetries. Second, the fact that PbS’s band gap can be lowered by pressure is likewise compatible with the narrow band gap. After a thorough analysis, they suggested a better structure based on these results: a monolayer sandwiched between two KF layers. To demonstrate the band structure of KF-PbS monolayer-KF, they used the PBE + SOC approach rather than the exorbitant computing cost of the HSE + SOC calculations. They discovered that the strain on the PbS monolayer is less than 4.5% and manageable in the synthesis using the new method. They concluded that the PbS monolayer mostly contributes to the electronic states close to the Fermi level, whereas the KF layers’ contribution is insignificant due to their huge band gaps. Figure 63a,b displays the outcomes.

### 5.5. PbSe Optical Properties

**Figure 64:**
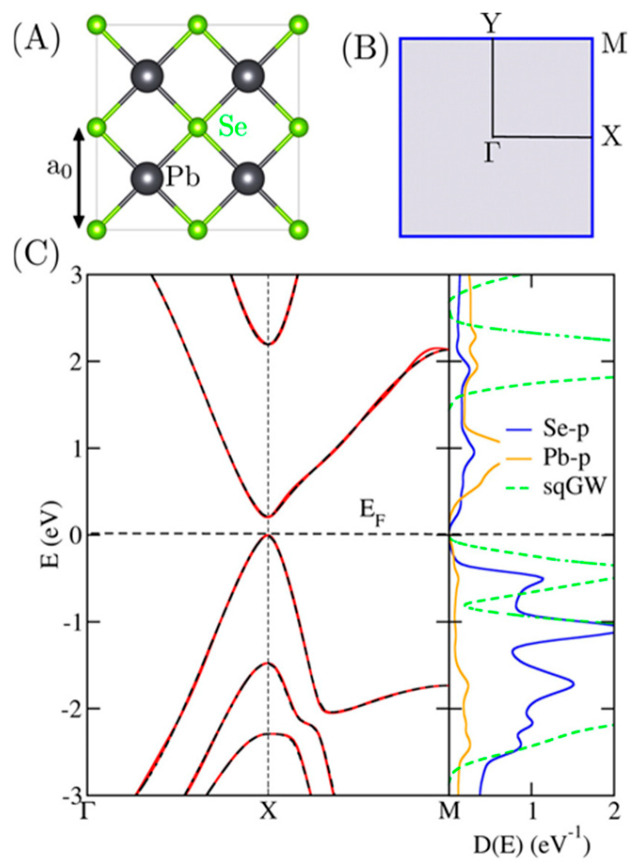
(**A**) The atomic structure’s top view; (**B**) the monolayer PbSe’s initial Brillouin zone along the [001] direction. (**C**) For the pure monolayer of PbSe, the DFT band structure (black dashed bands) is replicated by the downfolded Hamiltonian H_0_ (solid red bands). Reproduced from [210] under permissions from copyright clearance center.

**Figure 65:**
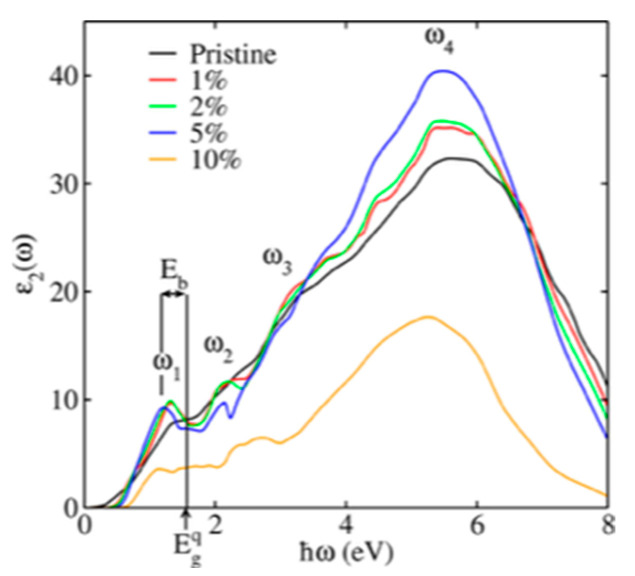
Variations in the dielectric function ε_2_(ω) at different energies for different concentrations of Se vacancies. Reproduced from [210] under permissions from copyright clearance center.

Ekuma [210] investigated the effects of defects on the electrical and optical properties of monolayer PbSe using DFT calculations in conjunction with a typical medium approximation based on fundamental principles. Figure 64B depicts the first Brillouin zone of monolayer PbSe along the [001] direction, while Figure 64A depicts the top view of the atomic structure. As seen in Figure 64C, they expected a direct band gap of 0.21 eV at the specified X point of the high-symmetry zones of the Brillouin zone. The imaginary component of the dielectric function ε_2_(ω) for different concentrations of Se vacancies is displayed in Figure 65. As the concentration of Se vacancies rises, the electron-hole delocalization first increases and subsequently declines. They suggest that the band edge is related to the first peak in the absorption spectra, which is positioned at ω_1_ = 1.30 eV. Such spectral weight redistribution may cause an increase in Se vacancy concentration. Figure 65 shows that when the concentration of Se vacancies rises, ω_1_ redshifts broadens and consistently decreases to around 1.0 at δ = 10%. The broad shoulder seen at ω_3_ indicates the absorption spectra, which occur at a resonant frequency of ω_2_. At ω_4_ ≈ 5.4 eV, ε_2_(ω) reaches its maximum value. Ekuma pointed out that the absorption spectra of the bulk crystal and the pristine monolayer exhibit a similar quality.

**Figure 66:**
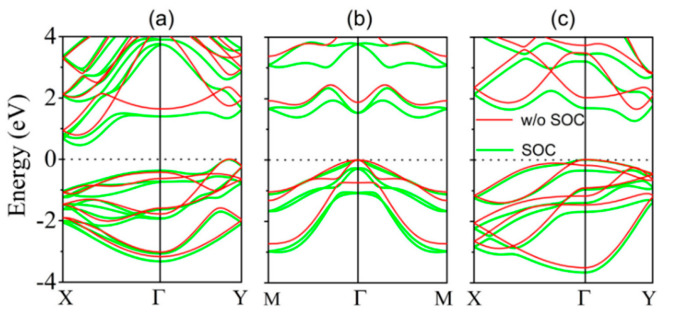
Band structure of (**a**) α, (**b**) β, and (**c**) γ-PbSe monolayers with and without SOC. Red lines represent without SOC, and green lines represent with SOC. Reproduced from [190] under permissions from copyright clearance center.

Nevertheless, they discovered that the resonant characteristics (ω_1_ and ω_2_) below 2.50 eV are only a shoulder in the bulk structure at lower energies, at 1.90 eV. Ekuma concluded that the discrepancies may be ascribed to a variety of factors, including their present data [210]. Haq et al. [190] predicted the optical characteristics of α-, β-, and γ-PbSe monolayers using first-principles calculations within DFT. They employed a DFT method. They carried out the calculations using the cutting-edge WIEN2k computing program. In addition to PBE-GGA, they used the Tran–Blaha modified Becke–Johnson exchange potential to calculate optical characteristics and electronic structures. They discovered that whereas the optical spectra of the α-and γ-types of PbSe monolayers show a notable degree of anisotropy, the β-PbSe showed isotropic electronic structures and spectra (refer to Figure 66). It should be mentioned that their estimates of the band gaps for PbSe monolayers differ from those of Ekuma [210]. They concluded that the observed discrepancy might be caused by the PbSe monolayer’s different crystal symmetry (tetragonal symmetry, which is examined in Reference [210]).

**Figure 67:**
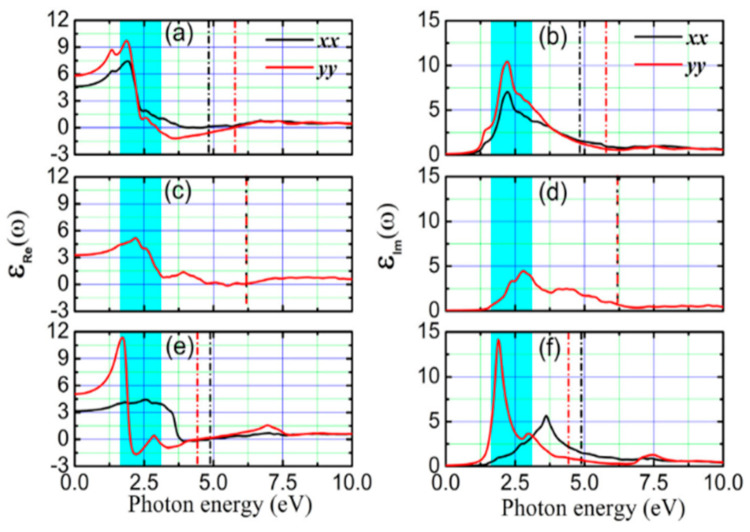
The real and imaginary parts of the dielectric functions of α-PbSe (**a**,**b**), β (**c**,**d**), and γ-PbSe (**e**,**f**) that were computed using the TB-mBJ + SOC along the *x*- and *y*-axes. The band with blue coloration represents the visible range of the solar spectrum. The depicted vertical lines match the excitations of plasmons. Reproduced from [190] under permissions from copyright clearance center.

These monolayers are transparent, as evidenced by their refraction spectra, which span the ultraviolet to NIR spectrum. Additionally, they exhibit a significant optical absorption of the incident light and a notably low reflectance. These characteristics support the efficient use of these new PbSe monolayers in innovative optoelectronic and photovoltaic systems. While ε_R_(ω) of β-PbSe had isotropic behavior, the x-and y-components of the real part of the dielectric function ε_R_(ω) for α-PbSe and γ-PbSe showed notable anisotropy (Figure 67). The highest amplitude found in the ε_R_(ω) spectrum of γ-PbSe is 4.49 at 2.60 eV on the *x*-axis and 11.29 at 1.73 eV on the *y*-axis. Upon reaching its maximum amplitude, ε_R_(ω) experiences a sharp decline before taking on negative values as energy levels rise even more. For the α-and γ-PbSe, larger values of static dielectric constants have also been observed in the y-direction compared to the x-direction. Additionally, the static dielectric constant values of α-PbSe were higher than those of β-and γ-PbSe. The lower band gaps of α-PbSe compared to β-and γ-PbSe are probably the reason for the higher values of its static dielectric constants. 

**Figure 68:**
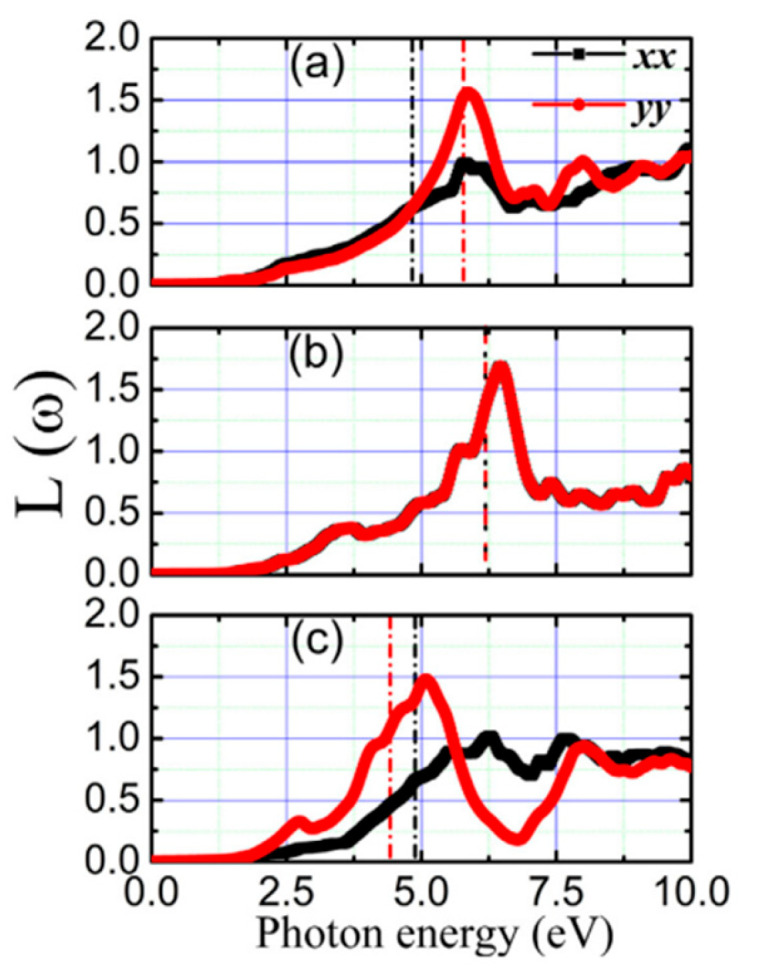
The energy loss function L(ω) was calculated for the α-PbSe (**a**), β-PbSe (**b**), and γ-PbSe (**c**) using the TB-mB + Soc. Reproduced from [190] under permissions from copyright clearance center.

Figure 68 shows variations of the energy loss function (L(ω)) at different energy levels for PbSe monolayers [190]. As this Figure illustrates, the plasmon excitations for α-PbSe have been identified at 4.83 eV and 5.78 eV along the x-and y-directions. They stated that plasmon excitations were discovered at 6.19 eV from the dispersion of ε_I_(ω) and L(ω) in the case of β-PbSe. Below 6.19 eV, single-particle interband optical transitions occur. For β-PbSe, the spectrum of ε_I_(ω) is found to have a broad peak with maximum amplitude at 2.74 eV and a shoulder at 2.39 eV. The isotropic lattice parameters along the x-and y-directions resulted in the isotropic behavior of the ε_R_(ω), ε_I_(ω), and L(ω) determined for β-PbSe. Similar to α-PbSe, the optical characteristics of γ-PbSe showed significant anisotropy in both the x and y directions. In the γ-PbSe example, the plasmon excitations happened at 4.88 and 4.42 eV along the x-and y-directions, respectively. In the instance of γ-PbSe, the single-particle interband transitions have been detected at 1.91 eV and 3.62 eV along the x-and y-directions, respectively.

**Figure 69:**
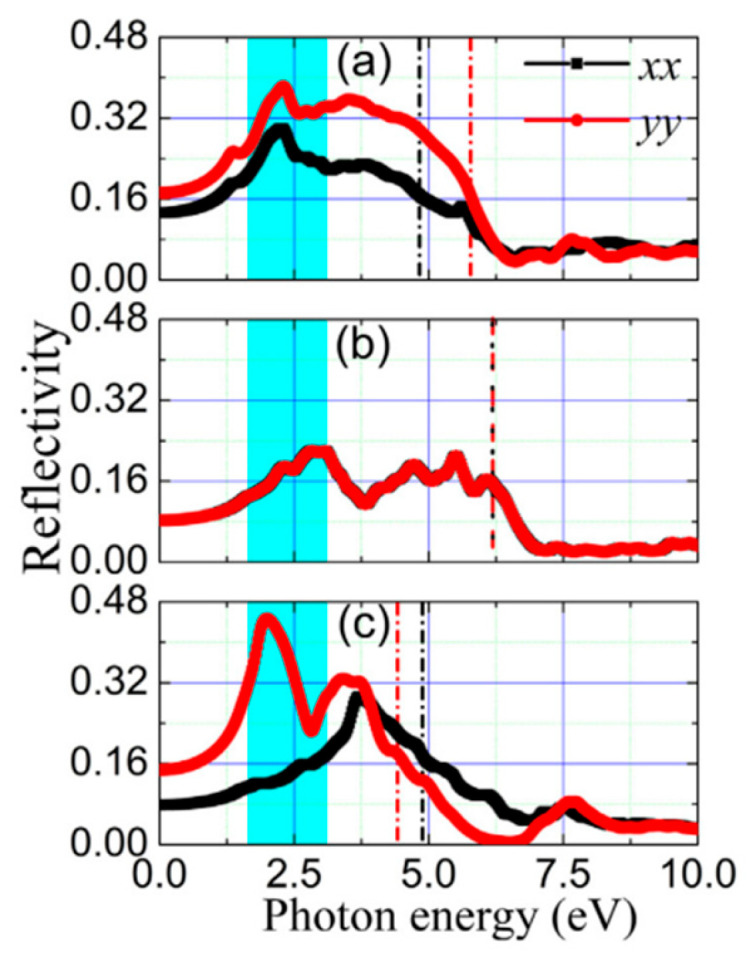
The TB-mB + SOc was used to determine the reflectivity spectra along the *x*- and *y*-axis for the α-PbSe (**a**), β-PbSe (**b**), and γ-PbSe (**c**). The band with a cranium color represents the visible range of the solar spectrum. The vertical lines that represent plasmon excitations have been drawn. Reproduced from [190] under permissions from copyright clearance center.

**Figure 70:**
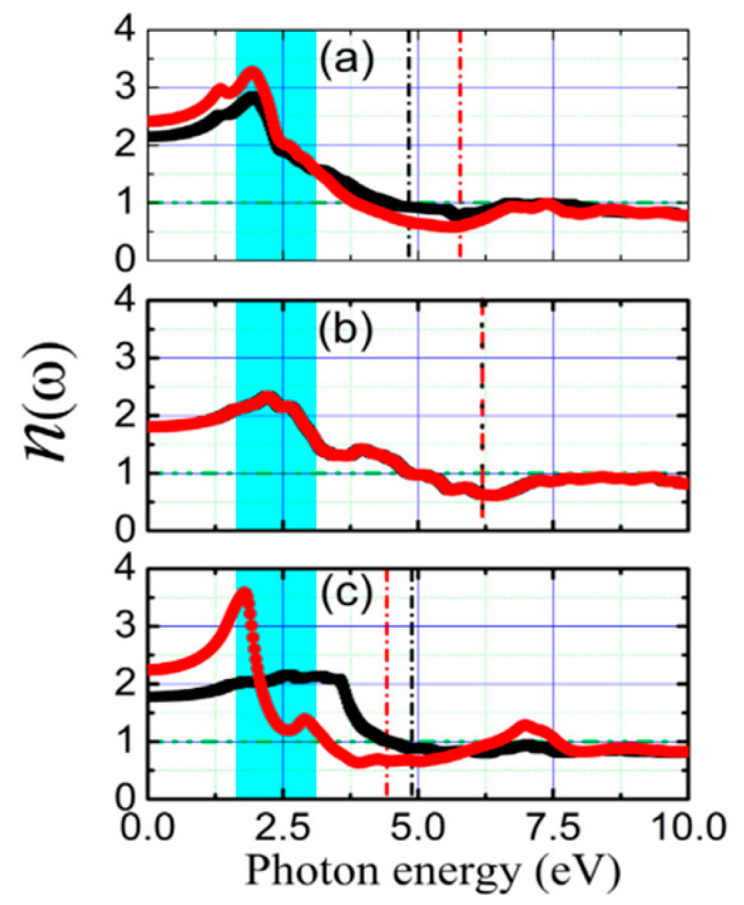
The TB-mB + SOc was used to determine the refraction spectra along the *x*- and *y*-axes for the α-PbSe (**a**), β-PbSe (**b**), and γ-PbSe (**c**). The band with a cranium color represents the visible range of the solar spectrum. The vertical lines that represent plasmon excitations have been drawn. Reproduced from [190] under permissions from copyright clearance center.

They also reported that the reflectivity spectra varied with the photon energies. While α-and γ-PbSe displayed an anisotropic reflectivity dispersion, Figure 69 illustrates the isotropic dispersion of optical reflection for PbSe [190]. In the reflectivity spectrum of the α-and γ-PbSe, it is discovered that the y component is greater than the x component. Furthermore, they stated that compared to β-PbSe, these two monolayers exhibited noticeably greater reflection. In contrast, it is measured at 22% for β-PbSe. Comparably, Figure 69 illustrates how the maximum reflection for the γ-PbSe approaches 44.67% in the visible range and 29.18% along the *x*-axis in the UV [190]. The refraction spectra of the three different PbSe monolayer types are displayed in Figure 70. It is shown that the PbSe monolayers’ n(ω) values are greater than unity over a wide range of the solar spectrum, including the visible, infrared, and lower UV regions. This suggests that the PbSe monolayers are transparent within the specified solar spectrum ranges. While the β-PbSe monolayer behaved isotropically, the n(ω) of the α-and γ-PbSe monolayers displayed significant anisotropy [190]. The α-PbSe refractive indices approach unity at 4.45 eV in the x direction and 3.85 in the y direction. For β-PbSe, the optical refraction is greater than unity up to 4.86 and decreases to less than unity as the energy increases. Comparably, measurements of the optical refraction’s x-and y components have been made up to 4.48 eV and 3.31 eV, respectively, bigger than unity.

**Figure 71:**
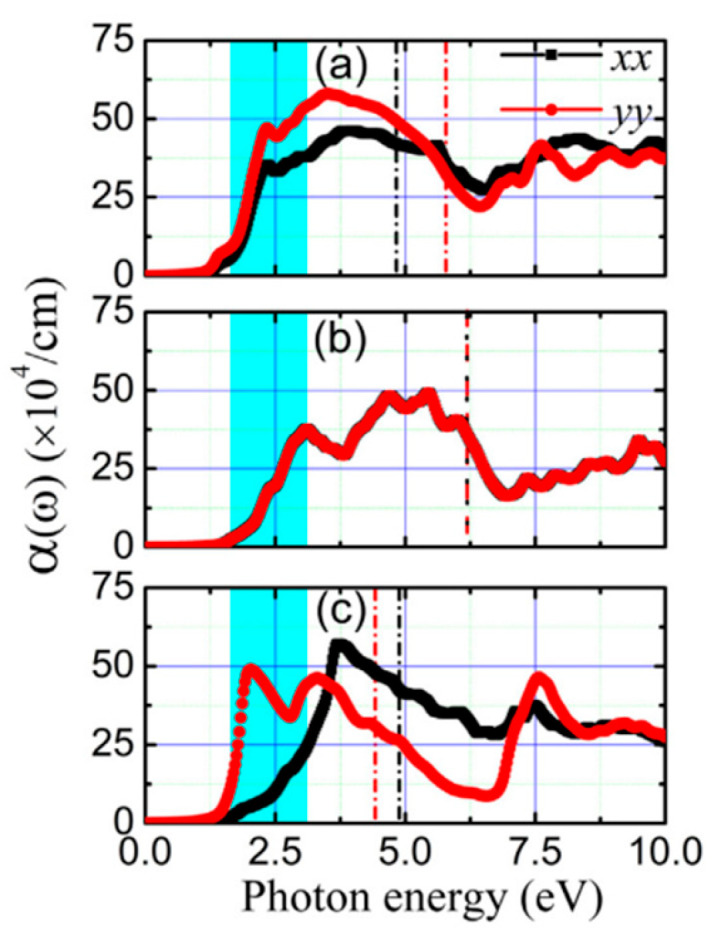
The TB-mB + SOc was used to determine the absorption spectra along the *x*- and *y*- axes for the α-PbSe (**a**), β-PbSe (**b**), and γ-PbSe (**c**). The band with a cranium color represents the visible range of the solar spectrum. The vertical lines that represent plasmon excitations have been drawn. Reproduced from [190] under permissions from copyright clearance center.

The absorption spectra of the three different kinds of PbSe monolayers are shown in Figure 71. The isotropic β-PbSe absorption spectra reach a maximum amplitude. Conversely, the γ-PbSe absorption spectra exhibit strong anisotropy in both the x and y directions, peaking at 3.72 eV for 57.47 × 10^4^ cm^−1^ and 2.04 eV for 48.71 × 10^4^ cm^−1^ at 3.72 eV. Figure 69, Figure 70 and Figure 71 show that for all three polymorphs, the refraction and reflection of incident light decreased in the UV region (usually exceeding 6 eV) and that these monolayers primarily absorb high photon energy incident light.

### 5.6. PbTe Optical Properties

**Figure 72:**
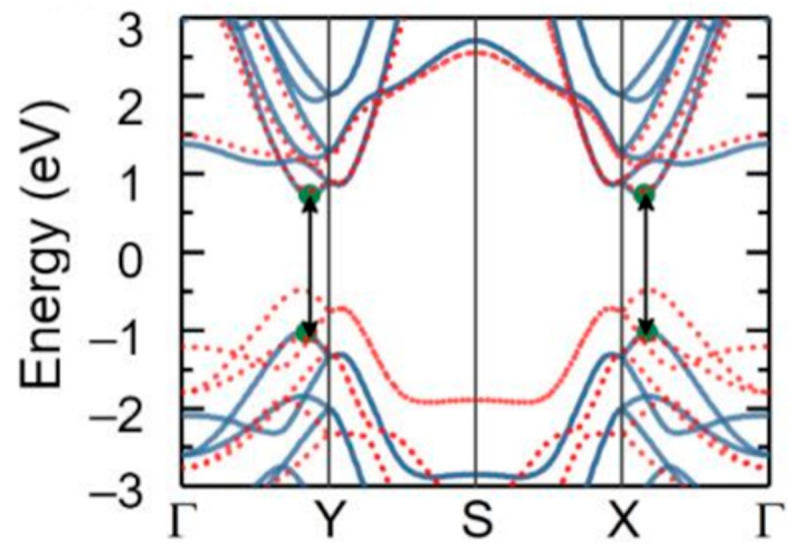
Band structure of monolayer PbTe determined by DFT calculations. Band structure of monolayer PbTe determined by DFT calculations (red dotted lines: the PBE functional; blue lines: the GW method; Green circles: global CBM or VBM). Reproduced from [211] under permissions from copyright clearance center.

Using first-principles calculations, Bafekry et al. [202] examined the optical characteristics of monolayer PbTe and reported the material’s bandgap, refractive index, dielectric function, and α(ω). When taking SOC into account, they found that the bandgap for PbTe increases with decreasing layer thickness down to a monolayer, with values of 0.4 eV at the bilayer, 0.25 eV at the trilayer, and 0.2 eV at the tetralayer. To investigate the PbTe monolayer, Wang et al. [211] used DFT calculations and adopted the GGA of the PBE functional for the exchange-correlation energy as implemented in the VASP. They reported a bandgap energy of 1.742 eV. Their suggested band structure is displayed in Figure 72.

**Figure 73:**
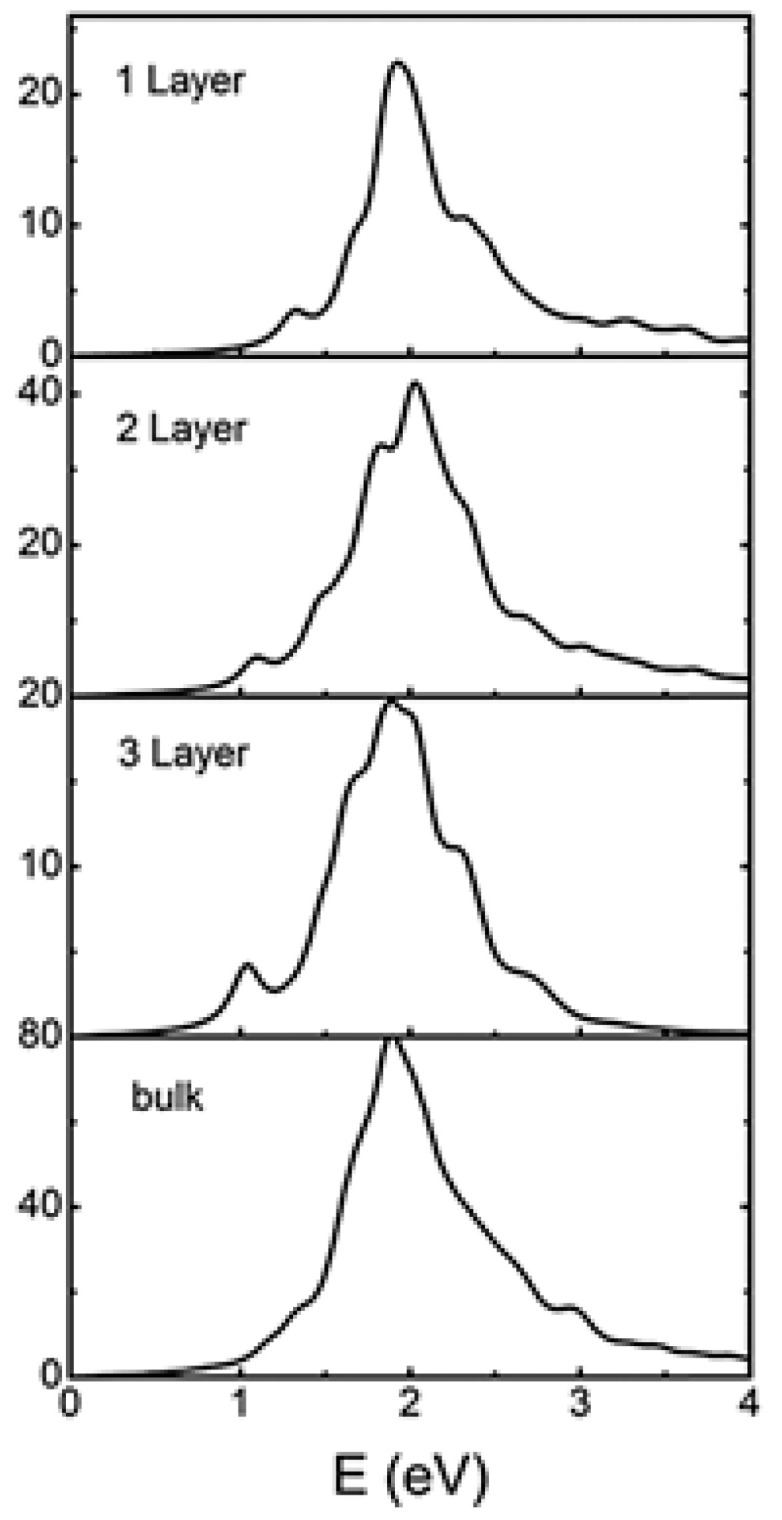
Variations of ε_2_(ω) with energy. Reproduced from [212] under permissions from copyright clearance center.

Li et al.’s [212] report on light adsorption in mono, bi, tri, and bulk PbTe reveals that this energy range is higher, as first principle calculations indicate. Figure 73 displays variations of ε_2_(ω) for PbTe systems. The maximum light absorption occurs at 2 eV, suggesting possible consequences for solar cells. They said that the SOC effect significantly impacts ε_2_(ω) of bulk PbSe and PbTe. 

Moreover, the electrode employed significantly impacts a device’s optical and current-voltage (I–V) properties [213]. Electrodes in optoelectronic devices interact with light in addition to making electrical connections. Transparency, work function, and interface characteristics are just a few of the physical attributes that can be impacted by the selection of electrode materials. In systems like solar cells, LEDs, and photodetectors, the transparency of the electrode is crucial in terms of optical properties [214]. The light must reach the active layer through transparent conductive electrodes, such as silver nanowires or indium tin oxide (ITO), to maximize light emission or absorption [215]. Conversely, light may be reflected or absorbed by metallic electrodes, which are usually opaque, decreasing the device’s optical efficiency. In some systems, the reflectivity of metallic electrodes can also be used to increase the optical field by functioning as mirrors to reflect additional light into the active area. The electrode type determines the electrical contact between the electrode and the active material for I–V characteristics. The work function of the electrode material and the energy level alignment with the semiconductor or active layer are two examples of the parameters that control this [216,217]. High contact resistance can be caused by an incompatibility in work functions, which restricts the effective injection or extraction of charge carriers. Additionally, the device’s overall carrier mobility and recombination rates are impacted by the individual electrodes’ varied interface characteristics with the active layer. Better current flow results from high-quality, low-resistance contacts, but poor-quality interfaces might create obstacles to charge transfer, which lowers I–V performance. However, the optical properties of SnSe, SnS, SnTe, PbS, PbSe, and PbTe have all been discussed in this section. Each of these materials has distinctive qualities that are essential for optoelectronic applications. Because of their high absorption coefficients in the visible region and layered architectures, SnSe and SnS exhibit substantial anisotropy in optical absorption. They hold great promise for use in photovoltaic and photodetector applications. SnTe is a good choice for infrared detectors and sensors because, like lead chalcogenides, it exhibits good infrared absorption properties. Because of their well-known small bandgaps and potent infrared absorption, lead-based chalcogenides like PbS, PbSe, and PbTe are excellent for thermophotovoltaic and infrared detector applications. PbTe’s adjustable bandgap makes it suitable for both optical and thermoelectric devices, while PbSe, in particular, has improved optical properties in the mid-infrared range. These materials’ distinct optical properties result from their electronic band structures and crystal structures, which enable customization for various optoelectronic uses, such as infrared detectors and solar cells.

Table 4 shows the optical properties of Sn/Pb X (X = S, Se, Te). 

## 6. Applications

The layered MX (M = Sn, Pb, and X = S, Se, Te) materials have been used primarily for various photodetection and photo-sensing applications in UV, visible, and near-infrared regions because of their moderate band gaps. Among these, Pb-based compounds have found less practical applications because of the toxic nature of Pb, although much theoretical and practical research has been done so far [173].

### 6.1. SnSe Applications

**Figure 74:**
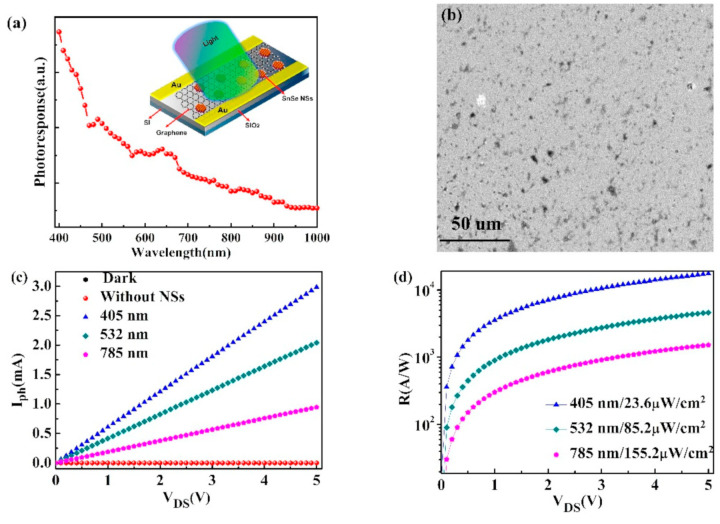
(**a**) The graphene–SnSe NSs photodetector’s spectral response. Inset: A three-dimensional image of the graphene–SnSe NSs photodetector. (**b**) SEM picture of the graphene film’s SnSe NSs. (**c**) Photocurrent of the hybrid device as a function of VDS at various light wavelengths (VG = 0 V, light power density of 155.2 μW/cm^2^). (**d**) Responsivities under various light wavelengths, each with the lowest power intensity, as a function of VDS (VG = 0 V). Reproduced from [51] under permissions from copyright clearance center.

Two-dimensional NSs of single-layered SnSe allotropes can change the incident light’s polarization orientation to determine the sensitivity’s direction due to their anisotropic spin transport and strong visible-light absorbance, possibly using integrated polarization detection SW-NIR optical applications. For use in photodetector applications, Li et al. produced SnSe NSs on SiO_2_/Si substrates using the LPE approach. According to their report, in the visible regime, the SnSE NSs had short carrier recovery times (τ_1_ = 0.77 ps, τ_2_ = 8.3 ps, and τ_3_ = 316.5 ps) and broadband absorption. These details point to their possible uses in the disciplines of fast optics and optoelectronics for creating broadband and high-speed photodetectors. The estimated photoresponse times were 22.6 ms, 11.6 ms, and 9.7 ms, according to the reports [51]. Figure 74a shows the optical response of the phototransistor for V_DS_ = 5 V. The NS–monolayer graphene hybrid photodetector schematic is shown in Figure 74a inset. The SEM picture of the SnSe NSs on top of the graphene channel is displayed in Figure 74b. Junctions between the SnSe NSs and graphene arise due to the discontinuous distribution of SnSe NSs on graphene. Figure 74c displays the changes in drain current with VDS at various excitation wavelengths. The monolayer graphene device did not exhibit any changes in photocurrent when exposed to light. The maximum photocurrent of around 3.00 mA was achieved when 405 nm light was illuminated (VDS = 5 V). Because the carrier builds up in the graphene layer from SnSE NSs, the current varies along with variations in optical excitation [20]. The photodetector’s responsiveness to various excitations is displayed in Figure 74d. Figure 74d illustrates how the responsivity diminishes as the wavelength of light increases. So, SnSe nanosheets (NSs) possess substantial optical absorption in the visible region and low carrier recovery periods, which make them highly promising for application in high-speed, broadband photodetectors. These characteristics make them perfect for use in optoelectronics and rapid optics applications, like the creation of responsive photodetectors at different wavelengths.

**Figure 75:**
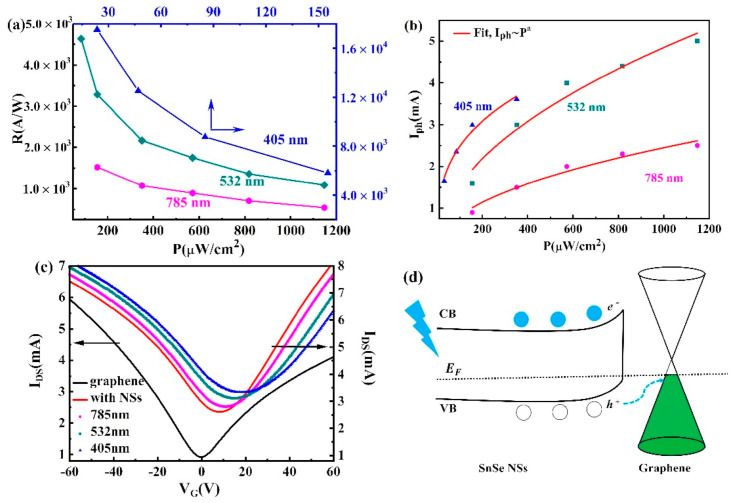
(**a**) Photocurrent and (**b**) responsiveness at varied illumination powers and wavelengths (**c**) The graphene–SnSe NSs photodetector’s transfer characteristics at 405, 532, and 785 nm, respectively. (VDS = 0.5 V; light power density of 155.2 µW/cm^2^). (**d**) A diagram showing how the photo-response mechanism works. Reproduced from [51] under permissions from copyright clearance center.

Figure 75a showed a nonlinear decrease in responsivity when incident power was increased for all excitations by the detector. Higher incident power causes an increase in carrier concentration because of photoexcitation. Li et al. also reported a decrease in α(ω), which caused the responsivity to reduce as the wavelength of light increased. They also used the equation I_ph_ = CP^a^, where an is the ideal ratio of photocurrent to light power density, and C is the proportional constant and index to report the dependency between the incident light power density and the photocurrent. However, a = 1.0 is the optimum photodetector. They calculated the value of a by fitting the graphs for variations in photocurrent with power density, and they reported that value to be 0.42, 0.5, and 0.47 for wavelengths of 405, 532, and 785 nm, respectively. So, it is concluded that these detectors are perfect for optical sensors and power-adjustable detection systems in fields like imaging, communications, and environmental monitoring because of their nonlinear decrease in responsivity with increased incident power and their specific dependence of photocurrent on light power density. 

**Figure 76:**
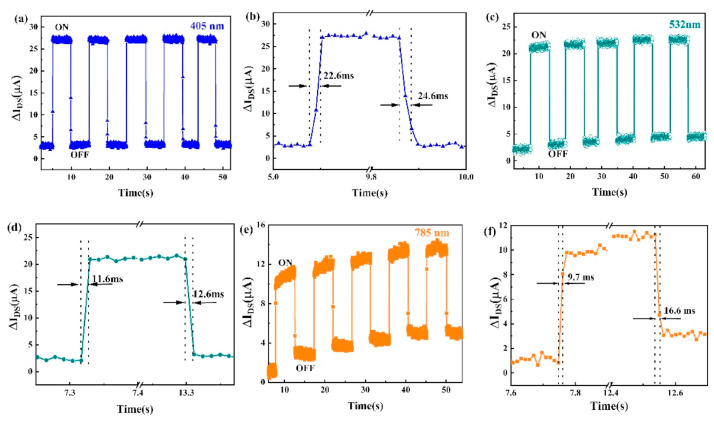
Time-resolved channel photocurrents are used to display the rise and fall periods for (**b**) 405 nm (**d**) 532 nm (**f**) 785 nm, as well as channel photocurrent response to on/off light illumination (**a**) 405 nm (**c**) 532 nm (**e**) 785 nm over multiple cycles. Reproduced from [51] under permissions from copyright clearance center.

Figure 75c displays the transfer curves at V_DS_ = 0.5 V both before and after the SnSe NSs decorating at different illumination wavelengths. They observed that, compared to the single monolayer graphene device, the hybrid device’s Dirac point was positioned at +8 V after the SnSe NSs were decorated, and it exhibited a more excellent conductivity in dark conditions. Furthermore, the Dirac point moved to the higher positive gate voltage during illumination. The Dirac point’s largest shift was at +18 V under 405 nm of light, and its smallest shift was under 785 nm. They attributed these shifts to either low optical absorption or low excited photon energy. They concluded that although photoproduced electrons are confined in the SnSe NSs, the interface contacts between SnSe NSs and monolayer graphene enable the injection of photogenerated holes from SnSe NSs into monolayer graphene upon illumination. These phenomena can be further explained by looking at the schematic of the photo-response scenario shown in Figure 75d. They determined that the channel photocurrent responses were performed at different wavelengths for an incident power density of 155.2 W/cm^2^, V_DS_ = 0.05 V, and V_G_ = 0V. Figure 76a–f shows these. They observed that the channel photocurrent decreases with an increase in the wavelength of excitation lights, although the detector’s response (in terms of rise and fall time) improves, providing faster responses for higher excitation wavelengths. They came to the conclusion that the channel current was divided into two processes: slow and quick. The rapid process of carriers moving from SnSe NSs to graphene after the light is turned on is responsible for the rapid rise at higher wavelengths. In addition, because electrons and holes are separated spatially, which causes a gradual drop in channel current, electron-hole recombination may be delayed while the light is off.

**Figure 77:**
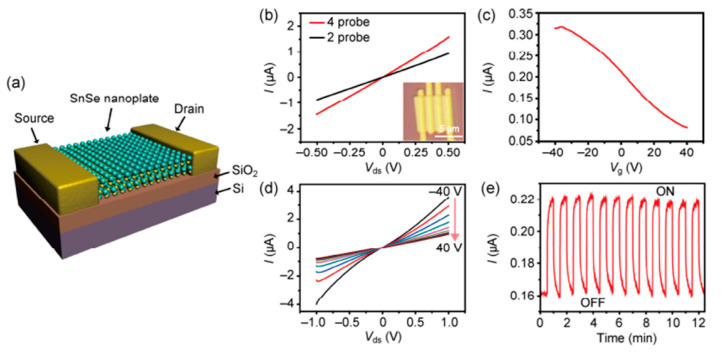
(**a**) SnSe nano plate transistor schematic view in three dimensions. (**b**) The device’s two four-probe $I-V_{ds}$ characteristics. The device’s optical microscope image is inset; the scale bar is 5 μm. (**c**) The device’s transport characteristic curve at V_ds_ = 0.1 V. (**d**) The output characteristic curves at various V_g_ values (ranging from –40 V to 40 V in steps of 10 V). (**e**) The photocurrent response time trace for the device at a bias voltage of 0.1 V when the incident light is turned on and off. Reproduced from [219] under permissions from copyright clearance center.

The magnetron sputtering method was employed by Hao et al. to create SnSe NSs on SiO_2_/Si substrates. Based on this technique, a photodetector was found to be very sensitive throughout a wide wavelength range, spanning from UV to visible to NIR. Their findings [52] indicated a maximum responsivity of 277.3 A/W. Similarly, Zhong et al. used a hot injection technique to produce SnSe NSs, which were then placed on a Si/SiO_2_ substrate in order to manufacture photodetectors. Under 400 nm illumination (0.46 mW/cm^2^), they reported that the photocurrent (I_ph_), responsivity (R), and external quantum efficiency (EQE) were 13.7 mA/cm^2^, 30 mA/W, and 7.69%, respectively. The photocurrent was I_ph_ = 4.3 mA/cm^2^, and the responsivity was 11 mA/W under 1050 nm light (0.4 mW/cm^2^). The Gr/SnSeNS/Gr photodetector demonstrated detection throughout a broad wavelength range, ranging from visible to NIR. They recorded a maximal responsivity of 38 mA/W [218]. Additionally, Zhao et al. synthesized SnSe NSs to create photodetectors and used electron beam lithography to deposit them on SiO_2_ substrate (Figure 77a). The photoresponsivity was determined to be around 330 A/W under white light illumination and a bias voltage of 0.1 V [219]. Figure 77b–d depicts the additional features of the SnSe nanoplate photodetector. The creation of extremely sensitive, broad-spectrum photodetectors that can function well over an extensive wavelength range, from ultraviolet (UV) to near-infrared (NIR), is the primary finding of this research. These photodetectors have a high level of sensitivity, making them appropriate for use in optical sensing, imaging systems, and optoelectronic devices where it’s essential to detect at different wavelengths, including in communications, medical imaging, and environmental monitoring.

Yu et al. created a polarization-sensitive photodetector with Au electrodes by synthesizing SnSe NSs and depositing them on a SiO_2_/p++ Si substrate. They observed that the EQE, detective, and responsivity at 808 nm light illumination were 1422%, 4.08 × 10^10^ Jones, and 9.27 A/W, respectively. The detector displayed a broad spectrum response between 360 and 1550 nm [166]. SnSe thin films were deposited on PET templates by Xu et al. to create SnSe thin film photodetectors. According to their research, the photodetector demonstrated a high responsivity of 1745.5 A/W and detectivity of 4.2 × 10^11^ Jones when illuminated by a 404 nm laser. The photodetector demonstrated 3.9 × 10^7^ Jones detectivity and 0.16 A/W responsivity when illuminated by a 10.6 μm laser [220]. 

### 6.2. SnS Applications

**Figure 78:**
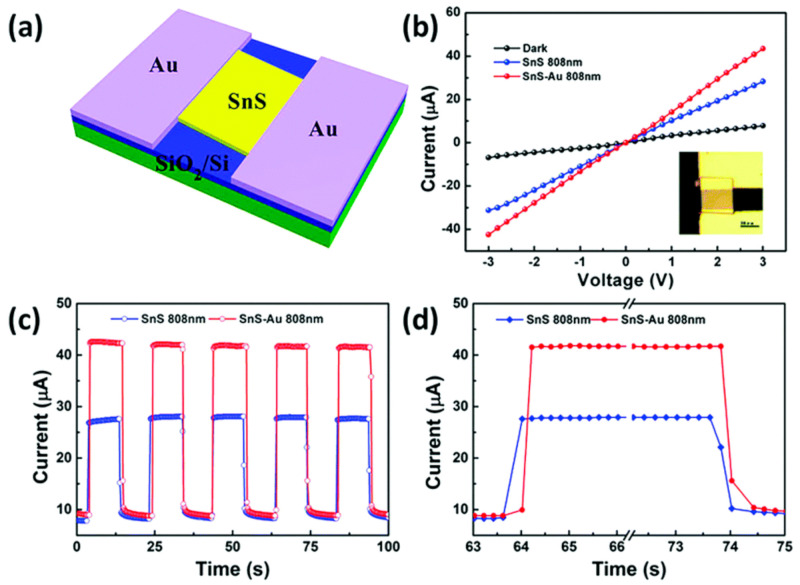
(**a**) Three-dimensional view of the photodetector. (**b**) I–V curves with optical microscope picture inset, (**c**) time-resolved photoresponse, and (**d**) response and recover time of the Au-NP-decorated device and the SnS nanosheet-based device under 808 nm light irradiation and in the dark, respectively. Reproduced from [60] under permissions from copyright clearance center.

SnS crystals have a direct band gap ~ of 1.3 eV, a high α(ω), and higher photoconversion efficiencies, which makes them a good candidate for photodetectors. Because self-assembled SnS nanoflakes have a larger surface-to-volume ratio than other materials, they can produce photodetectors with superior photo-response characteristics, quick response and recovery times, long-term stability, and a strong dependence of photocurrent on light intensity [221]. Zhang et al. Fabricated a NIR SnS nanosheet-based photodetector on a mica substrate. They used two device configurations while they fabricated the photodetector. The first device type includes monolayer SnS NSs, while for the second device type, they decorated the NSs with Au nanoparticles. They reported a photoresponsivity of 365 A/W for the first type when an 808 nm light of 40 mW/cm^2^ intensity was applied [60]. The photodetector’s 3D structure is depicted in Figure 78a. The I–V curve of a photodetector with three configurations is displayed in Figure 78b. The I–V graph with black lines represents the SnS nanosheet device under dark, while the blue lines represent the device under illumination. A two-terminal device’s optical microscope image is displayed in the inset of Figure 78b. When exposed to laser light, the photocurrent rises dramatically due to increased excited electron-hole pairs, which raises the channel’s conductance. As seen in Figure 78c (blue line), the device demonstrated a stable reaction when the incident light was turned on and off alternately with a time interval of 10 s under a bias of 3 V. Figure 78d shows the response and recovery, which are both measured to be 0.35 s. The photoconductor’s EQE and detectivity (D*) were 5.70 × 10^4^% and 2.72 × 10^9^ Jones, respectively. They used 40 nm-diameter Au nanoparticles to embellish the SnS NSs to increase the photo response of the 2D SnS nanosheet-based photodetectors. Figure 78b (red line) also displays the matching I–V curve. The photocurrent increased from 28.25 mA to 43.39 mA at 3 V, which they judged to indicate an improvement in photo response performance. By effectively capturing incident light, the Au nanoparticles generated a localized surface plasmon resonance effect, which improved photocurrents in photodetectors. In that order, the Au-decorated device’s responsivity, EQE, and D* are computed to be 635 A/W, 9.92 × 10^4^%, and 4.74 × 10^9^ Jones.

**Figure 79:**
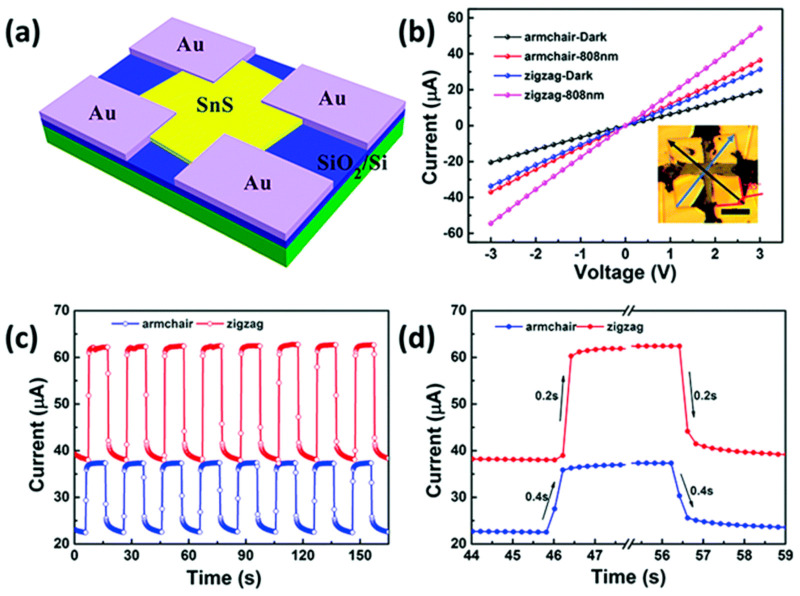
(**a**) Three-dimensional view of the photodetector with four ports. (**b**) I–V curves with optical microscope picture in the set, (**c**) time-resolved photoresponse, and (**d**) response and recovery time of the SnS nanosheet-based apparatus under the illumination of 808 nm light in the zigzag and armchair directions, respectively. Reproduced from [60] under permissions from copyright clearance center.

Figure 79a shows the fabrication of four-terminal devices to investigate the anisotropic photoresponse of 2D SnS nanosheet (NS) photodetectors. The four terminal devices’ I–V curves are displayed in Figure 79b. They reported a dark current of 31.3 mA along the zigzag route at a bias voltage of 3 V, which was much more than 19.3 mA along the armchair direction. The photocurrent measured along the armchair direction was found to be 36.4 mA. In contrast, the photocurrent measured along the zigzag direction grew to 54.2 mA when illuminated with light at 40 mW/cm^2^. The fluctuations in currents with time demonstrate good repeatability and stability, as shown in Figure 79c. According to Figure 79d, the device’s response and recovery times are 0.2 s in the zigzag direction and 0.4 s in the armchair direction, respectively. Because the effective mass in the zigzag direction is lower than that in the armchair direction, resulting in a greater carrier wavenumber, anisotropy in current flow and response time is produced.

Liu et al. synthesized SnS nanoflakes by CVD on Si/SiO_2_ substrate, reporting a maximum photoresponsivity of 156.0 A/W, EQE of 4.77 × 10^4^%, and normalized detectivity of 2.94 × 10^10^ Jones [71]. Similarly, SnS nanoflakes were created by Dong et al. on substrates made of polyethylene terephthalate. Based on them, a photodetector exhibited a broad spectrum response from 355 nm to 1550 nm. Under an ultraviolet laser with a wavelength of 355 nm, they reported maximum photoresponsivity and detectivity of 1280 A/W and 3.02 × 10^11^ Jones, respectively, and obtained a responsivity of 69 A/W towards the 1550 nm region [223]. Wen et al. synthesized SnS NSs that were created using PVD and a photodetector based on them. The photodetector showed a high responsivity of 161 A/W EQE of 4.45 × 10^4^% and detectivity of 1.15 × 10^9^ Jones under 450 nm blue light illumination [224]. Krishnamurthi et al. produced multilayer SnS from metallic liquid tin with thicknesses ranging from a single unit cell to several stacked unit cells achieving broad spectral responses from deep ultraviolet to NIR regions (280–850 nm). Single unit cell thick SnS shows responsivity, detectivity, EQE, response time of 927A/W, 1.09 × 10^9^ Jones, 1.7 × 10^5^% and 0.12 ms respectively at 660 nm wavelength and for multiple unit cell layer SnS shows responsivity, detectivity, electrical quantum efficiency, response time of 3.51 × 10^3^A/W, 6.83 × 10^10^ Jones, 1.5 × 10^5^% and 0.16 ms, respectively, as reported by them. These results show that SnS is a promising candidate for photodetection in the NIR and infrared zone [75]. Additionally, Luo et al. synthesized SnS and SnS_2_ nanoflakes by phase-controlled synthesis. Phototransistors based on them showed promising results. They reported a high responsivity of 3390 mA/W, a detectivity of 1.1 × 10^10^ Jones, and a fast response and recovery time of 3.10/1.59 ms [225]. Modi et al. synthesized SnS NSs using a solvothermal process. At low intensity, the photodetector showed a photoresponse of 0.02 mW/cm^2^. They stated that the photodetector had a response time of 150 ms, a sensitivity of 10^10^ Jones, and a responsivity of 86.2 mA/W. Over a large area, the photodetector displayed strong absorption [65]. So, for the creation of broad-spectrum photodetectors with outstanding performance in the visible, near-infrared, and ultraviolet (UV) domains, SnS nanoflakes and nanosheets are extremely promising materials. With their high detectivity, quick response times, robust light absorption, and high responsivity, these photodetectors are ideal for use in optoelectronic devices, sophisticated optical sensing, and imaging in industries like communications, environmental monitoring, and medical diagnostics.

**Figure 80:**
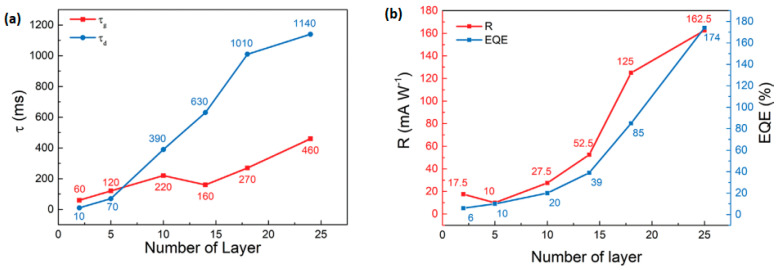
(**a**) Time constants for photoresponse, (**b**) responsivity, and EQE of 2–25 ML SnS photodetectors. Reproduced from [228] under permissions from copyright clearance center.

**Figure 81:**
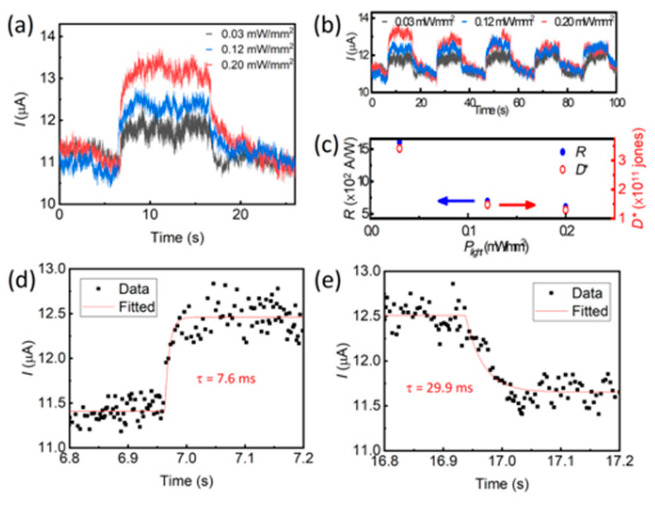
(**a**) The current that varies over time when exposed to varying power densities; (**b**) the photodetector’s consistent photoresponse behavior; (**c**) the detectivity D and responsivity R as functions of power density; and (**d**) the rising and (**e**) falling edges of a single pulse. Reproduced from [67] under permissions from copyright clearance center.

Wang et al. [226] deposited 2D SnS films on Si/SiO_2_ substrates and reported their layer-dependent optical, photo-detecting properties. The devices made of these films showed an on/off ratio of f 3.41 × 10^6^ and mobility of 1.48 cm^2^V^−1^s^−1^. The 2 ML SnS-based photodetector demonstrated a quick reaction under 365 nm light with a power density of 80 µWcm^−2^. Thinner films have fewer flaws, interlayer traps, and in-layer carrier transport. Additionally, the photodetectors showed a broadband response with very high detectivity under dim light irradiation. As seen in Figure 80a, the photodetector in the 2 ML SnS device indicates a quick photocarrier production and quenching speed with a time constant of 60 ms. The fluctuations in EQE and responsivity with layer numbers under 365 nm illumination at 80 µWcm^−2^ are depicted in Figure 80b. Li et al. [227] produced photodetectors based on the graphene/SnS/Si heterojunction. Thermal evaporation was used to create SnS films. The photodetector displays a broadband response from the visible to NIR range. Photodetectors based on vertically grown SnS layers on Si substrates were created by Kumar et al. [228]. The photodetector saw a broadband response in the UV, visible, and NIR range. The photodetector demonstrated fast response times: ~12 µs for rise and ~55 µs for decay. At 760 nm wavelength and 7 mWcm^−2^ intensity, it demonstrated a high detectivity of (~3.2 × 10^14^ Jones) and a responsivity value of 12 mA/W. SnS NSs were created by Huang et al. [229] using the LPE technique. Based on these, the photodetectors displayed a maximum detectivity of 9.43 × 10^7^ Jones and a maximum responsivity of 59.8 μA/W. Broadband band absorption in the UV–visible range was observed. It showed a response time of 0.1–0.3 s. Dong et al. [230] synthesized SnS films using the PVD method and fabricated photodetectors based on an SnS/SnSe_2_ heterostructure. The devices showed photo responses in the UV–visible range and a 14.78 µA/W photoresponsivity. Vinoth et al. [231] manufactured NIR photodetectors based on orthorhombic SnS. The photodetector showed broadband response from the UV–visible–NIR region. The maximum photoresponsivity was reported to be 0.19A/W at 1030 nm wavelength light illumination. Yuan et al. [67] synthesized SnS NSs by PVD. The NSs were 12 nm thick and had a lateral size of 7 µm. The time-dependent current is displayed in Figure 81a. After testing the photodetector’s photoresponse behavior for the aforementioned power densities, they discovered that it is repeatable, as seen in Figure 81b. As Figure 81c demonstrated, the photodetector based on the NSs exhibited strong responsivity of 1604 A/W, detectivity of 3.42 × 10^11^ Jones, and EQE of 2.31 × 10^5^% under 850 nm wavelength light irradiation. Furthermore, as Figure 81d illustrates, they calculated the response time of 7.6 ms and recovery time of 29.9 ms from a single pulse.

Mahdi et al. [232] synthesized SnS nanoflakes by the CBD method on glass substrates for photodetector applications. It showed broadband photoresponse in the UV–visible–NIR region. In the 300–930 nm wavelength range and at a bias voltage of 5 V, the photodetector shows an average responsivity of 0.01mA/W. And under 530 nm light illumination, it shows a rising time of 0.36 s and a decaying time of 0.38 s. He et al. [233] synthesized a few layers of SnS and fabricated photodetectors based on Au/SnS/Ag Schottky structure. This photodetector shows broadband photoresponse from visible–NIR. The photodetector exhibits a strong responsivity of 1652.87 A/W, detectivity of 8.05 × 10^12^ Jones, and response time of 6.5 ms when illuminated by light at 850 nm. Balakarthikeyan et al. [234] fabricated photodetectors based on SnS thin films prepared by the thermal evaporation method. They reported the films’ responsivity, detectivity, EQE, response time, and recovery time to be 6.4 × 10^−2^ A/W, 6.05 × 10^9^ Jones, 14.9%, 1.5 s, and 2.5 s, respectively. Wang et al. [235] synthesized SnS thin crystals by solvothermal method. The thin crystals had a lateral dimension of 20–30 µm and a thickness of less than 10 nm. For 532 nm light irradiation with a power intensity of 50 mWcm^−2^, the photodetector based on it demonstrated strong responsivity of 2040 A/W, detectivity of ~3 × 10^9^ Jones, EQE of ~4.75 × 10^5^%, and response time of ~90 ms. Patel et al. [236] fabricated photodetectors based on SnS layers on n-Si substrates. The SnS layers were covered with ITO. They reported the photodetector’s responsivity at –1 V bias voltage to be 0.25 A/W and detectivity to 1.3 × 10^11^ Jones with fast response and recovery time of 41 µs and 40.7 µs, respectively, under 850 nm light illumination. At low intensity of 10 µW/cm^2^, the photodetector showed responsivity and detectivity of 1.19 A/W and 7.1 × 10^11^ Jones, respectively, with photoresponse time of 222 µs and 80 µs for rise and decay. 

### 6.3. SnTe Applications

The strain-sensitive nature of the 2D SnTe monolayer makes it a promising material for mechanical sensor design. Liu et al. [83] synthesized 3.6 nm thick SnTe nanoplate by the van der Waals growth process and then deposited them on mica substrates to produce photodetectors. Based on these, photodetectors had an EQE of 88.5% when exposed to a 980 nm laser at ambient temperature without needing a gate voltage. The responsivity rises from 0.698 to 1.468 A/W upon increasing thickness to 35 nm. The device showed no apparent deterioration following 300 bends at different radii. Song et al. [84] synthesized SnTe nanofilms by a magnetron sputtering technique on a quartz substrate. Photodetectors were prepared based on them, and Cr/Au was used as an electrode. According to their study, the photodetectors demonstrated a broad spectrum of photoelectric response. In NIR illumination at 940 nm power density and 1.71 AW^−1^ responsivity (R) and 3.46 × 10^11^ cmHz^1/2^W^−1^ detectivity (D*) were recorded for the device.

**Figure 82:**
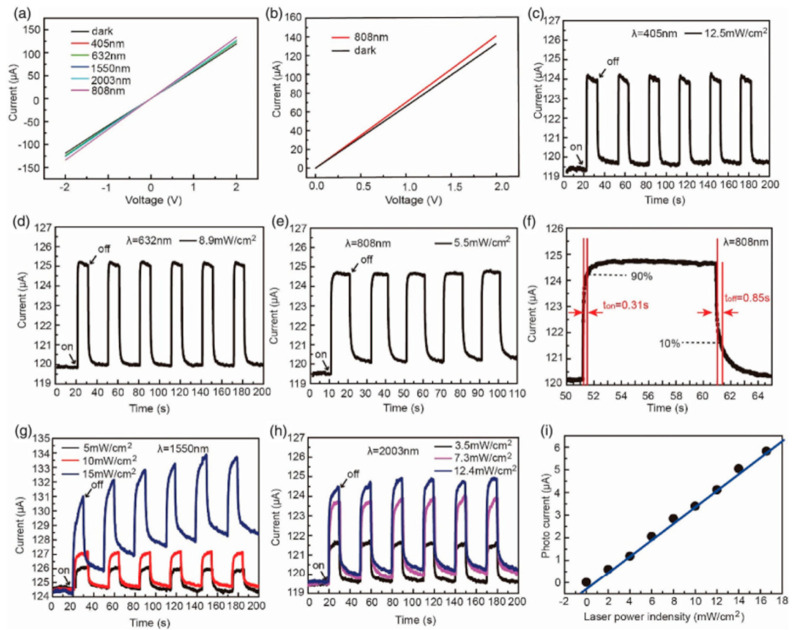
(**a**) I–V curves during laser irradiation at 405, 632, 808, 1550, and 2003 nm and in the dark. (**b**) I–V curve stimulated by an 808 nm laser. (**c**–**e**) Time-dependent photoresponse at 2 V bias voltage and 405, 632, and 808 nm laser wavelengths, respectively. (**f**) Photodetector response and reset cycle when exposed to an 808 nm laser. (**g**,**h**) Time-dependent photoresponse at different laser power intensities at 1550 and 2003 nm laser wavelengths, respectively, with a voltage bias of 2 V. (**i**) Variations of photocurrent with a laser power intensity of the device. Reproduced from [237] under permissions from copyright clearance center.

Jiang et al. [237] synthesized SnTe NSs by MBE on SrTiO_3_ substrate. It showed a flat, stable response over a broadband region from visible to mid-infrared. According to their report, this photodetector exhibits a 3.75 AW^−1^ responsivity at 2003 nm at room temperature and a quick and consistent photo response throughout the visible to the mid-infrared spectrum. The photodetector was made based on the SnTe/Bi_2_Te_3_ strip with SrTiO_3_ as the supporting substrate, and Cr/Au electrodes were used. Then, they measured under 405 nm, 632 nm, 808 nm, 1550 nm, and 2003 nm. Figure 82a shows the *I–V* curves of the device under the dark and illumination of lasers of different wavelengths. Figure 82b shows the photocurrent output signal under the illumination of an 808 nm laser and the time-dependent response of the photodetector for the wavelengths 405 nm, Figure 82c–e 632 nm, and 808 nm. The laser power intensity was 2.8, 8.9, and 5.5 mWcm^−2^, respectively. Figure 82f shows the complete cycle under an 808 nm laser; the rise and falling time of the photodetector were calculated to be 0.31 s and 0.85 s. Figure 82g,h shows the photocurrent response at different incident laser power intensities of 1550 nm and 2003 nm. At 15 W/cm^2^, the dark current increased step by step, and the current signal did not return to its original state even after turning off for 20 s. Figure 82i shows the linear dependence between laser power intensity and photocurrent, and from it, they calculated the photoresponse of the detector under 808 nm to be 3.75 A/W. 

**Figure 83:**
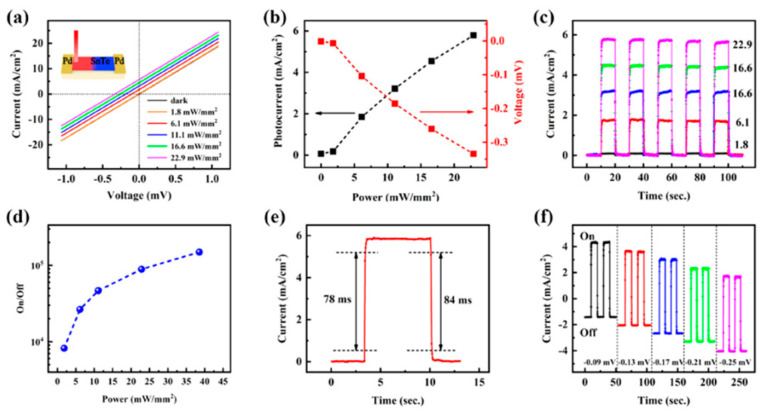
(**a**) The device’s J–V curves under various power intensities of a 532 nm laser. (**b**) The photovoltages and photocurrents flow through the apparatus’s power dependence. (**c**) The device’s time-dependent photoresponse at varying power intensities. (**d**) On/Off ratio dependence with power intensity. (**e**) Separated response cycle, 78 ms rising and 84 ms falling time. (**f**) Photoresponse under different bias voltages. Reproduced from [238] under permissions from copyright clearance center.

Liu et al. [238] synthesized SnTe thin films using the magnetron sputtering method, deposited them on polyethylene terephthalate templates and used Pd as electrodes to produce photodetectors. These photodetectors demonstrated stable photoresponse throughout a wide spectral range. It displayed a maximum detectivity of 1.3 × 10^10^ Jones, a responsivity of 3.9 mA/W, and a quick response and recovery time of 78/84 ms when illuminated by a 404 nm laser. Additionally, it showed outstanding adaptability for wearable optoelectronics with minimal energy consumption. Figure 83a shows the J–V curve of SnTe PTE-based photodetectors. The power dependency of photocurrent is depicted in Figure 83b; it is clear that when power intensity increases, the photothermal transformation is enhanced. Because of this, there may be a rise in the temperature differential between the device’s two ends, which could heighten the TE Seebeck effect’s inherent electrical field. The device’s time-dependent photoresponse at power intensities of 1.8, 6.1, 11.1, 16.6, and 22.9 mW/mm^2^ is displayed in Figure 83c. The On/Off ratio is displayed in Figure 83d, and the rising and falling times are shown in Figure 83e. It is evident from Figure 83f that when the laser is turned on or off, the current alternates between positive and negative. This demonstrates the device’s potential for usage as a dependable binary response device for on/off photodetection of a faint light.

**Figure 84:**
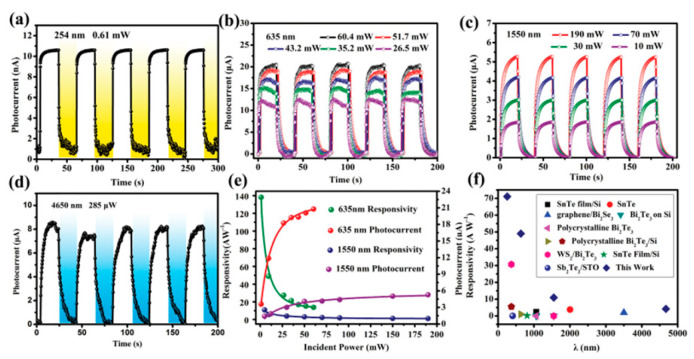
(**a**–**d**) Time-dependent photocurrent response at 254, 635, 1550, and 4650 nm, respectively. (**e**) Photocurrent and photoresponsivity dependence with incident power intensity. (**f**) Ragone plots of wavelength versus responsivity for the SnTe device compared to another photodetector with other photodetectors. Reproduced from [239] under permissions from copyright clearance center.

**Figure 85:**
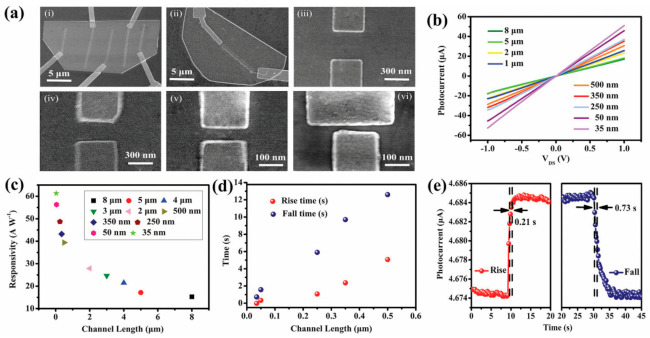
SEM pictures (**a**) of SnTe FETs with different channel lengths: (**i**) 5, 4, 3, and 2 µm; (**ii**) 500 nm; (**iii**) 350 nm; (**iv**) 250 nm; (**v**) 50 nm; (**vi**) 35 nm. (**b**) Photocurrent with varying channel lengths about source–drain bias. (**c**) Ragone plots showing the SnTe-based photodetector’s channel length against responsivity. (**d**) Ragone plots of the SnTe-based photodetector’s channel length vs rising time. (**e**) Response of the photocurrent in time. The time of ascent (≈0.21 s) and descent (≈0.73 s). Reproduced from [239] under permissions from copyright clearance center.

Yang et al. [239] produced photodetectors by depositing 100 nm to 120 nm thick SnTe nanoflakes on Si/SiO_2_ substrates with Cr/Au as electrodes. Their research shows a wide spectral range spanning from deep UV to MIR (254 nm to 4650 nm). The device’s time-dependent photoresponse at 254, 635, 1550, and 4650 nm wavelengths is displayed in Figure 84a–d. The photocurrent exhibits an excellent and consistent amplification at varying light intensities, and each illumination cycle’s line form is similar, indicating high photoswitching repeatability. The photocurrent grows linearly at lower power intensities, as seen in Figure 84e, which also displays the photoresponsivity and photocurrent dependence with incident power intensity. Scaling of the channel length was introduced to enhance the performance of photodetectors. They created SnTe FETs, the SEM pictures displayed in Figure 85a, with different channel lengths. They employed a 53.4 mW, 635 nm laser to evaluate the photoconductors. The I–V curve of the photodetectors with varying lengths of channel is displayed in Figure 85b. The Ragone plots in Figure 85c–e display the photoresponsivity with varying channel lengths and rise and fall times about channel lengths. They stated that the exhibits a high 61.3 A/W responsivity.

### 6.4. PbS Applications

**Figure 86:**
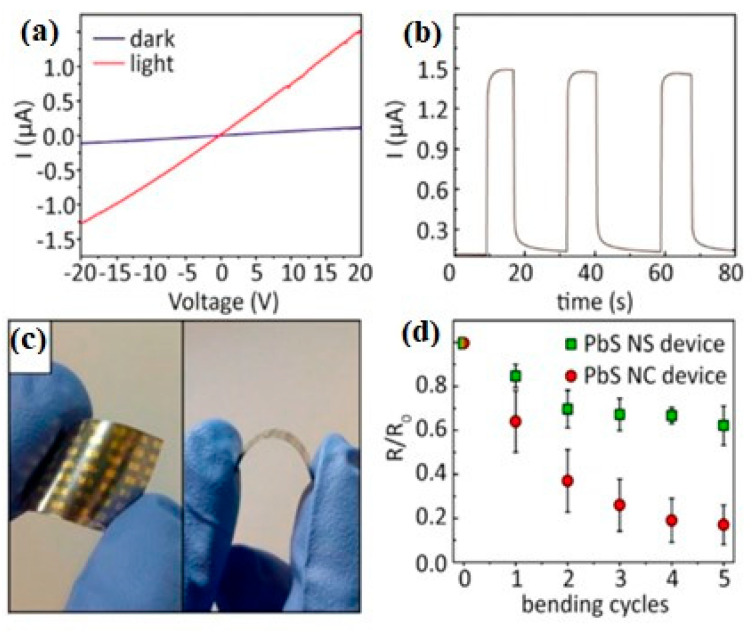
(**a**) PbS NS-based device’s I–V characteristics with a 100 μm Ti/Au contacts gap; (**b**) On/Off response to white LED light at 20 V bias voltage. (**c**) Images captured during a bending cycle (**d**) The normalized responsiveness (R/R0) of flexible PbS NC or NS devices after bending cycles. Reproduced from [109] under permissions from copyright clearance center.

A flexible PET substrate-based PbS photodetector shows great promise for application in flexible optoelectronic devices. This device is suitable for flexible, high-performance photodetectors in applications like wearable electronics, flexible displays, and portable optical sensing systems because of its Ohmic I–V characteristics, ON/OFF ratio of 10.5, and improved performance due to reduced contact spacing. Figure 86a displays the Ohmic I–V characteristics of the PbS photodetector that Akkerman et al. [109] found after determining the characteristics under dark and light circumstances while fabricating it on a PET substrate. They used a bias voltage of 20 V, white light with a wavelength range of 400–750 nm, and an intensity of 100 mWcm^−2^. They discovered an ON/OFF ratio of around 10.5 ± 0.6, as shown in Figure 86b. A similar device on a flexible substrate is shown in Figure 86c. Reducing the spacing between the contacts from 100 μm to 10 μm enhanced the zT, as demonstrated in Figure 86d. 

**Figure 87:**
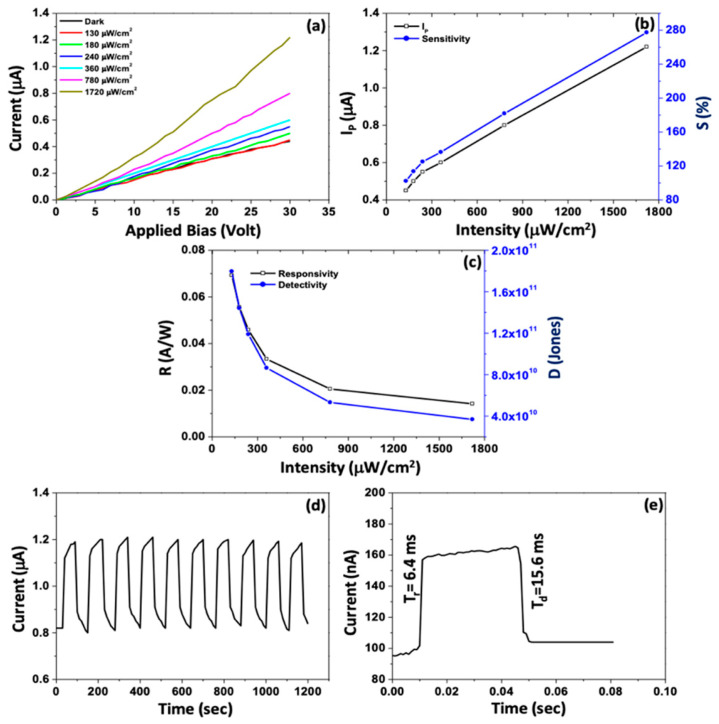
(**a**) Ag/PbS/Ag photodetector device I–V characteristics in the dark and in IR illumination at intensities between 130 and 1720 µW/cm^2^, (**b**) changes in photocurrent and sensitivity with intensity, (**c**) changes in responsivity and detectivity with intensity, (**d**) photocurrent measured under consecutive light On/Off cycles, and (**e**) real-time photo response. Reproduced from [241] under permissions from copyright clearance center.

Wang et al. [240] synthesized PbS NSs on NaCl and KCl substrates by CVD method and then transferred them to produce photodetectors. They reported these photodetectors to have a fast response time of about 0.25 s. They discovered the device’s I–V characteristics were linear when exposed to a 40 mW/cm^2^ 450 nm laser. Gu et al. [110] fabricated photodetectors based on 50 nm thick PbS nanoplates synthesized by CVD. They found that when the incident light power drops, the photoresponsivity increases. Wu et al. [99] fabricated photodetectors based on PbS nanoplates with Cu_2_S residues on a Si/SiO_2_ substrate and used Au as an electrode. Under 808 nm laser illumination, they reported the photodetector’s responsivity to be ~1739 A/W and detectivity of 2.55 × 10^11^ Jones, thus showing potential as an NIR photodetector. To measure the I–V characteristics of eight-layer thick PbS nanoplates, they used an 808 nm laser with 0.5 mW illumination. Thabit et al. [241] synthesized a nano 50 nm thick PbS thin layer on a glass substrate. Its maximal responsivity was 70 mA/W, with a detectivity of 1.8 × 10^11^ Jones. The photoresponse measurement on the PbS thin-film device, while the applied bias varies between 0 and 30 V, is depicted in Figure 87a. The graph (Figure 87b) displayed the variations in photocurrent when the device was exposed to different IR intensities, ranging from 130 to 1720 µW/cm^2^. The measurement conducted in the absence of light reveals a linear increase in the dark current up to 30 V with applied bias. The device’s I–V characteristics are linear. The changes in responsivity and detectivity with increasing light intensity are depicted in Figure 87c, where they drop from 70 to 10 mA/W and 1.8 × 10^11^ to 3.7 × 10^10^ Jones, respectively. The cyclic photo response of the Ag/PbS/Ag device was studied at 30 V applied bias when a 60-s pulse with an IR illumination of 1720 W/cm^2^ was delivered (Figure 87d). The response time of PbS devices is comparatively faster (rise and fall periods of 6.4 and 15.6 ms, respectively) in Figure 87e, which is caused by photogenerated charge carriers while in the presence of infrared light.

Khandoze et al. [242] fabricated photodetectors based on PbS films deposited on paper by spray pyrolysis using graphite electrolyte. They stated that the reaction was 0.0356 A/W, the fall time was 6.3 ms, and the quick response was 14.7 ms. Gao et al. [92] synthesized PbS NSs by LPE on ITO substrates to fabricate photodetectors. They reported that these photodetectors showed broad UV–Vis–NIR (300–1200 nm) range absorption. Additionally, they demonstrated remarkable long-term cycling stability, a detectivity of 3.96 × 10^10^ Jones, and a photo-responsivity of 27.81 mA/W.

### 6.5. PbSe Applications

**Figure 88:**
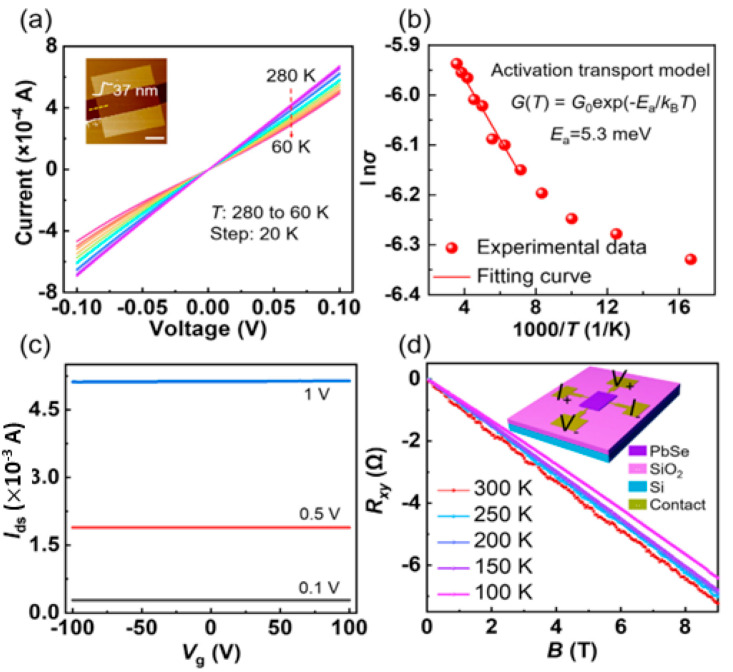
(**a**) I–V properties of 2D PbSe at various temperatures, with steps of 20 K, ranging from 60 to 280 K. The PbSe device’s height profile and AFM picture are seen in the inset. (**b**) Temperature-dependent variations in the 2D PbSe conductivity. (**c**) Drain current-gate voltage (Ids-Vg) transfer curves for a range of drain voltages. (**d**) Hall measurements based on PbSe nanosheet temperature dependency. Reproduced from [88] under permissions from copyright clearance center.

Jiang et al. [88] used PbSe NSs to fabricate the photodetectors. The I–V curves of an in situ-fabricated, two-terminal PbSe device at various temperatures are displayed in Figure 88a. The inset, which has a measurement of 37 nm, provides the matching AFM image and height profile. As the temperature drops, the current progressively drops, suggesting that the 2D PbSe NSs are semiconductors as they expand. From the slope of the conductivity versus temperature graph shown in Figure 88b, they found the activation energy of the 2D PbSe to be 5.3 meV using the Arrhenius equation. 

**Figure 89:**
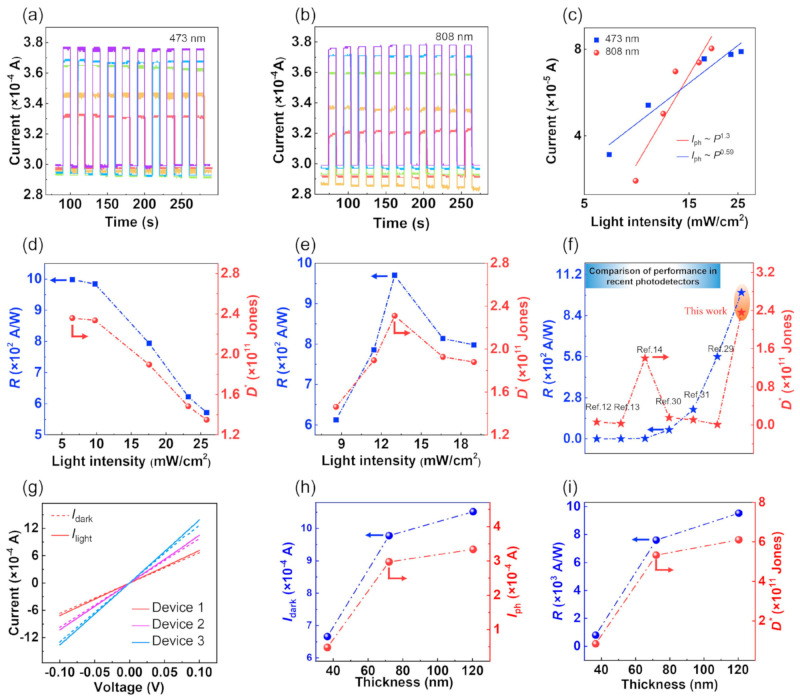
(**a**,**b**) The PbSe device’s temporal response was seen when it was exposed to two different laser intensities: an 808 nm laser with a bias of 0.1 V and a 473 nm laser with a variation in intensity that ranged from 8.58 (**bottom**) to 25.85 (**top**) mW/cm^2^. (**c**) Photocurrents about light intensity. (**d**,**e**) The PbSe photodetector’s light intensity-dependent R and D* under 473 and 808 nm lasers, respectively. The device channel is 16.2 μm long and 32.8 μm wide. (**f**) Comparison of performances in recent photodetectors. (**g**) I_ds_-V_ds_ of the PbSe photodetector at varying channel thicknesses in the lighted (solid) and dark (dashed lines) states, with a 25.85 mW/cm^2^ light power, respectively. (**h**) PbSe photodetector’s thickness-dependent I_dark_ and I_ph_. (**h**,**i**) R and D* of the PbSe photodetector depend on thickness. Reproduced from [88] under permissions from copyright clearance center.

Figure 88c shows the transfer curve of the PbSe detector, where no obvious gate tunability can be seen for these devices. According to Jinag et al. [88], this might be because pervasive vacancies-induced in-gap defect states peg the Fermi level of PbSe NSs generated by CVD. Figure 88d displays the four-terminal Hall bar device and the Hall measurements that were taken. The measurement found that 2D PbSe has an n-type carrier with a sheet density carrier concentration of 10^17^ cm^−2^. To test the photoresponse of the device, two lasers with wavelengths of 473 nm and 808 nm were used, with varying power densities. In Figure 89a,b, the device’s time-resolved photoresponse was displayed under various light intensities and a bias voltage of 0.1 V. Impressively, the device demonstrated stable and reversible switching without any degradation in its performance due to light exposure. At the same time, the external laser source was turned off and on, indicating the superior multi-wavelength photoresponse performance of the 2D PbSe nanosheet. Figure 89c shows the corresponding photocurrents that are extracted from Figure 89a,b. It can be seen from Figure 89c that the photocurrent is dependent on light intensity. Jiang et al. [88] concluded that the concentration of photo-generated electron-hole pairs increased as the photon flux within a certain range of light intensity was absorbed more.

The variations of D* and R of 2D PbSe-based photodetector for two different light sources are shown in Figure 89d,e. R and D* both drop monotonically when light intensities rise from 6.50 to 25.85 mW/cm^2^ at 473 nm in wavelength. In this instance, the maximum R and D* values are 2.36 × 10^11^ Jones and 998.15 A/W, respectively. When the light intensity increases, the R and D* values for the 808 nm laser first increase and subsequently fall. In this instance, the maximum R and D* values are 2.31 × 10^11^ Jones and 970.05 A/W, respectively. 

Ren et al.’s [243] PVD technique was used to build PbSe thin films on a Si substrate to create photodiodes. They reported a detectivity of 1.2 × 10^11^ Jones and a maximum responsivity of 0.35 A/W. By adjusting the deposition period and applying an iodine treatment, Peng et al. [244] created large-area PbSe films made of varying-sized crystal particles on glass substrates using CBD. They reported that photodetectors based on them exhibited maximum responsivity. He et al. [245] fabricated photodetectors by depositing graphene and PbSe thin films on SiO_2_/Si substrates. A maximal responsivity of 420 A/W and a detectivity of 5.9 × 10^11^ Jones were reported. Che et al. [246] fabricated photodetectors by depositing spin-coated PbSe thin films with graphene on a SiO_2_/Si substrate. The device exhibited good photoelectric performance in the NIR range, as reported by the authors. At 36 mWcm^−2^, it demonstrated maximum responsivity. 

### 6.6. PbTe Applications

**Figure 90:**
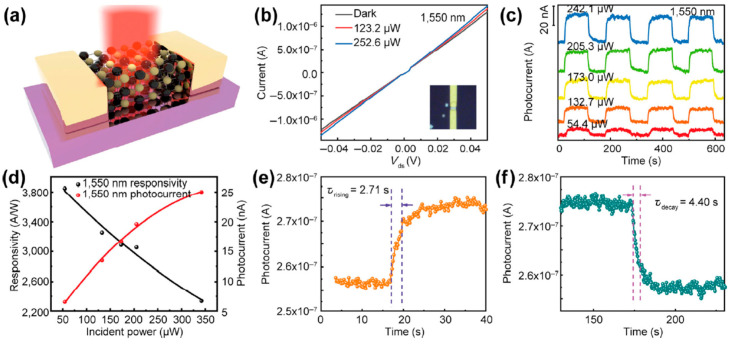
The PbTe nanosheet device’s optoelectronic characteristics. (**a**) Schematic illustration of a 2D PbTe-based FET-structured photodetector. (**b**) Measured in the dark and with varying incident power, the 2D PbTe phototransistor’s output characteristics. The device’s optical photograph is shown in the inset; the phototransistor’s channel length is 1.25 μm. (**c**) The 2D PbTe phototransistor’s time-resolved photo response at varying incident powers; 10 mV is the bias voltage. (**d**) The photocurrent and responsivity depend on the intensity of the incident’s power. (**e**,**f**) The device’s response and recovery time when the light source is turned on and off, as determined from the time-resolve photo response spectra. Using a 1550 nm laser, all experiments are carried out at room temperature. Reproduced from [86] under permissions from copyright clearance center.

Zhao et al. [86] synthesized ~7 nm thick PbTe thin films on fluoro phlogopite mica substrate by PVD method. Photodetectors were fabricated with Cr/Au electrodes. As seen schematically in Figure 90a, they built photodetectors based on 2D PbTe NSs on flexible mica substrates. The I–V curves of 2D PbTe photodetectors with a fixed wavelength of 1550 nm at room temperature are displayed in Figure 90b with varying incident laser optical strengths (from dark to 252.6 μW). The current rose in proportion to the incident light power. The optical microscope picture of the device is shown in the inset of Figure 90b. When the laser power is 242.1, 205.3, 173.0, 132.7, and 54.4 μW, respectively, Figure 90c displays the time-dependent photocurrent (I_ph_ = I_light_ − I_dark_, where I_ph_ is photocurrent and I_light_ and I_dark_ are the current under light and dark circumstances). Figure 90d illustrates the square-shaped photocurrent’s repeatability. They reported a high responsivity of 3847.1 A/W under 1550 nm laser illumination at incident power of 54.4 μW (Figure 90d), and the response and recovery time were 2.71, 4.40 s respectively (Figure 90e,f). As the incident optical power increases, R drops, and I_ph_ steadily increases to a saturated trend, as seen in Figure 90d. The properties of PbTe and the two-terminal devices’ small channel length (1.25 μm) determine the excellent responsivity. They determined the recovery time (τ_decay_) by measuring the photocurrent falling to 20% and the reaction time (τ_rising_) by measuring the photocurrent reaching 80% of the maximal value. Based on their observations from Raman and XPS characterizations, they concluded that the poor reaction and recovery time was most likely due to partial oxidation of the PbTe nanosheet surface during device manufacturing. They said that improving the device’s fabrication process and testing in a vacuum environment will shorten the time that PbTe NSs are exposed to air, improving response times. Surface passivation, 2D/electrode interface optimization, optimal heterostructure design, and other techniques may also help to further improve the 2D PbTe photodetector’s performance.

**Figure 91:**
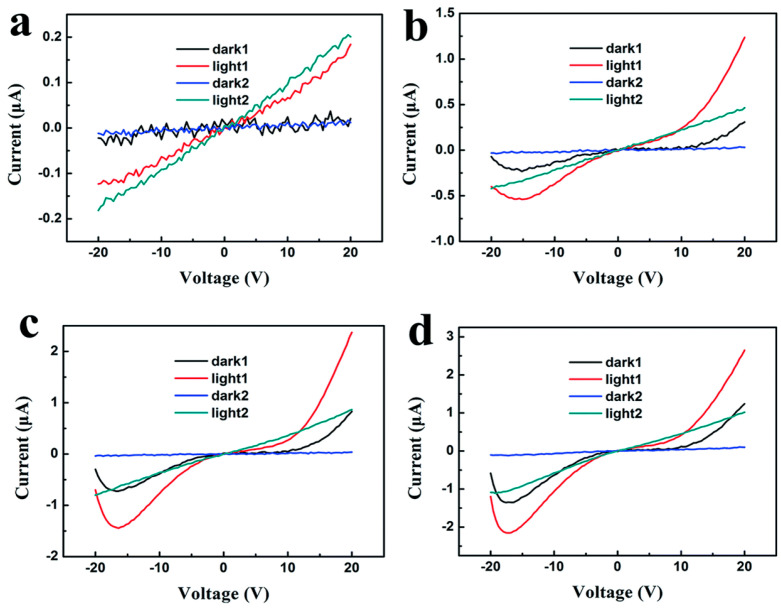
I–V curves for PbTe quantum dot film photodetectors treated with TBAI at varying thicknesses under xenon light illumination and without it are as follows: (**a**) 4 cycles at 47 nm, (**b**) 6 cycles at 68 nm, (**c**) 8 cycles at 76 nm, and (**d**) 12 cycles at 91 nm. Reproduced from [247] under permissions from copyright clearance center.

Lin et al. [247] synthesized PbTe quantum dot thin film by a layer-by-layer spin-coating method. These thin films were deposited on a SiO_2_/Si substrate with Ti/Au electrodes to fabricate photodetectors. They treated the PbTe quantum dot thin film by tetrabutylammonium iodide (TBAI) ligand exchange process. Figure 91a–d shows the *I–V* characteristics of the devices of various thicknesses. Figure 91 shows that, except for the film with a thickness of 47 nm, the nonlinear Schottky contact transforms into a symmetric linear Ohmic contact. 

**Figure 92:**
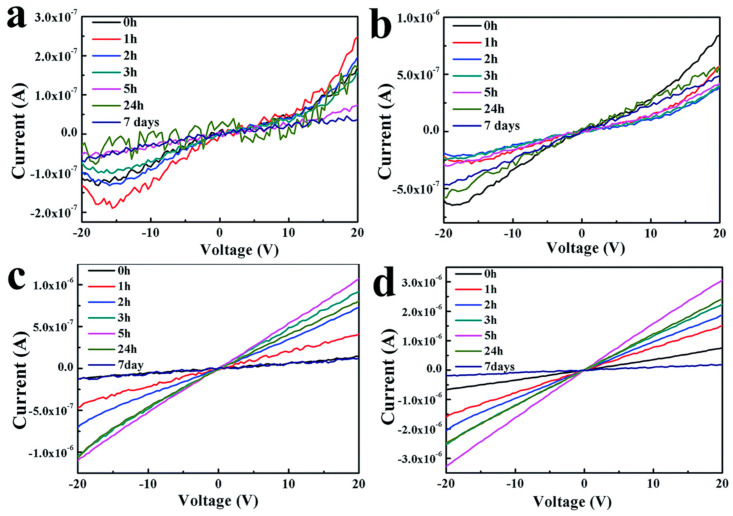
(**a**) Dark current of the photodetector with PMMA, (**b**) photocurrent of the photodetector with PMMA, (**c**) dark current of the photodetector without PMMA, and (**d**) photocurrent of the photodetector without PMMA. Reproduced from [247] under permissions from copyright clearance center.

Because of the increased number of photo-generated carriers brought on by increased light absorption, both the dark current and photocurrent increase with thickness. They stated that the TBAI treatment successfully improves the conductivity and stability of quantum dot films through surface passivation. They concluded that a barrier separates the high work function Au from the n-type TBAI-treated PbTe quantum dot film, indicating that the fresh sample for TBAI-treated PbTe quantum dot film possesses Schottky contact. The PbTe quantum dot film transitions from n to p-type due to surface oxidation, which results in the Ohmic type contact. To verify the conjecture, they performed stability tests on PbTe quantum dot film photodetectors with and without the polymethyl methacrylate (PMMA) layer. For photodetectors with PMMA protective layers, Figure 92a,b shows a nearly constant dark current from the start to seven days [247]. Additionally, it is evident that the photocurrent steadily decreases during the first five hours before increasing to a stable value after 24 h. For photodetectors without PMMA protection, Figure 92c,d shows progressively rising dark current and photocurrent from the start to five hours. After a day, it slightly decreased, and after seven days, when the photodetector was kept in the air, the photoresponse completely vanished. In contrast, photodetectors with PMMA protection can use the Schottky contact for an extended period of time; photodetectors without PMMA protection must use the Ohmic contact type from the start. As per their findings, the oxidized process is crucial for both charge injection and transportation processes because the presence of an oxidized state on the surface of PbTe quantum dots alters the barrier height between the electrode and film, thereby converting the contact from Schottky to Ohmic. Because the oxidized state is a form of p-type doping for PbTe quantum dot films, raising the carrier concentration and air exposure can also enhance the film conductivity. With increasing thin film thickness, they reported responsivity of 0.13 mA/W, 1 mA/W, 1.6 mA/W, and 1.9 mA/W for these photodetectors.

**Figure 93:**
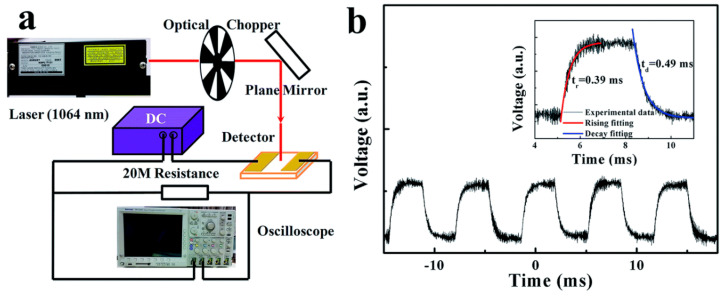
(**a**) Experimental setup for measuring the photodetector’s time-dependent photoresponse. (**b**) Time-dependent photoresponse in the presence of 150 Hz pulsing light. The response and recovery time are obtained by fitting exponential curves to the rising and decaying photocurrent processes, as shown in the inset for a single photoresponse cycle. Reproduced from [247] under permissions from copyright clearance center.

They used the experimental setup shown in Figure 93a to determine the repeatability of time-dependent photo response measurement. They found good photo response repeatability, as shown in Figure 93b. By fitting a single photo response cycle at a frequency of 150 Hz, they obtained a response time of 0.39 ms and a recovery time of 0.49 ms. 

Han et al. [248] synthesized waveguide-integrated PbTe photodetectors. PbTe thin film, Sn, and Ge_23_Sb_7_S_70_ were deposited on the Si substrate to fabricate the photodetector. They reported that this photodetector showed responsivity of ~1.0 A/W around 2.1–2.5 μm mid IR region. They reported the detectivity of the photodetector at 2250 nm to be 2 × 10^12^ Jones. However, Table 5 shows the properties of the optical detectors made from Sn/PbX (X = S, Se, Te) compounds.

The many uses of SnSe, SnS, SnTe, PbS, PbSe, and PbTe in thermoelectrics, photovoltaics, infrared detection, and other optoelectronic devices have been discussed in this section. Due to its advantageous optical and electrical properties, SnSe is receiving attention for its potential in low-cost, environmentally friendly solar cells. At the same time, SnSe stands out as a top option for thermoelectric energy conversion due to its high thermoelectric figure of merit. Conversely, SnTe is used in thermoelectric devices and infrared detectors, where its adjustable bandgap is quite helpful. Infrared photodetectors frequently employ lead-based chalcogenides, such as PbS, PbSe, and PbTe; PbS and PbSe are especially useful in the near-and mid-infrared region, respectively. PbTe is still one of the most popular materials for thermoelectric power generation because of its well-established thermoelectric characteristics, especially in high-temperature applications. These materials are essential for developing next-generation energy conversion and sensing technologies because of their versatility in thermoelectrics and optoelectronics.

## 7. Conclusions

In this review, we reviewed the synthesis, structure, optical, and TE characteristics and uses of layered Sn/Pb X (X = S, Se, Te) compounds, emphasizing monolayered compounds. To clarify the relationship between the different parameters that impact the optical and thermal conductivity properties of these materials, we presented the most recent theoretical and experimental data. The development of these earth-rich multilayer materials has advanced significantly over the past 10 years, showing encouraging optical and TE properties. Despite the significant advancements made in recent years, these qualities still require improvement to be used in optical detection and TE devices. First, the synthesis of these materials, monolayer in particular, is still in its infancy. MBE, CVD, ALD, PVD, liquid exfoliation, and ME are some of the present, difficult techniques for achieving 2D structures of these materials. These must be addressed to produce scalable and high-quality 2D IVA-VA composite material easily. There will be a big boost in the production of these materials if they can be synthesized like graphene using CVD. Sn and Pb-based materials’ thermoelectric qualities provide much promise for industrial uses, especially in energy harvesting and waste heat recovery. These materials are perfect for use in sectors like manufacturing and the automotive industry, which produce a lot of waste heat since they can turn heat into energy. By recovering energy that would otherwise be wasted, devices made of materials like SnSe and PbTe can improve energy efficiency and lessen their adverse environmental effects. These materials also show promise for wearable electronics and off-grid power generation in isolated areas where dependable, low-maintenance power solutions are required. TEGs based on Sn and Pb-based materials may provide sustainable energy sources for both small and large-scale applications. For commercial adoption, scalability and cost-efficiency issues must be resolved. Improving the long-term durability of these materials under operating circumstances, like high temperatures, and streamlining the production procedures are essential for their more comprehensive industrial application. Subsequent investigations have to concentrate on optimizing these substances for pragmatic implementation, guaranteeing their economic feasibility and longevity for practical usage.

## Figures and Tables

**Table 1:** Synthesis of 2D Sn/Pb X (X = S, Se, Te) using various methods.

Material	Morphology	Process Used	Thickness (nm)	Reference
SnSe	Nanoflakes	ME	>10	Song et al. [38]
SnSe	Nanoflakes	Micromechanical Cleavage	90	Cho et al. [28]
SnSe	Nanoflakes	Micromechanical Cleavage	~7 and 28	Yang et al. [27]
SnSe	Nanoflakes	ME	50	Guo et al. [30]
SnSe	Nanoflakes	ME	71	Yang et al. [29]
SnSe	Nanoflakes	ME	20.2–31.5	Liu et al. [31]
SnSe	Nanoflakes	ME	1.82	Liu et al. [32]
SnSe	2D films	LPE	N/A	Li et al. [58]
	Monolayer	LPE	N/A	
SnSe	NSs	LPE	3000 rpm: 8.9 8000 rpm: 5.9 12,000 rpm: 4.3	Huang et al. [33]
SnSe	NSs	LPE	3000 rpm: 9.5 8000 rpm: 6 12,000 rpm: 2.5	Ye et al. [34]
SnSe	NSs	LPE	8	Li et al. [59]
SnSe	NSs	Li Intercalation Assisted LPE	3.4	Ju and Kim [37]
SnSe	NSs	Li Intercalation Assisted LPE	6	Ren et al. [35]
SnSe	NSs	Li Intercalation Assisted LPE	0.68	Qiao et al. [36]
SnSe	NSs	Molecule intercalation method and LPE (sonication-assisted)	1.82	Doung et al. [39]
SnSe	Nanoflakes	Electrochemical and ME	2.7	Lee et al. [40]
SnSe	Nanoflakes	Vapor transport deposition and nitrogen etching	0.68	Jiang et al. [48]
SnSe	NSs	Chemical Vapor Transport and ME	6	Yu et al. [49]
SnSe	Nanoflakes	Sputtering	5	Hao et al. [51]
SnSe	Nanoflakes	PVD	~2.0	Chiu et al. [53]
SnSe	Nanoflakes	Vapor Transport Deposition and Nitrogen Etching	0.68	Jiang et al. [54]
SnSe	Nanoflakes	MBE	0.68	Zhou et al. [57]
SnSe	Monolayer	Van der Waals MBE	0.60	Chang et al. [56]
SnSe	Nanoflakes	Microwave-assisted solvothermal synthesis	N/A	Hartl et al. [58]
SnS	NSs	PVD	N/A	Zhang et al. [60]
SnS	NSs	LPE	4.1	Brent et al. [61]
SnS	Monolayer	Van der Waals Adhesion Exfoliation Method	0.7	Khan et al. [62]
SnS	NSs	N/A	N/A	Khan et al. [63]
SnS	NSs	Thermal Evaporation	Free of buffer layer-: 80 With Ag buffer layer-: 37 With Au buffer layer-: 51	Jamali-Sheini et al. [64]
SnS	NSs	Microwave-assisted Assisted Solvothermal Synthesis	N/A	Modi et al. [65]
SnS	Porous NSs	Chemical Exfoliation Process	N/A	Ju et al. [66]
SnS	NSs	PVD	12	Yuan et al. [67]
SnS	NSs	Laser ablation vapor transport process	N/A	Kang et al. [68]
SnS	NSs	Chemical Bath Deposition (CBD)	100	Shan et al. [69]
SnS	Nanoflakes	Thermal evaporation	5–160	Sutter et al. [70]
SnS	Nanoflakes	CVD	25–95	Liu et al. [71]
SnS	NSs	LPE	~4.5 and ~10	Sarkar and Stratakis [72]
SnS	NSs	LPE	1.10	Sarker et al. [42]
SnS	NSs	Solvothermal Process	10	Li et al. [73]
SnS	Nanoflakes	PVD	5.5	Xia et al. [74]
SnS	NSs	ME	~0.8 and ~1.8	Krishnamurti et al. [75]
SnS	Nanoflakes	ME	13.8, 10.5 and 4.3	Higashitarumizu et al. [76]
SnS	Nanoplates	Physical vapor transport	6	Tian et al. [77]
SnS	NSs	Magnetron Sputtering	11.2	Patel et al. [78]
SnS	NSs	Colloidal synthesis	7	Li et al. [79]
SnS	NSs	N/A	20	Zhang et al. [80]
SnTe	2D layers	DFT calculations	N/A	Chang et al. [26]
SnTe	Nanocrystals	Colloidal synthesis (hot injection)	30	Li et al. [81]
SnTe	2D layers	N/A	N/A	He et al. [82]
SnTe	2D Nanoplates	CVD	3.6	Liu et al. [83]
SnTe	NSs	N/A	N/A	Song et al. [84]
	Nanofilms	Magnetron Sputtering	N/A	
SnTe	NSs	Low-temperature STM and MBE	N/A	Chang et al. [85]
PbTe	NSs	PVD	7.1	Zhao et al. [86]
PbTe	NSs	Hydrothermal synthesis	N/A	Zhu et al. [87]
PbSe	NSs	Atmospheric pressure chemical vapor deposition (APCVD)	~19.6, ~47.6, and ~101	Jiang et al. [88]
PbSe	Nanoplates	N/A	2–4	Koh et al. [89]
PbSe	Nanoplates	Colloidal synthesis	0.8 ± 0.1	Klepig et al. [90]
PbS	NSs	Colloidal Synthesis	1.2–4.6	Bhandari et al. [91]
PbS	Nanoplatelets	LPE	3.7 and 11.2	Gao et al. [92]
PbS	NSs	Colloidal synthesis	N/A	Moayed et al. [93]
PbS	NSs	Oriented attachment of nanocrystals	4, 5.9, and 7	Aerts et al. [94]
PbS	NSs	Solvothermal method	25	Zhu et al. [95]
PbS	Nanoplates	CVD	5–35	Wen et al. [96]
PbS	NSs	2D Oriented attachment of nanocrystals	2.2	Schleicher et al. [97]
PbS	NSs	Colloidal synthesis (Precursor-based)	2.0 and 3.6	Zhang et al. [98]
PbS	Nanoplates	WCS	3.5	Wu et al. [99]
PbS	NSs	Bottom-up approach	1.8	Acharya et al. [100]
PbS	Nanoplatelets	Colloidal synthesis	1–2	Manteiga Vázquez et al. [101]
PbS	Nanoplates	Colloidal synthesis (single-molecule precursor approach)	1.8	Khan et al. [102]
PbS	NSs	Colloidal synthesis	2.2	áPeter Lu [103]
PbS	NSs	Colloidal synthesis	2.4	Premathilaka et al. [104]
PbS	Nanoplates	Hot-Injection	5	Sontangg et al. [105]
PbS	NSs	Colloidal synthesis	10 ± 0.2	Bielewicz et al. [106]
PbS	Nanoplates	Chemical synthesis	50	Han et al. [107]
PbS	NSs	One pot synthesis	<5	Shkir et al. [108]
PbS	NSs	Colloidal synthesis	1.2	Akkerman et al. [109]
PbS	NSs	CVD	50	Gu et al. [110]

**Table 2:** Phases of Sn/Pb X (X = S, Se, Te) multi and monolayer with their lattice type and constants.

Material Phase	Type of Lattice	Process Used for Synthesis/Calculation Method	Pressure (GPa)	Type of Film	Lattice Parameters a, b, c or a, b or a (Å)	Reference
α-SnSe	Orthogonal, Pnma	N/A	N/A	N/A	11.5524, 4.1777, 4.4261	[114]
β-SnSe	Orthogonal, Cnma	N/A	N/A	N/A	4.3062, 11.7243, 4.3178	[117]
α-SnSe	Orthogonal, Pnma	Arc Melting	N/A	Multilayer	11.5524, 4.1777 4.4261	[118]
β-SnSe	Orthogonal, Cmcm	Arc Melting with isothermal heating	N/A	Multilayer	4.3062, 11.7243, 4.3178	[118]
π-SnSe	Cubic, *P*2_1_3	N/A	N/A	Multilayer	11.9702	[119]
Rock salt SnSe	N/A	N/A	N/A	Monolayer	N/A	[122]
π-SnSe	Honeycomb	DFT, as implemented in the VASP	N/A	Monolayer	N/A	[121]
α-SnSe	Orthorhombic	N/A	N/A	Monolayer	3.95, 4.82	[121]
β-SnSe	2D hexagonal	N/A	N/A	Monolayer	3.78	[121]
SnSe	N/A	N/A	N/A	Nanosheet	N/A	[49]
Rock salt SnSe	Cubic, *Fm*$\bar{3}$*m*	DFT, as implemented in the VASP	N/A	Multilayer	5.71	[123]
α-SnSe	Orthorhombic, *P*_21_mn	ab initio DFT (PBE and HSE)	N/A	Monolayer	a = 3.95, b = 4.82	[124]
β-SnSe	Hexagonal, *P*_3_m1	ab initio DFT (PBE and HSE)	N/A	Monolayer	3.78, 3.78	[124]
γ-SnSe	Honeycomb, *P*_21_mn	ab initio DFT (PBE and HSE)	N/A	Monolayer	3.78, 6.11	[124]
δ-SnSe	Honeycomb, *P*_21_ca	ab initio DFT (PBE and HSE)	N/A	Monolayer	6.14, 6.23	[124]
ε-SnSe	Honeycomb, *P*_21_ca	ab initio DFT (PBE and HSE)	N/A	Monolayer	7.1, 6.6	[124]
α-SnSe	N/A	N/A	N/A	Multilayer	11.32, 4.05, 4.24	[125]
β-SnSe	N/A	N/A	N/A	Multilayer	4.148, 11.480, 4.177	[125]
π-SnS	N/A	N/A	N/A	N/A	11.595	[124]
α-SnS	Orthogonal, Pnma	N/A	N/A	Multilayer	11.32, 4.05, 4.24	[126]
β-SnS	Orthogonal, Cmcm	N/A	N/A	Multilayer	4.148, 11.480 4.177	[126]
α-SnTe	rhombohedral or orthorhombic	N/A	N/A	N/A	a = 6.235 Å and α = 89.895 Å	[127]
β-SnTe	rhombohedral or orthorhombic, *Fm*$\bar{3}$*m*	N/A	Room temperature and pressure	N/A	a = 6.318 Å and α = 90 Å	[85]
γ-SnTe	rhombohedral or orthorhombic	N/A	N/A	N/A	N/A	[127]
Rock salt SnS	Cubic, *Fm*$\bar{3}$*m*	Epitaxial growth at high-pressure	N/A	Multilayer	5.80	[26]
π-SnS	Cubic, *P*2_1_3	Chemical Synthesis	N/A	Multilayer	11.595	[125]
α-SnTe	rhombohedral or orthorhombic, R3m	Arc Melting	N/A	Multilayer	a = 6.235 and α = 89.895	[128]
β-SnTe	Cubic, Fm$\bar{3}$m	Arc Melting with isothermal heating	>18 kbar	Multilayer	a = 6.318 and α = 90	[128]
γ-SnTe	Orthorhombic, Pnma	DFT, as implemented in the VASP	18 kbar	Multilayer	11.95, 4.37, 4.48	[128]
A and β-SnTe	N/A	N/A	N/A	Ultrathin	N/A	[129]
γ-SnTe, Rock salt	N/A	Variable temperature scanning tunneling microscopy (VT-STM)	N/A	Nanoplate	N/A	[129]
PbS-Rock salt	Cubic, *Fm*$\bar{3}$*m*	N/A	N/A	N/A	5.936	[130]
B1-PbS to B33-PbS	Orthorhombic	N/A	2.5 GPa	N/A	N/A	[131]
PbS	-Pnma -Orthorhombic	N/A	3.0 GPa	-Nanocrystal -Nanofilms	10.96, 4.02, 4.28	[132]
Bulk PbS	N/A	First-principles calculations using the projector augmented plane waves method	N/A	Monolayer	6.00	[133]
B1-PbS	Cubic, *Fm*$\bar{3}$*m*	CGA	Normal pressure	Multilayer	a = 6.12	[132,134,135]
PbS-Orthorhombic	Pnma	CGA	~9.5 GPa	Multilayer	11.18, 4.17, 4.05 [Pnma] 4.06, 10.85, 4.16 [Cmcm]	[136]
B2-PbS	cubic, *Pm*$\bar{3}$*m*	CGA	>16 GPa	Multilayer	a = 3.56	[137]
B1-PbSe	Cubic, *Fm*$\bar{3}$*m*	Arc Melting	N/A	Multilayer	a = 5.936	[132]
B33-PbSe	Orthorhombic, Pnma	Chemical synthesis after one hour	N/A	Multilayer	10.96, 4.02, 4.28	[137]
PbSe	Orthorhombic, Pnma	N/A	~9.5 GPa	N/A	11.18, 4.17, 4.05	[134]
CsCl PbSe	Orthorhombic, Cmcm	N/A	~9.5 GPa	N/A	3.56	[134]
Honeycomb Puckered PbSe	*P3m1*	DFT calculation	Normal pressure	Monolayer	*a* = *b* = 4.091	[136]
B1-PbTe	Cubic, *Fm*$\bar{3}$*m*	Chemical Synthesis	Normal pressure	Multilayer	a = 6.461	[138]
PbTe	Pnma Pmm CsCl	N/A	N/A	N/A	N/A	[139]
Orthorhombic PbTe	Pnma	GGA	~9.5 GPa	Multilayer	8.11, 4.5, 6.26	[140]
B2-PbTe	cubic, *Pm*$\bar{3}$*m*	GGA	>24.7 GPa	Multilayer	a = 3.57	[140]

**Table 3:** TE properties of Sn/Pb X (X = S, Se, Te).

Material	Method	Type of Layer	zT Value	Temperature	PF (Power Factor)	Reference:
SnS	DFT and Boltzmann	Monolayer	1.36–5	300–600 K	N/A	Gupta et al. [164]
SnS	WCS	NSs	0.8	873 K	0.48	Li et al. [165]
SnS	Synthesized porous and coated with PANI	NSs	0.078	450 K	N/A	Ju et al. [66]
SnS	First-Principle Calculations and Boltzmann Transport	Monolayer	N/A	N/A	~1.1 W-m^−1^-K^−2^	Gupta et al. [167]
SnS SnSe	DFT and Boltzmann transport	2D monochalcogenides	1.75, 1.88 2.63, 2.46	700 K	N/A	Shafique et al. [168]
SnS	Density functional theory and semi-classical Boltzmann transport	Monolayer	1.04	700 K	N/A	Pandit et al. [169]
SnS	First principal calculation	Monolayer	0.13–0.89	300–700 K	N/A	Fang et al. [170]
SnS	Green’s function-based transport	Monolayer	ZigZag-0.95 Armchair-1.6	Room temperature	N/A	Sandonas et al. [179]
SnS	USPEX and VASP	Monolayer	~1.19–3.18	300–900 K	N/A	Dong et al. [172]
SnTe	Density functional theory and semi-classical Boltzmann transport	Monolayer	n-type: 2.9 p-type: 2.2	N/A	37 mW/(m K^2^)	Li et al. [173]
SnTe	Phonon dispersion calculation and elasticity criteria	Monolayer	~0.96–3.81	300–900 K	N/A	Dong et al. [172]
SnTe	First-principles calculation and Boltzmann transport	Monolayer	1.58	900 K	N/A	Zhang et al. [174]
SnTe	First-principles calculation and Boltzmann transport	Monolayer	1.44	900 K	N/A	Tang et al. [175]
SnTe	First-principles calculation and Boltzmann transport	Monolayer	2.61	500 K	N/A	Liu et al. [176]
SnTe	DFT and Boltzmann transport	Monolayer and bilayer	>1.6	900 K	N/A	Lubis et al. [177]
SnTe	First-principle calculations	Bilayer	3.48	700 K	N/A	Pandit et al. [178]
SnTe	DFT and semi-classical Boltzmann transport	Monolayer	1.46	700 K	N/A	Pandit et al. [169]
SnTe	Synthesized porous	NSs	1.1	923 K	N/A	Ju and Kim [179]
SnTe	Vacuum deposition method	Thin films	~1.0	300 K	N/A	Panwar et al. [180]
SnSe	N/A	Single crystal	2.6	973 K	N/A	Zhao et al. [182]
SnSe	Hydrothermal synthesis and SPS	Nanoplates	~2.1 ~1.75	873 K	N/A	Chandra et al. [183]
SnSe	Synthesized porous by solution synthesis	NSs	0.8 to ~1.86	873 K	N/A	Li et al. [184]
SnSe	First principle calculations	Single sheet Single sheet	3.27 N/A	700 K room temperature	2.57 W/mK (zigzag) and 2.02 W/mK (armchair)	Wang et al. [185]
SnSe	USPEX combined with VASP	Monolayer	~0.93 to 2.51	300 K to 900 K	N/A	Dong et al. [172]
SnSe	DFT combined with Boltzmann transport theory	Monolayer	2.63 and 2.46	700 K	N/A	Shafique et al. [168]
SnSe	N/A	Monolayer	N/A	-Room temperature	0.91–0.97 W-m^−1^-K^−2^	Fang et al. [170]
	N/A	Monolayer	~0.25–1.41	300–700 K	N/A	
SnSe	First-principles electronic structure calculations and Boltzmann transport theory	Monolayer	p-type: 1.2 n-type: 2.5	N/A	N/A	Ding et al. [186]
SnSe	Ge-doped synthesized by simple hydrothermal route followed by SPS	2D nanoplates	~2.1	873 K	N/A	Chandra et al. [183]
	Ge doping (3 mol%)	Nanoplates	~1.75	873 K	N/A	
	N/A	Nanoplates	N/A	873 K	~0.18 W/mK	
SnSe	First-principles calculations, semi-classical Boltzmann, with SOC	Monolayer	N/A	N/A	N/A	Guo and Wang [187]
		Monolayer	N/A	N/A	N/A	
β−SnSe	DFT	Monolayer	2.06	300 K	N/A	Hu et al. [122]
		2D hexagonal	2.32	700 K	N/A	
PbS	Layer-by-layer method via ligand exchange	Nanocrystal films	0.30	405 K	N/A	Ding et al. [189]
	Layer-by-layer method via ligand exchange	Nanocrystal	0.30	405 K	N/A	
PbS	Ab initio approach	Monolayer	~1.0	N/A	6.1 × 10^10^ W/mK^2^	Haq et al. [190]
PbS	DFT combined with semi-classical Boltzmann transport theory	2D monolayers	n-type: 1.51	700 K	N/A	Pandit et al. [169]
PbSe	p-type doped	Monolayer	p-type: ~1.3	900 K	N/A	Tang et al. [136]
PbSe	DFT and p + n doped	N/A	0.3 and 0.8	Room temperature	N/A	Yin et al. [191]
	DFT and p + n doped	N/A	2.2	900 K	N/A	
PbSe	Doped p and n dopants	N/A	≤0.1 to 0.3	Room temperature	N/A	Gayner et al. [192]
	Doped p and n dopants	N/A	≥1	650–873 K	N/A	
PbTe	N/A	N/A	~2.5	923 K	~1.5 × 10^20^ cm^−3^	[193]
PbTe	First-principles calculations and semi-classical Boltmazz Transport	Monolayer	~1.55	900 K	N/A	[203]
PbTe	First-principles calculations and semi-classical Boltzmann transport theory	Monolayer	>1.58	900 K	N/A	Zhang et al. [174]
SnTe	First-principles calculations and semi-classical Boltzmann transport theory	Monolayer	N/A	N/A	N/A	Liu et al. [176]
	First-principles calculations and semi-classical Boltzmann transport theory	Monolayer	5.91	500 K	N/A	
PbTe	DFT combined with semi-classical Boltzmann transport theory and SOC	2D monolayer	1.94	700 K	N/A	Pandit et al. [169]

**Table 4:** Optical properties of Sn/Pb X (X = S, Se, Te).

Material	Type of Lattice	Process Used for Synthesis/Calculation Method	Type of Film	zT-Value and Temp.	Optical Bandgap	Reference
SnS	Monolayer	DFT, Boltzmann transport equation	Thin film	~5 and ~1.36 at 600 K and 300 K	N/A	[165]
SnS	Nanosheet	WCS	Thin film	N/A	N/A	[66]
SnS	NSs	Coated with conductive PANI	Thin film	N/A	N/A	[166]
SnTe	NSs	Synthesized porous	Thin film	1.1 at 923 K		
SnS	Monolayer	Piezoelectric nanogenerators and PFM	Thin film	N/A	N/A	[64]
SnS SnSe	2D heterojunction	First-principle calculations and DFT	Thin film	N/A	1.3 eV	[167]
SnS	Monolayer	First-principle calculations and Boltzmann transport equation	Thin film	N/A	N/A	[168]
SnS	2D monochalcogenides	DFT and Boltzmann transport theory	Thin film	1.75, 1.88 at 700 K	N/A	[169]
SnSe	Monolayer	DFT and Boltzmann transport theory	Thin film	2.63, 2.46 at 700 K	N/A	
SnS	2D monolayers	DFT and semi-classical Boltzmann transport theory	Thin film	1.04 at 700 K	N/A	[170]
SnS	Monolayer	First-principle calculations	Thin film	~0.13–0.89 at 300–700 K	N/A	[179]
SnSe	Monolayer	N/A	N/A	~0.25–1.41 at 300–700 K	N/A	
SnS	Monolayer	Green’s function-based transport techniques	Thin film	Zigzag~0.95 Armchair~1.6	N/A	[172]
SnS	Monolayer	USPEX combined with VASP	Thin film	~1.19 to 3.18 at 300K to 900 K	N/A	[173]
SnTe	Monolayer	Phonon dispersion calculation	Thin film	~0.96 to 3.81 at 300K to 900 K	N/A	
SnSe	Monolayer	USPEX combined with VASP	Thin film	~0.93 to 2.51 at 300K to 900 K	N/A	
SnTe	monolayer	DFT, Boltzmann transport theory	Thin film	2.9 (n-type, armchair), 2.2 (p-type, armchair)	1.05 eV	[174]
SnTe	Monolayer	First-principles calculation, Boltzmann transport theory	Thin film	More than 1.58 at 900 K	N/A	[175]
PbTe	Monolayer	First-principles calculation, the Boltzmann transport theory	N/A	~1.58 at 900 K	N/A	
SnTe	2D wrinkled monolayer	First-principles calculations, Boltzmann transport theory	Thin film	1.44 at 900 K	N/A	[176]
SnTe	Monolayer	First-principles calculations, Boltzmann transport equation	Thin film	~2.61 at 500 K	indirect band gap semiconductors	[177]
PbTe	Monolayer	First-principles calculations, Boltzmann transport equation	N/A	~5.91 at 500 K		
SnTe	Monolayer Bilayer	DFT, Linearised Boltzmann transport theory	Single-layer thickness	>1.6 at 900 K	N/A	[178]
SnTe	Bilayer	First-principle calculations	Thin film	3.48 at 700 K	N/A	[169]
SnTe	Monolayer	DFT, semi-classical Boltzmann transport theory	Thin film	1.46 at 700 K	N/A	[180]
PbS	2D monolayers	DFT, semi-classical Boltzmann transport theory	N/A	n-type: 1.51 at 700 K	N/A	
PbTe	2D monolayers	DFT, Semi-classical Boltzmann Transport Theory	N/A	-p-type: 1.94 at 700 K	N/A	
SnTe	N/A	Vacuum Deposition Method	Thin film	~1.0 at 300 K	N/A	[181]
SnSe	Monolayer	N/A	N/A	N/A	N/A	[182]
SnSe	Single crystal	N/A	N/A	2.6 at 973 K	N/A	[183]
SnSe	Nanoplates	Hydrothermal Synthesis Anisotropic Measurement Parallel to the SPS	N/A	N/A	N/A	[184]
SnSe	N/A	N/A	Thin film	0.055 at 501 K	N/A	[185]
SnSe	Single sheet	First-Principle Calculations	N/A	3.27 at 700 K	N/A	[186]
SnSe	Monolayer	First-principles calculations, Boltzmann transport theory	N/A	1.2 (p-type), 2.5 (n-type)	N/A	[183]
SnSe	2D nanoplates	Hydrothermal synthesis, SPS	N/A	~2.1 at 873 K ~1.75 at 873 K	N/A	[187]
SnSe	Monolayer	First-principles calculations, semi-classical Boltzmann transport theory, SOC	N/A	N/A	N/A	[188]
SnS	Monolayer					
SnSe	Monolayer	Ab initio DFT calculations	N/A	β−SnSe: 2.06 at 300 K	N/A	[121]
SnSe	2D hexagonal	First-principle study	N/A	2.32 at 700 K	N/A	[189]
Pbs PbTe	N/A	Layer-by-layer method via ligand exchange	Nanocrystal films	0.30 at 405 K	N/A	[190]
PbSe	Monolayer	N/A	N/A	1.3 (p-type, zigzag) at 900 K	N/A	[114]
PbSe	2D	First-principles calculations	N/A	3.95 at 500 K	N/A	[191]
PbSe	N/A	DFT	N/A	0.8 (p-type, room temperature), 0.3 (n-type, room temperature), 2.2 (p-type, 900 K)	N/A	[192]
PbSe	N/A	N/A	N/A	≤0.1 to 0.3 (room temperature), ≥1 (650–873 K)	N/A	[193]
PbTe	2D	Doped with n and p-type impurities	N/A	n and p-type: ~2.5 at 923 K	N/A	[194]
PbTe	N/A	Band convergence	N/A	N/A	N/A	[195]
PbTe	N/A	Band convergence	N/A	N/A	N/A	[196]
PbTe	N/A	Introducing an excess density of state near Fermi level and increasing energy-dependence (*μ*E)	N/A	N/A	N/A	[197]
PbTe	Nanostructures	Introducing foreign atom doping	N/A	N/A	N/A	[202]
PbTe	Monolayer Bilayer Trilayer Tetralayer	First-principles calculations, DFT	N/A	N/A	Bandgap increases with a decrease in layer thickness 0.4 eV 0.25 eV 0.2 eV	[203]
PbTe	Honeycomb-like puckered monolayer	First-principles calculations, the semi-classical Boltzmann transport theory	N/A	~1.55 at 900 K	Wide bandgap of 2.251 eV	[203]
SnSe	Nanolayers	Typical bath sonication exfoliation method	8.9,5.9, and 4.3 nm corresponding to 15, 10, 7 layers	N/A	Indirect bandgap: 0.91, 1.13, 1.27, and 1.35 eV Calculated bandgap: 0.93 to 1.79 eV	[15]
SnSe	Monolayers	PBE method and HSE06 calculations	N/A	N/A	A: 1.04 eV Β: 2.22 eV Γ: 1.52 eV δ: 1.55 eV	[121]
SnSe	Monolayer	GW with BSE method	N/A	N/A	1.41 eV	[204]
SnSe	Monolayer	DFT calculation	N/A	N/A	0.986, 1.067, 1.471, 1.67 eV	[205]
SnS	Monolayer	First-principles calculations	N/A	N/A	1.63 eV	[208]
SnS	Monolayer	First-principles calculations, DFT	N/A	N/A	1.38 eV	[209]
SnS	Monolayer	First-principles calculations	200 nm	N/A	2.89 eV	[86]
SnS	NSs	UV–vis–NIR Fractioned SnS sol B	N/A	N/A	1.03 eV 1.65 eV	[62]
SnTe	Hexagonal monolayer	Ab initio approach	N/A	N/A	PBE theory: ~2.00 eV HSE06: ~2.60 eV	[210]
SnX (X = S, Se, Te)	Monolayer	N/A	N/A	N/A	Indirect Bandgap	[190]
PbS	NSs	CBD	N/A	N/A	1.14, 1.39, 1.43, 1.57, 1.64, 1.74 eV	[211]
PbS (001)	Monolayer	HSE + SOC	Hex-layer	N/A	N/A	[212]
			Few layers	N/A	0.24 to 1.92	
			Kf layer	N/A	N/A	
PbSe	Monolayer	DFT calculations, first-principle-based typical medium approximation	N/A	N/A	0.21 eV	[218]
PbSe	Monolayers	First-principles calculations within DFT full-potential linearized-augmented-plus-local-orbit method within DFT WIEN2k computational package	N/A	N/A	Indirect Bandgaps: 0.45, 1.39, and 1.26 eV	[219]
PbTe	Monolayer	DFT Calculations adopting the GCA of the PBE functional	N/A	N/A	1.742 eV Mono, bi, tri, and bulk: 1.23–3.20 eV	[220]
PbSe PbTe	N/A	SOC	N/A	N/A	2 eV	[221]
SnSe	NSs	Typical bath sonication method	N/A	N/A	0.91, 1.13, 1.27, and 1.35 eV	[33]
SnSe	Monolayer	BSE method	N/A	N/A	1.41 eV	[15]
SnSe	Monolayer	GCA, SCAN, HSE06, and other DFT methods	N/A	N/A	0.986, 1.067, 1.471, 1.67 eV	[204]
SnS	Monolayer	First-principles calculations	N/A	N/A	1.63 eV	[205]
SnS	Monolayer	N/A	N/A	N/A	1.3 eV	[166]
SnS	Monolayer	N/A	N/A	N/A		[205]
SnS	Monolayer	First-principle calculations based on DFT	N/A	N/A	1.38 eV	[222]
SnS	Monolayer	First-principle calculations	N/A	N/A	2.89 eV	[86]
SnS	NSs	UV–vis–NIR absorbance spectroscopy	Thin film	N/A	1.03 eV	[61]
SnS	Monolayer	First-principle calculations	200 nm	N/A	3.75 eV	[206]
SnTe	Hexagonal Monolayer	PBE theory and HSE06	N/A	N/A	PBE theory: ~2.00 eV HSE06: ~2.60 eV	[207]
SnX (X = S, Se, Te)	Monolayer	GW-BSE method	N/A	N/A	N/A	[208]
PbS	NSs	CBD	N/A	N/A	1.14, 1.39, 1.43, 1.57, 1.64, 1.74 eV	[209]
PbS	Monolayer to hex-layer	HSE + SOC method	N/A	N/A	0.24 to 1.92 eV	[133]
PbSe	Monolayer	DFT calculations with First-principles-based typical medium approximation	N/A	N/A	0.21 eV	[210]
PbSe	Monolayer	First-principles calculations within DFT	N/A	N/A	α-0.45 β-1.39 γ-1.26	[190]
PbTe	Monolayer	First-Principle Calculations	N/A	N/A	0.4 eV: bilayer 0.25 eV: trilayer 0.2 eV: tetralayer	[202]
PbTe	Monolayer	GCA implemented in the VASP	N/A	N/A	1.742 eV	[211]
PbTe	Mono, bi, tri, and bulk	First-principle calculations	N/A	N/A	1.23–3.20 eV	[212]

**Table 5:** Properties of the optical detectors made from Sn/PbX (X = S, Se, Te) compounds.

Material	Maximum Responsivity	Maximum Detectivity	Excitation Wavelength and Power	Rise and Fall Time	Spectral Range	Reference
SnSe	405, 532, and 785 nm wavelengths as 1.75 × 10^4^ A/W, 4.63 × 10^3^ A/W, and 1.52 × 10^3^ A/W	N/A	N/A	N/A	N/A	Li et al. [51]
SnSe NSs synthesized magnetron sputtering method	277.3 A/W	7.6 × 10^11^ Jones	UV-visible-NIR range	N/A	UV to NIR	Hao et al. [52]
SnSe NSs synthesized by hot injection method	30 mA/W (400 nm)/11 mA/W (1050 nm)	N/A	400 nm (0.46 mW/cm^2^)/1050 nm (0.4 mW/cm^2^)	N/A	Visible to NIR	Zhong et al. [218]
SnSe NSs synthesized by electron beam lithography	~330 A/W	N/A	White light, bias voltage of 0.1 V	N/A	N/A	Zhao et al. [219]
SnSe NSs	9.27 A/W	4.08 × 10^10^ Jones	808 nm	N/A	360 to 1550 nm	Li et al. [166]
SnSe thin film photodetectors by depositing SnSe thin films on PET templates	1745.5 A/W (404 nm)/0.16 A/W (10.6 µm)	4.2 × 10^11^ Jones (404 nm)/3.9 × 10^7^ Jones (10.6 µm)	404 nm laser/10.6 µm laser	N/A	UV to IR	Xu et al. [220]
SnS nanoflakes	N/A	N/A	N/A	Fast response and recovery time	N/A	Hu et al. [221]
SnS nanosheet-based photodetector (monolayer) SnS nanosheet-based photodetector (Au-decorated)	365 A/W 635 A/W	5.70 × 10^4^% 9.92 × 10^4^%	808 nm, 40 mW/cm^2^ 808 nm, 40 mW/cm^2^	Response: 0.35 s, Recovery: 0.35 s N/A	NIR NIR	Zhang et al. [60]
SnS nanoflakes synthesized by CVD	156.0 A/W	4.77 × 10^4^%	N/A	N/A	N/A	Liu et al. [71]
SnS nanoflakes fabricated by poly-ethylene terephthalate substrates	1280 A/W (355 nm)/69 A/W (1550 nm)	3.02 × 10^11^ Jones	355 nm/1550 nm	N/A	355 nm to 1550 nm	Dong et al. [223]
SnS NSs synthesized by PVD	161 A/W	4.45 × 10^4^%	450 nm blue light	N/A	N/A	Wen et al. [224]
SnS synthesized by metallic liquid tin	927 A/W (660 nm, single unit cell)/3.51 × 10^3^ A/W (660 nm, multiple unit cells)	1.09 × 10^9^ Jones (660 nm, single unit cell)/6.83 × 10^10^ Jones (660 nm, multiple unit cells)	280–850 nm	0.12 ms (single unit cell)/0.16 ms (multiple unit cells)	Deep UV to NIR	Krishnamurthi et al. [75]
SnS and SnS2 nanoflakes synthesized by phase-controlled synthesis	3390 mA/W	1.1 × 10^10^ Jones	N/A	3.10 ms (response)/1.59 ms (recovery)	N/A	Luo et al. [225]
SnS NSs synthesized by solvothermal process	86.2 mA/W	10^10^ Jones	Low intensity (0.02 mW/cm^2^)	150 ms	N/A	Modi et al. [65]
SnS films	N/A	N/A	365 nm, 80 µWcm^−2^	τg: 60 ms, τd: 10 ms	Broadband	Wang et al. [226]
SnS films	Up to 1.17 A/W (980 nm)/17.31 mA/W (1550 nm)	N/A	980 nm/1550 nm	11.0 ms	Visible to NIR	Li et al. [227]
Sns layers grown vertically on Si substrates	12 mA/W	~3.2 × 10^14^ Jones	760 nm, 7 mWcm^−2^	Rise: ~12 µs, Decay: ~55 µs	UV to NIR	Kumar et al. [228]
SnS NSs synthesized by the LPE method	59.8 μA/W	9.43 × 10^7^ Jones	N/A	0.1–0.3 s	UV to Visible	Huang et al. [229]
SnS films synthesized by the PVD method	14.78 µA/W	N/A	N/A	N/A	UV to visible	Dong et al. [230]
SnS	0.19 A/W	9.213 × 10^11^ Jones	1030 nm	Response: 2 s, Recovery: 4 s	UV to NIR	Vinoth et al. [231]
SnS NSs synthesized by the PVD method	1604 A/W	3.42 × 10^11^ Jones	850 nm	Response: 7.6 ms, Recovery: 29.9 ms	NIR	Yuan et al. [67]
SnS nanoflakes synthesized by CBD	0.01 mA/W	N/A	530 nm	Rise: 0.36 s, Decay: 0.38 s	UV to NIR	Mahdi et al. [232]
SnS	1652.87 A/W	8.05 × 10^12^ Jones	850 nm	Response: 6.5 ms	Visible to NIR	He et al. [233]
SnS thin films fabricated by a thermal evaporation method	6.4 × 10^2^ A/W	6.05 × 10^9^ Jones	N/A	Response: 1.5 s, Recovery: 2.5 s	N/A	Balakarthikeyan et al. [234]
SnS thin crystals synthesized by solvothermal method	2040 A/W	~3 × 10^9^ Jones	~4.75 × 10^5^%	~90 ms	N/A	Wang et al. [235]
SnS layers on n-Si substrates fabricated photodetectors	0.25 A/W (850 nm, −1 V bias)/1.19 A/W (10 µW/cm^2^)	1.3 × 10^11^ Jones (850 nm, −1 V bias)/7.1 × 10^11^ Jones (10 µW/cm^2^)	850 nm/850 nm	Rise: 41 µs/222 µs	NIR	Patel et al. [236]
SnTe nanoplates are synthesized by the van der Waals growth process and then deposited on mica substrates	698 mA/W (3.6 nm thick)/1.468 A/W (35 nm thick)	3.89 × 10^8^ Jones	980 nm	N/A	NIR	Liu et al. [83]
SnTe nanofilms synthesized by magnetron sputtering technique on a quartz substrate	1.71 A/W	3.46 × 10^11^ cmHz^1/2^W^−1^	940 nm, 0.2 mWcm^−2^	N/A	UV to NIR	Song et al. [84]
SnTe NSs synthesized by MBE on SrTiO_3_ substrate	3.75 A/W (2003 nm)	N/A	405 nm, 632 nm, 808 nm, 1550 nm, 2003 nm	N/A	Visible to mid-infrared	Jiang et al. [237]
SnTe thin films synthesized by a magnetron sputtering method deposited on polyethylene terephthalate templates used Pd as electrodes	3.9 mA/W	1.3 × 10^10^ Jones	404 nm	Response: 78 ms, Recovery: 84 ms	UV to MIR	Liu et al. [238]
SnTe nanoflakes deposited by depositing 100 nm to 120 nm thick SnTe nanoflakes on Si/SiO_2_ substrates with Cr/Au as electrodes	71.11 A/W (254 nm)/49.03 A/W (635 nm)/10.91 A/W (1550 nm)/4.17 A/W (4650 nm)	N/A	254 nm, 635 nm, 1550 nm, 4650 nm	N/A	Deep UV to MIR	Yang et al. [239]
PbS photodetectors by fabrication on PET substrate under dark and illuminated conditions	0.013 A/W (100 μm gap)/0.1 A/W (10 μm gap)	1.9 × 10^9^ Jones (100 μm gap)/1.3 × 10^9^ Jones (10 μm gap)	400–750 nm, 100 mWcm^−2^	N/A	Visible	Akkerman et al. [109]
PbS NSs by CVD method	7.5 A/W	1.44 × 10^12^ Jones	450 nm, 40 mW/cm^2^	~0.25 s	Visible	Wang et al. [240]
PbS nanoplates by CVD method	37 A/W to 119 A/W	N/A	N/A	N/A	N/A	Gu et al. [110]
PbS nanoplates with Cu2S residues by Cu_2_S residues on Si/SiO_2_ substrate and used Au as electrode	~1739 A/W	2.55 × 10^11^ Jones	808 nm, 0.5 mW	N/A	NIR	Wu et al. [99]
PbS thin layer by synthesization on a glass substrate	70 mA/W	1.8 × 10^11^ Jones	N/A	Response: 6.4 ms, Recovery: 15.6 ms	NIR	Thabit et al. [241]
PbS films on paper by spray pyrolysis method and graphite electrolyte used on them	0.0356 A/W	N/A	N/A	Response: 14.7 ms, Recovery: 6.3 ms	NIR	Khandoz et al. [242]
PbS NSs by LPE on ITO substrates	27.81 mA/W	3.96 × 10^10^ Jones	N/A	N/A	UV-Vis-NIR	Gao et al. [92]
PbSe NSs	~998.15 A/W (473 nm)/~970.05 A/W (808 nm)	~2.36 × 10^11^ Jones (473 nm)/~2.31 × 10^11^ Jones (808 nm)	473 nm, 6.50–25.85 mW/cm^2^/808 nm, variable	N/A	NIR	Jiang et al. [88]
PbSe thin films by PVD	0.35 A/W	1.2 × 10^11^ Jones	N/A	N/A	NIR	Ren et al. [243]
PbSe films by using the CBD	30.27 A/W	N/A	808 nm, 233 µW/cm^2^	N/A	NIR	Peng et al. [244]
PbSe thin films by depositing graphene and PbSe thin films on SiO_2_/Si substrates	420 A/W	5.9 × 10^11^ Jones	N/A	N/A	NIR	He et al. [245]
PbSe thin films by depositing spin-coated PbSe thin films with graphene on SiO_2_/Si substrate	1.1 × 10^4^ A/W	1.3 × 10^10^ Jones	NIR, 36 mWcm^−2^	Response: 7 ms, Recovery: 10 ms	NIR	Che et al. [246]
PbTe 2D thin films by PVD method	3847.1 A/W	N/A	1550 nm, 54.4 μW	2.71 s	4.40 s	Zhao et al. [86]
PbTe quantum dot thin film by a layer-by-layer spin-coating method	0.13–1.9 mA/W (depending on thickness)	N/A	N/A	0.39 ms	0.49 ms	Lin et al. [247]
PbTe thin films	~1.0 A/W	2 × 10^12^ Jones	2.1–2.5 μm	N/A	N/A	Han et al. [248]

## Data Availability

No new data were produced as part of this publication. We used data, Figures etc., from other sources with their permissions.
